# Monovalent Nondegrading Molecular Glues: An Updated Overview of Emerging Mechanisms and Therapeutic Development

**DOI:** 10.3390/ph19091388

**Published:** 2026-09-02

**Authors:** Linfeng Li, Jiajia Li, Li Yang, Jun Zhou, Yuying Ma

**Affiliations:** 1 College of Pharmacy, Chongqing Medical University, Chongqing 400016, China; 2 Basic Medicine Research and Innovation Center for Novel Target and Therapeutic Intervention (Ministry of Education), Chongqing Medical University, Chongqing 400016, China

**Keywords:** chemically induced proximity, molecular glue, undruggable, biomolecular complex

## Abstract

Chemically induced proximity (CIP) has revolutionized small-molecule pharmacology by moving beyond traditional occupancy-based inhibition and activation. Within this paradigm, monovalent nondegrading molecular glues (ndMGs) represent a structurally compact and mechanistically distinct class of linkerless compounds. These agents stabilize or induce selective biomolecular interactions, altering the functional state or cellular localization of macromolecular complexes without causing component degradation. Once considered rare phenomena unique to specific natural products, ndMGs are now being actively engineered across diverse therapeutic landscapes, with multiple candidates advancing into clinical trials and achieving regulatory validation. We systematically highlight their evolving capabilities to modulate oncogenic networks, intervene with immune activity, rectify metabolic signaling, control neurodegenerative trafficking and stress networks, and disrupt critical pathogen assemblies, while broadening the druggable landscape into non-canonical mechanisms. In each section, we analyze the therapeutic value of the targets, the molecular mechanisms of action, the latest progress, and the unique challenges faced by these strategies. In summary, the ndMG modality represents a highly versatile strategy to unlock the undruggable proteome.

## 1. Introduction

Traditional small-molecule pharmacology largely relies on occupying pre-existing pockets to inhibit or activate individual proteins. Although highly successful, this occupancy-driven paradigm is heavily biased toward proteins with deep, chemically tractable binding sites. Furthermore, it restricts therapeutic intervention mostly to simple inhibition or agonism. Chemically induced proximity (CIP) offers a complementary strategy by using small molecules to assemble, stabilize, or redirect biomolecular complexes [1]. Initially used as chemical biology tools, proximity-inducing modalities are now emerging into mainstream clinical development and therapeutic validation.

**Figure 1:**
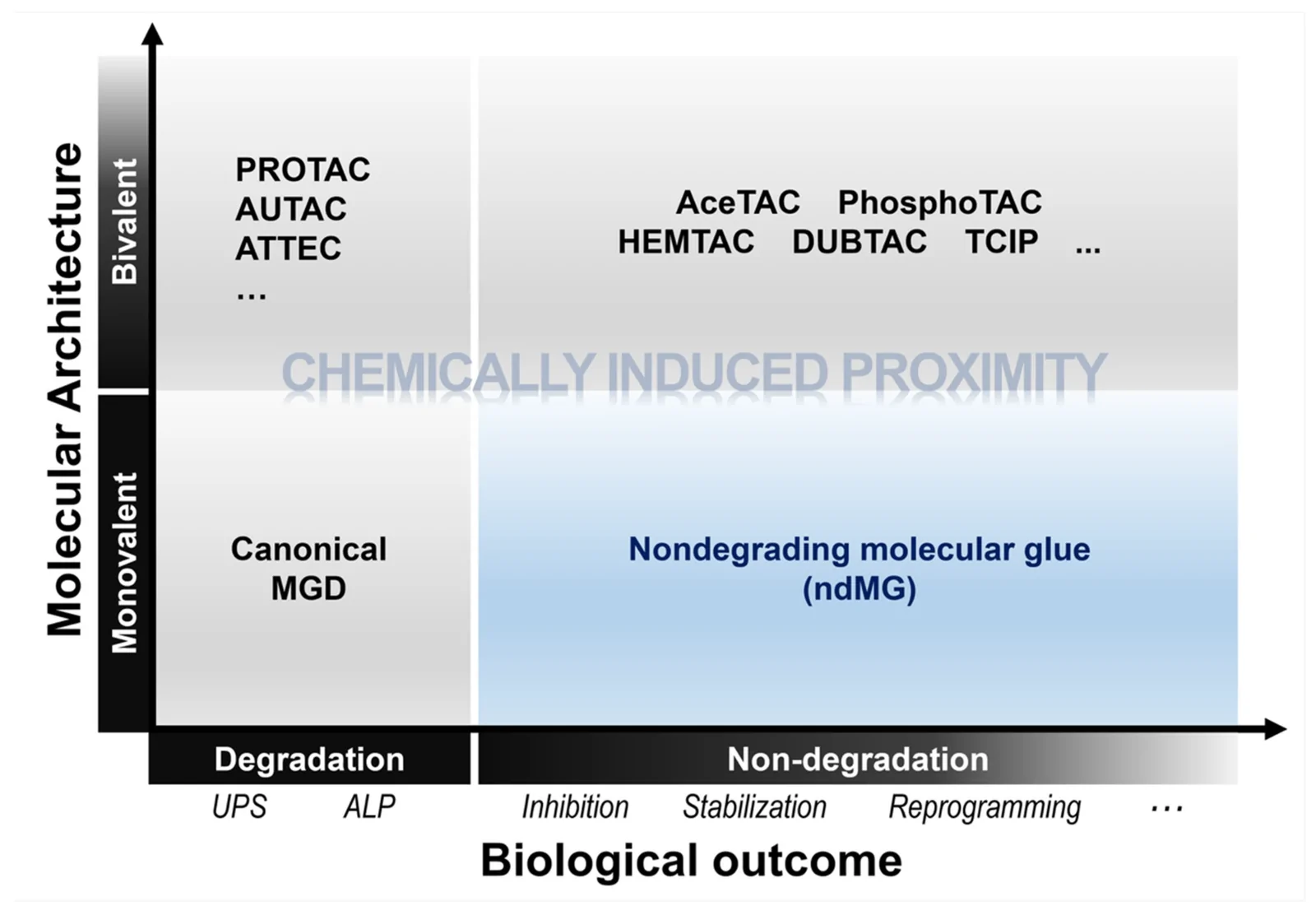
Classification of common chemically induced proximity modalities.

As summarized in Figure 1, the current landscape of CIP encompasses a broad range of modalities that differ in both molecular architecture and biological consequence. Monovalent molecular glue degraders (MGDs), such as thalidomide-based immunomodulatory drugs, typically recruit neosubstrates to E3 ligases for destruction [2]. In comparison, bifunctional technologies like PROTACs and ATTECs use a chemical linker to bridge target proteins with cellular degradation machinery [3]. Beyond degradation, bifunctional molecules can also achieve nondegradative outcomes by redirecting enzymes to alter target modification or transcription, as seen with AceTACs and DUBTACs [4,5]. Within this broader landscape, a monovalent nondegrading molecular glue (ndMG) is defined as a single, linkerless chemical entity that induces or stabilizes a selective biomolecular interaction, changing the function or localization of the resulting complex without destroying its components.

Unlike conventional ligands that require high-affinity engagement of a deep pocket, molecular glues derive their activity from cooperative stabilization of the entire protein interface. The compound often binds a presenter protein first, remodeling its surface to create a composite interface that recruits a second partner. This mechanism allows relatively shallow or flat protein surfaces to become pharmacologically accessible. Consequently, this mechanism can generate functional states difficult to achieve using traditional small molecules, including restoration of weakened regulatory complexes, enzymatic activation or silencing, selective sequestration, or altered trafficking. This cooperative recognition may also reduce the likelihood of drug resistance. Compared with bifunctional agents, the compact architecture of monovalent glues provides superior physicochemical properties, including lower molecular weight and better oral bioavailability. However, their discovery is less modular and harder to rationalize, as identifying these molecules requires screening strategies that evaluate the intact ternary complex rather than simple binary interaction.

The conceptual foundation of monovalent nondegrading glues stems from natural-product immunosuppressants [6]. Cyclosporin A and FK506 bind their respective immunophilin presenters to create a new surface that recruits and inhibits the phosphatase calcineurin. Similarly, rapamycin engages FKBP12 to recruit the FRB domain of mTOR, forming an inhibitory ternary complex. Expanding on this principle, Guo and colleagues developed the rapafucin platform in 2019 by constructing a library of 45,000 hybrid macrocycles that paired a conserved FKBP-binding domain with diversified peptide motifs [7]. This presenter-first approach led to the discovery of rapadocin, an FKBP-dependent inhibitor of the nucleoside transporter ENT1, demonstrating that engineered presenter modules can capture novel nondegradative interactions beyond natural targets. In parallel, a similar nondegradative proximity concept has achieved landmark translational success with RAS(ON) tri-complex inhibitors. A prominent example is the multi-selective agent daraxonrasib (RMC-6236), whose recent clinical success demonstrates that prospectively designed nondegrading molecular glues can effectively translate into late-stage therapeutic benefit [8].

Recent advances have propelled monovalent nondegrading molecular glues into major therapeutic areas, including oncology, immunology, metabolic diseases, neurological disorders, and infectious diseases. Rather than cataloging compounds strictly by presenter protein or structural class [9], this review organizes the emerging field by disease context and functional outcome, emphasizing target value, mechanistic evidence, clinical progress, and current translational challenges.

## 2. Nondegrading Molecular Glues for Cancer Therapy

Cancer has emerged as the leading therapeutic area for nondegrading molecular glues. This section examines how these compounds modulate major hallmarks of cancer and other tumor dependencies by remodeling or stabilizing biomolecular interactions (Table 1).

### 2.1. Suppressing Proliferative Signaling

Sustaining proliferative signaling is a defining hallmark of cancer, commonly driven by aberrant activation of kinase-centered pathways. A prototypical example of suppressing such signaling through induced proximity is rapamycin, which recruits FKBP12 to mTORC1 and allosterically inhibits its kinase output [6]. This classical ndMG established that small molecules can attenuate oncogenic signaling by remodeling regulatory protein complexes, a principle now extended to RAS and RAF/MEK signaling [10].

#### 2.1.1. RAS-Targeting ndMG

RAS proteins, encoded by KRAS, NRAS, and HRAS, are GDP/GTP-regulated small GTPases that transmit growth and survival signals through pathways such as RAF-MEK-ERK and PI3K-AKT-mTOR [11]. Oncogenic hotspot mutations impair GTP hydrolysis, locking RAS in a persistently active GTP-bound state and sustaining tumor-promoting signaling. Aberrant RAS signaling is implicated in approximately 20~30% of human cancers, with KRAS representing the dominant oncogenic isoform. Mutations occur mainly at codons G12, G13, and Q61, with distinct distributions across cancer lineages [12,13]. For decades, RAS was considered an “undruggable” oncoprotein, largely because its surface lacks deep ligandable pockets and its picomolar affinity for GDP/GTP makes nucleotide-competitive inhibition impractical [14,15]. This view changed with the development of first-generation KRAS inhibitors, represented by the FDA-approved agents sotorasib and adagrasib [16,17]. By exploiting the switch-II pocket and reacting with the mutant Cys12 residue, these agents lock KRAS G12C in its inactive GDP-bound “off” state [18]. However, their activity remains confined to a single allele and depends on cycling through the inactive RAS state.

These limitations motivated the development of RAS(ON) molecular glues that directly target active, GTP-bound RAS. Revolution Medicines pioneered this modality with macrocyclic compounds that first bind the abundant intracellular chaperone cyclophilin A (CypA), chemically remodeling its surface to create a new protein–protein interaction interface. The resulting CypA–drug binary complex then engages GTP-bound RAS to form an inhibitory ternary complex that sterically blocks effector binding, particularly RAS–RAF interaction [10,19]. Compared with first-generation KRAS G12C inhibitors, these monovalent, nondegrading RAS(ON) molecular glues offer several conceptual advantages. By targeting the active GTP-bound state rather than relying on cycling into the inactive GDP-bound state, they may better suppress tumors with high upstream nucleotide exchange activity and reduce adaptive pathway reactivation [20]. Multi-selective agents such as RMC-6236 also expand therapeutic coverage beyond KRAS G12C to common KRAS G12D, G12V, G12R, and related mutant contexts, which is especially relevant in pancreatic ductal adenocarcinoma [21,22]. In addition, simultaneous inhibition of oncogenic and compensatory wild-type RAS signaling may provide more complete pathway blockade. The modular tri-complex platform further enables both broad multi-selective inhibitors and mutant-selective agents, allowing therapeutic strategies to be tailored by genotype and tumor lineage.

Daraxonrasib, also known as RMC-6236, is the most clinically advanced representative of RAS(ON) molecular glues. It is an orally bioavailable, noncovalent, multi-selective RAS(ON) tri-complex inhibitor that recruits cyclophilin A (CypA) to active RAS, thereby sterically blocking effector engagement and suppressing downstream signaling [21]. Preclinical and translational studies demonstrated potent inhibition of MAPK pathway output, including pERK suppression, together with broad antiproliferative activity in RAS-addicted cancer models [22]. RMC-6236 showed the strongest activity in KRAS G12X-mutant contexts, while retaining activity in selected G13X, Q61X, NRAS, and HRAS models, and induced tumor regressions or marked tumor growth inhibition in NSCLC, pancreatic, and colorectal xenograft and patient-derived tumor models [22]. Clinically, daraxonrasib has advanced this modality from chemical proof of concept to late-stage therapeutic validation. Early-phase studies in previously treated RAS-mutant solid tumors showed antitumor activity in NSCLC and pancreatic ductal adenocarcinoma, with a manageable safety profile mainly characterized by rash and gastrointestinal adverse events [22]. Most notably, in the phase III RASolute 302 trial in previously treated metastatic PDAC, daraxonrasib met its primary endpoint, improving median overall survival versus chemotherapy from 6.7 to 13.2 months, with a hazard ratio of 0.40, while also prolonging progression-free survival without new safety signals [23]. Together with FDA breakthrough therapy and orphan drug designations in pancreatic cancer, these data position daraxonrasib as a leading late-stage clinical validation of RAS(ON)-directed, nondegrading molecular-glue inhibition.

Mutant-selective branches of the RMC platform are also advancing, including elironrasib/RMC-6291 for RAS G12C(ON), zoldonrasib/RMC-9805 for RAS G12D(ON), and RMC-5127 for RAS G12V(ON). Elironrasib (RMC-6291) is a G12C-selective RAS(ON) tri-complex inhibitor that, unlike first-generation KRAS G12C(OFF) inhibitors, targets active KRAS G12C and has shown clinical activity in KRAS G12C-mutant NSCLC after prior KRAS(OFF) inhibitor treatment, suggesting that ON-state blockade may retain activity in this resistance setting [24]. Zoldonrasib is an oral G12D-selective RAS(ON) tri-complex inhibitor that has generated encouraging phase 1 activity in previously treated KRAS G12D-mutant NSCLC and is being evaluated in combination regimens, including with daraxonrasib and standard therapies, in RAS G12D-driven cancers [25]. RMC-5127 extends this allele-selective strategy to RAS G12V; although publicly disclosed data remain primarily preclinical, the first-in-human phase 1/1b evaluation began in 2026 [26].

The clinical progress of Revolution Medicines’ programs has stimulated broader interest in RAS-targeting molecular glues beyond the original RMC chemical series. Among publicly disclosed non-RMC agents, ERAS-0015 from Erasca is an oral pan-RAS molecular glue in phase 1 development for RAS-mutant solid tumors, with preliminary antitumor activity reported and combination strategies under evaluation [27]. GFH276 from GenFleet is another clinical-stage pan-RAS(ON) molecular glue, supported by preclinical activity across RAS-driven models and now being evaluated in a phase 1 study [28,29]. Several additional preclinical candidates, including HEC228032 [30], SP09253 [31], RCZY-690 [32], HZ-V068 [33], HZ-V055 [34], and JAB-23E73 [35], have been disclosed mainly in recent AACR abstracts as orally active RAS(ON) molecular-glue inhibitors. Although still insufficiently disclosed, these programs highlight growing interest in expanding the chemical space of RAS-targeting molecular glues.

Not all emerging RAS glue concepts rely on CypA recruitment. A promising strategy is represented by RasGAP-gluing approaches, in which small molecules are proposed to stabilize or restore the native interaction between mutant RAS and endogenous GTPase-activating proteins [36], rather than recruiting CypA to block effector binding. A computational “glue docking” study identified candidate compounds predicted to reinforce the KRAS^G12D^–GAP interface with genotype-biased antiproliferative activity in vitro and in vivo [37]; however, no experimentally determined structure of a compound-stabilized RAS–GAP complex has yet been reported. More recently, an AACR abstract extended this concept to p120 RasGAP glues in NF1-null cells, aiming to compensate for neurofibromin loss and reduce RAS activity [38]. Although conceptually attractive because it seeks to restore endogenous RAS attenuation, RasGAP gluing remains an early-stage strategy with no publicly established clinical candidate.

Despite these advances, RAS-targeting monovalent nondegrading molecular glues still face important challenges. Acquired resistance has already been observed clinically, with emerging mechanisms including mutations that disrupt the drug-induced RAS-CypA interface or restore competitive RAS–RAF engagement, as well as downstream MAPK reactivation [39]. Encouragingly, the same study also explored mechanism-guided approaches to overcome these resistance mechanisms. RMC-4791 engages the KRAS switch-II region with reduced dependence on Y64, thereby retaining activity against Y64-mutant KRAS, whereas resistance driven by kinase-dead or hypoactive BRAF, which promotes RAF dimerization and strengthens RAS–RAF engagement, could be suppressed by combining daraxonrasib with agents that disrupt RAF dimerization or inhibit downstream MEK/ERK signaling. In addition, the therapeutic window of multi-selective RAS(ON) inhibitors requires continued evaluation because these agents can engage both mutant and wild-type RAS isoforms [40]. Clinically observed toxicities, particularly rash, gastrointestinal events, and stomatitis or mucositis, highlight the need for careful dose optimization, especially as these agents move into earlier-line and combination settings [41].

#### 2.1.2. RAF/MEK-Targeting ndMG

The RAF-MEK-ERK cascade is the canonical downstream effector pathway of RAS and a major proliferative signaling module in cancer. Activated RAS recruits RAF kinases, including ARAF, BRAF, and CRAF, which phosphorylate and activate MEK1/2, thereby driving ERK-dependent transcriptional programs that promote cell-cycle progression, survival, invasion, and therapy resistance [42]. Genetic activation of this axis occurs through diverse lesions, including BRAF V600 mutations, class II and class III BRAF alterations, KRAS and NRAS mutations, NF1 loss, and rare RAF or MEK rearrangements [42]. Although RAF and MEK have long been considered druggable nodes, conventional RAF or MEK inhibition is limited by pathway reactivation, genotype-dependent efficacy, and toxicity from sustained ERK suppression. Remodeling endogenous MAPK pathway protein–protein interfaces may offer an alternative strategy to address some of these limitations [43].

RAF–MEK clamps, which stabilize inactive RAF–MEK complexes, represent an important class of molecular glues that suppress downstream MAPK signaling. By trapping MEK in catalytically incompetent complexes with ARAF, BRAF, or CRAF, these agents suppress RAF-dependent MEK phosphorylation and may reduce rebound ERK activation in RAS- or RAF-driven tumors. Avutometinib, also known as VS-6766, CH5126766, or RO5126766, is the most advanced RAF–MEK clamp [44]. It inhibits MEK kinase activity while inducing dominant-negative RAF–MEK complexes that prevent MEK activation by RAF isoforms. In the phase 1 FRAME study, avutometinib plus the FAK inhibitor defactinib established an intermittent oral dosing schedule and showed proof-of-concept activity in MAPK pathway-driven tumors, particularly low-grade serous ovarian cancer, where the study reported an objective response rate of 42.3% and a median progression-free survival of 20.1 months, with higher activity in KRAS-mutant disease [45]. This intermittent schedule also suggested a potential tolerability advantage over continuous MEK inhibition. In May 2025, the FDA granted accelerated approval to avutometinib plus defactinib for adults with KRAS-mutated recurrent low-grade serous ovarian cancer after prior systemic therapy. In the supporting RAMP-201 study, the confirmed objective response rate was 44%, with duration of response ranging from 3.3 to 31.1 months [46,47].

Other RAF–MEK glues seek to improve the durability, breadth, and tissue distribution of MAPK pathway suppression. NST-628 is a nondegrading molecular glue that targets RAF–MEK signaling complexes across RAS- and RAF-driven tumor contexts [48]. Preclinical studies showed durable MAPK pathway inhibition and antitumor activity across diverse MAPK-altered cellular and patient-derived systems, with efficacy in intracranial xenografts supporting CNS penetration. This property is particularly relevant for MAPK-driven tumors involving the central nervous system, where limited brain penetration remains a challenge for many RAF- or MEK-directed therapies. NST-628 has entered phase 1 clinical evaluation in advanced solid tumors with RAS or RAF pathway alterations [49]. IK-595 represents another MEK–RAF molecular glue. It traps MEK in inactive complexes with RAF isoforms and blocks RAF-mediated MEK reactivation, leading to prolonged MAPK pathway inhibition in RAS/MAPK-altered cancer models [50]. Early clinical disclosures have reported target inhibition and preliminary antitumor activity [51,52], although its development path remains less established than that of avutometinib or NST-628.

Beyond direct RAF–MEK clamps, KSR–MEK interface stabilizers broaden this concept to scaffold-protein-mediated MAPK regulation. KSR proteins are pseudokinase scaffolds that organize RAF, MEK, and ERK signaling complexes. Although catalytically impaired, they regulate signaling by positioning MAPK components and facilitating RAF–MEK–ERK cascade propagation [53]. Structural studies of trametinib bound to KSR–MEK revealed that some MEK inhibitors do not simply bind isolated MEK; instead, their potency and residence time can be influenced by the KSR–MEK interface [54]. This work led to the design of “trametiglue,” a trametinib-derived tool compound engineered to enhance interfacial binding, stabilize KSR–MEK and RAF–MEK-related complexes, and limit adaptive resistance to MEK inhibition. Although trametiglue itself remains a chemical biology tool, it shows that classical MEK inhibitors can be redesigned to exploit scaffold-selective complex stabilization.

A complementary strategy shifts from RAF–MEK complex trapping to stabilization of autoinhibited RAF states. 14-3-3 proteins are dimeric phosphoserine/threonine-binding adaptors that regulate many signaling proteins, including RAF kinases [55]. In resting cells, 14-3-3 binding to phosphorylated CRAF, particularly at the S259 regulatory site, helps maintain CRAF in an inactive conformation and restrains inappropriate MAPK activation. Recent molecular-glue strategies aim to reinforce this native inhibitory interaction rather than block RAF catalytic activity directly. Konstantinidou et al. identified small-molecule stabilizers (such as compound **23**) of the 14-3-3σ-C-RAF autoinhibited complex, with lead compounds enhancing complex stability by up to approximately 300-fold in biophysical assays [56]. They also showed cellular 14-3-3/C-RAF engagement and partially blocked C-RAF dimerization and NRAS-C-RAF association. Virta et al. extended this principle to Noonan syndrome, using molecular glues to restore impaired 14-3-3/C-RAF regulatory interactions caused by RASopathy-associated C-RAF dysregulation [57].

### 2.2. Reactivating Cell Death Programs

Evading programmed cell death is a central hallmark of cancer and a major contributor to therapeutic resistance. Beyond conventional strategies that modulate apoptotic signaling, survival pathways, or cellular stress responses, nondegrading molecular glues provide a mechanistically distinct strategy to restore death-associated programs by stabilizing productive biomolecular complexes. This framework is now being leveraged for cell-death reactivation across enzymatic effectors, apoptotic regulators, and translational control nodes.

#### 2.2.1. PDE3A-SLFN12 ndMG

PDE3A–SLFN12 glues, commonly termed velcrins, illustrate a scaffold-enabled strategy for directly inducing tumor-cell death. PDE3A is a phosphodiesterase whose coexpression with the Schlafen-family protein SLFN12 defines a drug-sensitive state observed in selected tumor contexts, including melanoma, sarcoma, glioblastoma, ovarian cancer, and gastrointestinal stromal tumor [58]. Upon compound binding, PDE3A functions as a scaffold to recruit SLFN12, forming a complex that activates SLFN12-dependent RNA cleavage and disrupts translation, ultimately triggering a distinctive cytotoxic program described as SLFN12-dependent apoptosis or “tomoptosis”. Thus, activity is largely dictated by PDE3A/SLFN12 coexpression, making this class a biomarker-defined strategy rather than a broadly cytotoxic modality.

Several velcrins have progressed from chemical probes to translational candidates. DNMDP first established the PDE3A–SLFN12 gluing mechanism [59], whereas BRD9500 improved cellular potency and in vivo activity [60]. BAY 2666605 was subsequently optimized as an oral PDE3A–SLFN12 complex inducer with reduced PDE3A enzymatic inhibition and antitumor activity in PDE3A/SLFN12-positive xenograft and patient-derived models [61]. OPB-171775 has also been reported to induce PDE3A–SLFN12-dependent cytotoxicity, including in gastrointestinal stromal tumor models, further supporting the druggability of this interaction. However, clinical translation remains challenging [62]. In a first-in-human study of BAY 2666605 in biomarker-selected patients, only a small number of patients were treated, no objective responses were observed, and dose-limiting thrombocytopenia—likely due to PDE3A expression in platelets—prevented establishment of a therapeutic window [63]. These findings show that PDE3A–SLFN12 glues provide a clear proof of principle for death reactivation by induced complex formation, while also exposing pharmacokinetic and hematologic liabilities that remain to be solved.

#### 2.2.2. MCL1-Homodimerizing ndMG

Induced protein self-association provides a direct route to modulate the apoptotic machinery. MCL1 is a key anti-apoptotic member of the BCL-2 family that sequesters pro-apoptotic BH3-only proteins and BAX/BAK activators, thereby preventing mitochondrial outer membrane permeabilization [64]. Although multiple BH3-groove-directed MCL1 inhibitors have entered clinical development, no selective MCL1 inhibitor has yet been approved, and clinical progress has been constrained by safety concerns, including cardiac toxicity observed with some candidates [65]. A short macrocyclic peptide, 5L1, recently identified by RaPID screening, represents a molecular-glue-like alternative that binds MCL1 with nanomolar affinity [66]. Structural analysis showed that 5L1 can simultaneously engage two MCL1 molecules and induce MCL1 homodimerization, thereby distinguishing it from conventional BH3-groove occupancy by small-molecule inhibitors. Cellular antiproliferative and pro-apoptotic activity was demonstrated using the cell-penetrating derivative 5L1-TAT. Despite not being a conventional low-molecular-weight small molecule, the macrocyclic peptide 5L1 operates monovalently to stabilize a new protein–protein complex, offering a compelling proof of concept for disabling anti-apoptotic proteins through induced homodimerization.

### 2.3. Restoring Growth-Suppressive Functions

Loss or attenuation of growth-suppressive programs is another central feature of cancer [67]. In many tumors, these functions are not completely lost but instead weakened due to destabilized complexes, partially functional alleles, or disrupted regulatory interactions. Nondegrading molecular glues offer a pharmacological repair strategy in this setting by stabilizing weakened protein complexes, preserving active holoenzymes, or reconnecting tumor suppressors with protective regulators [6].

#### 2.3.1. SMAD4–SMAD3 ndMG

SMAD4 is the central common mediator of canonical TGF-β signaling, and loss of SMAD4 function disables the cytostatic arm of TGF-β responses in several epithelial cancers [68]. Importantly, some SMAD4 alterations do not simply eliminate the protein, but instead weaken productive signaling complexes. Hotspot mutations such as R361H and R361C are located at the SMAD3-interacting MH2 interface and impair SMAD3–SMAD4 complex formation, creating an opportunity for allele-selective molecular repair.

A proximity-based screening strategy identified Ro-31-8220, a bisindolylmaleimide derivative, as a small-molecule inducer of the SMAD4^R361H^–SMAD3 interaction [69]. Related bisindolylmaleimide analogs, including Go-6983, have also been reported to enhance SMAD4–SMAD3 complex formation in similar assay systems, suggesting that this chemotype can stabilize the mutant SMAD4 interface. In SMAD4-mutant cancer cells, these compounds reactivated dormant SMAD4^R361H^-dependent transcriptional activity and restored TGF-β-induced growth-suppressive responses. A covalent variant further extends this concept to SMAD4^R361C^. neoCMG101 exploits the mutation-created cysteine at the protein–protein interface, covalently modifies the neo-cysteine on SMAD4, strengthens the SMAD4^R361C^–SMAD3 interaction, and restores SMAD-dependent transcriptional output [70]. These studies have demonstrated that hypomorphic tumor suppressors can be repaired in an allele-selective manner and that mutant tumor suppressors may still be druggable when residual structure and pathway connections remain intact.

#### 2.3.2. PP2A-Stabilizing ndMG

PP2A provides another example in which tumor-suppressive activity depends on assembly of a functional protein complex. Rather than a single enzyme, PP2A comprises a family of heterotrimeric phosphatases formed by scaffold, catalytic, and regulatory B subunits [71]. The identity of the regulatory subunit determines substrate selectivity and pathway output, making PP2A activity highly dependent on holoenzyme stability. This architecture makes PP2A particularly suitable for pharmacological stabilization at interfacial pockets. DT-061, also known as SMAP-061 [72], was reported to stabilize the fully assembled B56α-containing PP2A holoenzyme by engaging an interfacial pocket formed across the scaffold, catalytic, and regulatory subunits. This biased stabilization promotes dephosphorylation of oncogenic substrates such as c-MYC and supports the concept that selective PP2A activation can be achieved without globally stimulating all PP2A complexes. However, a subsequent study failed to reproduce direct PP2A–B56 activation, instead linking DT-061 cytotoxicity to Golgi and ER homeostasis disruption, leaving its identity as a molecular glue disputed [73]. More recently, RPT04402 was described as a PP2A–B56α molecular glue that counteracts adaptive resistance to RAS/MAPK pathway inhibition in KRAS-mutant non-small-cell lung cancer. RAS/MAPK inhibitors can reduce PP2A catalytic-subunit carboxymethylation and destabilize B56α-containing heterotrimers; RPT04402 reverses this deconstruction, restores PP2A–B56α assembly, enhances apoptotic responses, delays resistance, and improves antitumor activity in combination models [74].

#### 2.3.3. p53 Pathway-Reactivating ndMG

As a master tumor suppressor frequently inactivated in cancer through mutation or dysregulated stability, p53 plays a central role in controlling cell-cycle arrest, apoptosis, and genomic integrity. Molecular glues that stabilize p53-associated regulatory complexes, therefore, provide a repair-oriented complementary strategy to conventional p53-directed approaches [75]. The manumycin-family polyketides asukamycin and manumycin A act as unusual molecular glues between TP53 and the E3 ubiquitin ligase UBR7. Rather than promoting p53 degradation, these electrophilic natural products facilitate formation of a UBR7–ligand–TP53 ternary complex, leading to p53 transcriptional activation and cancer-cell death. This mechanism highlights how components of the ubiquitin pathway can be repurposed to stabilize and activate p53 [76]. However, their electrophilic nature and limited translational validation remain important considerations for further development. A related but less well-characterized example is bromocriptine [77], which has been proposed as a putative p53–USP7 molecular glue. Bromocriptine enhances the p53–USP7 interaction and promotes USP7-mediated deubiquitination of p53, resulting in increased p53 stability and activation of downstream targets such as p21. Cellular studies show p53 accumulation and growth inhibition in relevant cancer models. Although further validation is needed, this example supports the broader principle that p53 function can be restored by stabilizing its interactions with regulatory partners.

### 2.4. Restricting Phenotypic Plasticity Through Transcriptional Rewiring

Beyond canonical oncogenic signaling and cell-death regulation, cancer cells are sustained by aberrant transcriptional states, chromatin dependencies, and non-mutational epigenetic reprogramming. These programs enable lineage plasticity, therapy adaptation, and persistent oncogenic output even in the absence of new genetic lesions [67]. However, many transcription factors and chromatin-regulatory assemblies lack conventional ligandable pockets, making them difficult to modulate with classical occupancy-driven inhibitors [78].

#### 2.4.1. BCL6–BRD9 ndMG

BCL6 is a key transcriptional repressor in germinal-center B cells and a major oncogenic driver in diffuse large B-cell lymphoma (DLBCL). Traditional approaches either disrupt BCL6–corepressor interactions or induce BCL6 degradation, as seen with BI-3802 and the clinical candidate LY4584180 [79,80]. These strategies validate BCL6 as a drug target but rely on inhibition or degradation rather than nondegradative rewiring. ZD-1-186 illustrates a distinct nondegradative strategy. This covalent molecular glue was derived from a BCL6-binding scaffold and incorporates an electrophilic fumaramide group that enables a monovalent ligand to engage BCL6 while capturing endogenous protein partners [81]. In DLBCL cells, ZD-1-186 suppresses MYC expression more effectively than representative BCL6 inhibitors or degraders, while also derepressing canonical BCL6 target genes such as CDKN1A/p21. Chemoproteomic and functional studies identified BRD9, a component of the noncanonical BAF chromatin-remodeling complex, as the key recruited partner. Covalent modification of BRD9 C288 stabilizes a BCL6–BRD9 complex, and BRD9 inhibition or depletion attenuates MYC repression and p21 induction. Thus, ZD-1-186 does not simply block BCL6 activity but redirects it toward a chromatin-regulatory partner to reshape transcriptional output.

#### 2.4.2. 14-3-3 ndMG for Transcription-Factor Sequestration

The same 14-3-3-centered stabilization logic can be extended from proliferative signaling to transcriptional control. In this context, 14-3-3 proteins serve as phospho-dependent regulatory platforms that trap or constrain transcription factors outside their fully active nuclear state. The best-developed example is ERα–14-3-3 stabilization in breast cancer [82]. ERα contains a C-terminal phosphosite, T594, that mediates 14-3-3 binding. The natural product fusicoccin A stabilizes this interaction, thereby reducing ERα dimerization, chromatin binding, and transcriptional activity, ultimately inhibiting the proliferation of ERα-positive breast cancer cells. This mechanism differs from ligand-binding-domain antagonism and may be relevant for endocrine-resistant settings in which conventional ER-directed therapies lose efficacy.

Subsequent medicinal chemistry efforts have produced more selective stabilizers of the 14-3-3σ–ERα interface. Covalent compounds, exemplified by compound **181**, that engage 14-3-3σ C38 enhance ERα complex stabilization with improved potency and selectivity, while optimized scaffolds have been supported by structural, biophysical, and cellular assays, including intracellular NanoBRET-based measurements [83,84]. Functionally, these glues suppress ERα-dependent transcription, inhibit growth in breast cancer models, including endocrine-resistant organoids, and may be compatible with existing therapies such as fulvestrant.

This principle may extend beyond ERα. EN171, a covalent ligand targeting 14-3-3σ cysteines, enhances 14-3-3 interactions with ERα, as well as the Hippo-pathway transcriptional coactivators YAP and TAZ [85]. By stabilizing these inhibitory complexes, EN171 reduces transcriptional activity in cells, suggesting a broader opportunity to restrain oncogenic transcriptional programs through 14-3-3-mediated sequestration. Nevertheless, these examples remain less mature than ERα-directed glues, with limited structural, in vivo, and disease-context validation.

### 2.5. Targeting Noncanonical Cancer Dependencies

Not all nondegrading glue mechanisms fall into established cancer hallmark categories. The following examples illustrate how they can modulate noncanonical cancer dependencies, such as adaptive stress responses and translational-control programs.

#### 2.5.1. Verteporfin as ndMG for IRE1α

Verteporfin, a clinically used photosensitizer, has recently been described as a molecular-glue-like activator of the ER stress sensor IRE1α [86]. Instead of inhibiting IRE1α kinase or RNase activity, verteporfin promotes IRE1α dimerization and activation, leading to autophosphorylation, XBP1 splicing, and downstream miR-153-mediated regulation of PTEN and BACH1. In breast cancer models, this enforced activation of IRE1α was reported to synergize with AKT inhibition, suggesting that an adaptive stress pathway can, under certain conditions, be pushed into a therapeutically unfavorable state for tumor cells. This interpretation should nevertheless be made cautiously, because IRE1α signaling can, in certain contexts, support malignant adaptation to proteotoxic stress and adverse microenvironmental conditions.

#### 2.5.2. 14-3-3–GIGYF2 ndMG

12-Deoxyfusicoccin (12-dFC), a semisynthetic fusicoccin derivative, further expands the 14-3-3-based gluing strategy from signaling and transcriptional regulation to post-transcriptional control [87,88]. 12-dFC stabilizes the interaction between 14-3-3 proteins and the intrinsically disordered translational repressor GIGYF2, particularly through a phosphorylated GIGYF2 motif. Stabilization of this complex enhances GIGYF2-dependent translational repression, reduces protein synthesis and glycolysis-related protein expression, and inhibits cancer-cell proliferation. This mechanism is consistent with the dependence of many tumors on high translational output and metabolic plasticity.

## 3. Nondegrading Molecular Glues for Immune and Inflammator Diseases

Immune and inflammatory responses are frequently governed by inducible multiprotein assemblies rather than by individual catalytic proteins. These complexes coordinate diverse events including innate immune activation, immune-cell recognition, inflammatory transcription and regulation.

### 3.1. Activating Innate Immune Signaling

**Figure 2:**
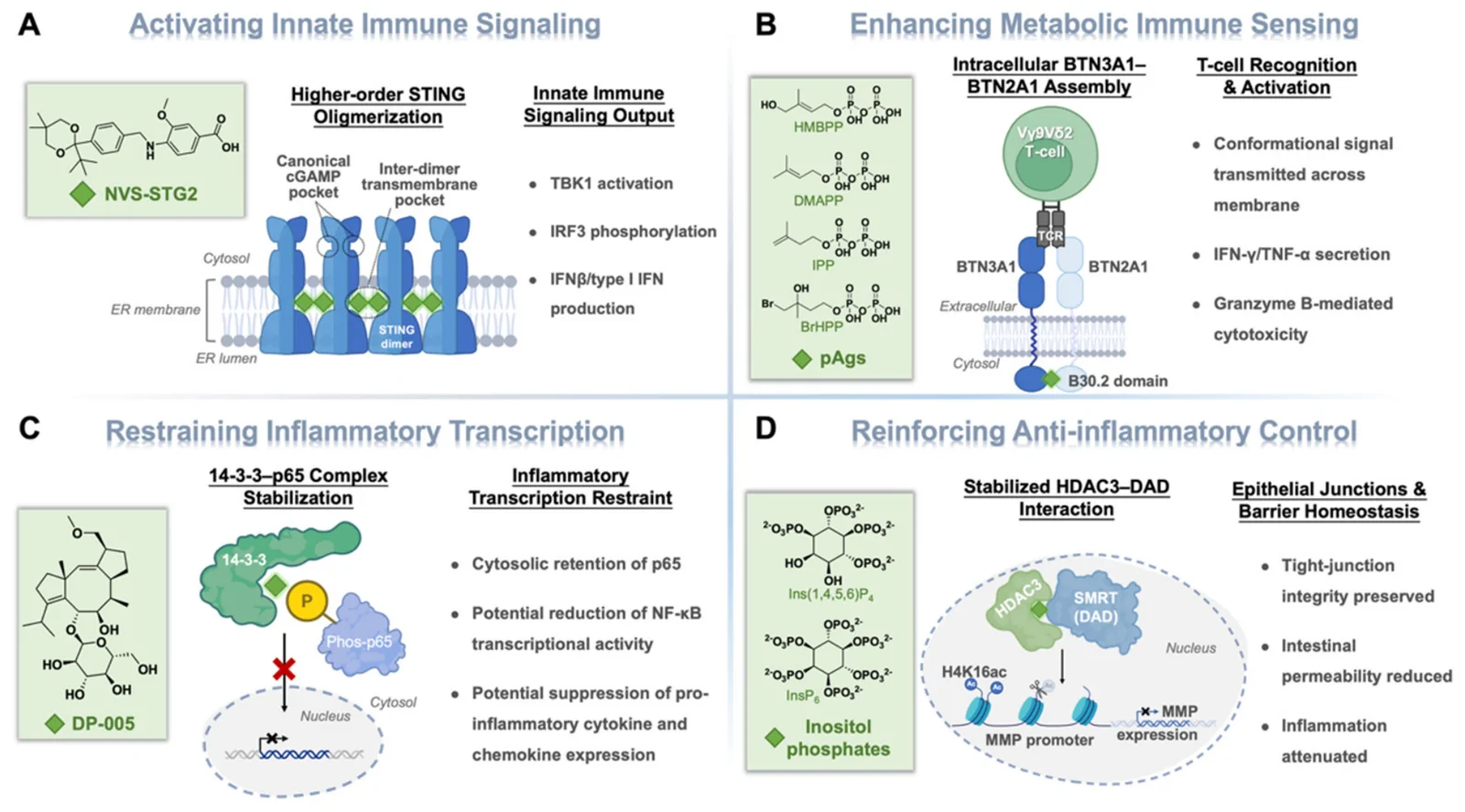
Chemical structures, mechanisms, and functional effects of ndMGs in immune and inflammatory regulation. (**A**) NVS-STG2 stabilizes STING oligomerization, activating TBK1-IRF3 signaling and type I interferon production. (**B**) Phosphoantigens promote BTN3A1-BTN2A1 assembly, triggering Vγ9Vδ2 T-cell recognition, cytokine secretion, and cytotoxicity. (**C**) DP-005 stabilizes the 14-3-3-p65 interaction, promoting cytosolic retention of phosphorylated p65 and potentially restraining NF-κB-dependent inflammatory transcription. (**D**) Inositol phosphates stabilize the HDAC3-DAD interaction, enhancing HDAC3 activity, suppressing MMP expression, and preserving intestinal epithelial barrier homeostasis.

As a central adaptor of cytosolic DNA sensing, STING is activated by cyclic dinucleotides such as cGAMP, triggering TBK1–IRF3 signaling and type I interferon production. While this pathway is attractive for cancer immunotherapy, antiviral responses, and vaccine adjuvants, conventional agonists are limited by delivery challenges and potential systemic inflammation [89]. NVS–STG2 represents a distinct mode of STING activation (Figure 2A) [90]. Rather than binding the canonical cyclic dinucleotide pocket, it engages an allosteric site in the transmembrane region. Structural studies show that two NVS-STG2 molecules occupy a cavity between adjacent STING dimers, stabilizing the dimer–dimer interface and promoting higher-order oligomerization. This architecture is consistent with a homomeric molecular glue mechanism. Mutations at residues such as R94, R95, and L134 selectively impair NVS–STG2 activity while largely preserving cGAMP responsiveness, supporting a mechanistically distinct glue pocket.

Cellular and in vivo studies further support this model. In engineered cells, NVS–STG2 drove STING oligomerization and puncta formation, accompanied by interferon-reporter activation. In human PBMCs, it induced IFNβ across major human STING haplotypes [90]. Because the compound is human-selective, antitumor activity was evaluated in human-STING knock-in mice: intratumoral treatment slowed MC38 tumor progression, with complete growth arrest in a subset of animals, and promoted systemic immune activation and tumor-specific T-cell priming in related models. Together, these findings establish molecular glue-induced STING oligomerization as a plausible preclinical strategy for immune activation, with potential applications in oncology, antiviral immunity, and vaccine adjuvancy.

### 3.2. Enhancing Metabolic Immune Sensing

Phosphoantigen sensing by human Vγ9Vδ2 T cells provides a natural example of metabolite-driven molecular glue in immune recognition. These T cells respond to phosphorylated metabolites such as HMBPP, DMAPP, and IPP, which are produced by microbes or accumulate during dysregulated isoprenoid metabolism in tumor cells [91]. Rather than acting as conventional extracellular receptor ligands, phosphoantigens bind the intracellular B30.2 domain of BTN3A1 and promote its association with BTN2A1 (Figure 2B). Structural studies show that HMBPP is positioned at the BTN3A1–BTN2A1 interface, creating a composite surface that stabilizes the butyrophilin heteromeric complex required for γδ T-cell activation [92].

Genome-wide CRISPR screening identified BTN3A1 and BTN2A1 as essential for HMBPP-dependent Vγ9Vδ2 T-cell killing. Biophysical assays further demonstrated phosphoantigen-dependent association of BTN3A1 and BTN2A1 intracellular domains, while mutagenesis of residues at the heteromeric interface impaired γδ T-cell activation [91]. Importantly, this mechanism is not limited to microbial HMBPP. Endogenous metabolites such as DMAPP and IPP, which arise from host isoprenoid metabolism and can accumulate after zoledronate treatment, also promote BTN3A1–BTN2A1 association, albeit with weaker activity. Together, these findings establish phosphoantigens as natural small-molecule glues that connect intracellular metabolic status to cell-surface immune surveillance. Prior phase I testing of another synthetic phosphoantigen bromohydrin pyrophosphate, also known as BrHPP or IPH1101, showed that its combination with low-dose IL-2 was generally well tolerated and induced robust expansion of Vγ9Vδ2 T cells [93], demonstrating the feasibility of phosphoantigen-based immune modulation in humans. IPH1101 was also explored in a small phase 2a study in chronic hepatitis C [94], further extending this immune-modulatory approach beyond oncology, although subsequent clinical development remained limited.

### 3.3. Restraining Inflammatory Transcription

NF-κB p65 is a central transcriptional driver of inflammatory and autoimmune responses, controlling cytokines, chemokines, survival factors, and immune effector genes. Because broad inhibition of upstream NF-κB signaling can cause excessive immunosuppression, reinforcing endogenous inhibitory interactions may offer a more selective therapeutic strategy [95]. Building on the same 14-3-3 interfacial-stabilization principle discussed in the previous section, DP-005 exemplifies this approach by reinforcing a potentially inhibitory 14-3-3–p65 interaction (Figure 2C).

Phosphorylated p65 contains 14-3-3-binding motifs, and its association with 14-3-3 has been linked to cytosolic retention and reduced nuclear activity. DP-005, a semisynthetic fusicoccane, stabilizes the 14-3-3–p65 complex by occupying an interfacial pocket formed between 14-3-3 and a phosphorylated p65 motif [95]. Structural studies showed that DP-005 contacts both binding partners and reorients the p65 phosphopeptide within the 14-3-3 groove, consistent with a heteromeric molecular glue mechanism. Quantitative binding studies further demonstrated strong cooperativity and selectivity for the p65/14-3-3 complex compared with other tested 14-3-3 interactions, suggesting that selectivity can arise from productive interfacial stabilization rather than simple ligand affinity.

Conceptually, this strategy could suppress NF-κB-dependent inflammatory gene expression by reinforcing p65 sequestration rather than globally blocking upstream receptor or kinase pathways. Such an approach may be relevant to chronic inflammatory diseases such as rheumatoid arthritis, inflammatory bowel disease, psoriasis, and other NF-κB-dependent disorders [96]. However, the current evidence remains mainly structural and biophysical, with no reported clinical studies or disease-model animal efficacy for DP-005. Therefore, it serves as an early tool compound rather than as an anti-inflammatory lead.

### 3.4. Reinforcing Anti-Inflammatory Control

Another example of metabolite-mediated molecular gluing occurs within HDAC3 corepressor complexes. HDAC3 is a class I histone deacetylase whose full catalytic activity depends on association with the transcriptional corepressors SMRT or NCoR through their deacetylase-activating domain (DAD). Structural studies identified Ins(1,4,5,6)P_4_ at this interface, where it contacts both HDAC3 and the corepressor to stabilize the active complex (Figure 2D) [97]. More recent work established the more abundant InsP_6_ as a physiological regulator of the same interaction. By strengthening DAD engagement with chromatin-bound HDAC3, InsP_6_ activates a preassembled but weakly active deacetylase complex [98].

The pathophysiological relevance of this mechanism has now been demonstrated in intestinal inflammation. Loss of IPMK, an upstream enzyme required for efficient cellular InsP_6_ synthesis, reduced epithelial HDAC3 activity and increased H4K16 acetylation at matrix metalloproteinase promoters [98]. The resulting MMP upregulation disrupted cell–cell junctions and increased intestinal permeability. Patient samples from Crohn’s disease and ulcerative colitis similarly showed reduced IPMK expression and HDAC3 activity, accompanied by elevated MMP expression. In intestine-specific IPMK-deficient mice and DSS-induced colitis models, oral InsP_6_ restored HDAC3 activity. This intervention suppressed MMP expression, improved epithelial barrier integrity, and attenuated intestinal inflammation.

## 4. Nondegrading Molecular Glues for Metabolic Signaling and Diseases

Metabolic diseases frequently involve dysregulated molecular assemblies that coordinate signaling, endocrine control, and metabolic adaptation. Although nondegrading molecular glues remain less clinically mature than those in oncology, several examples already illustrate how induced complex stabilization can be applied in this therapeutic area.

### 4.1. Amplifying Endogenous GLP-1R Signaling

**Figure 3:**
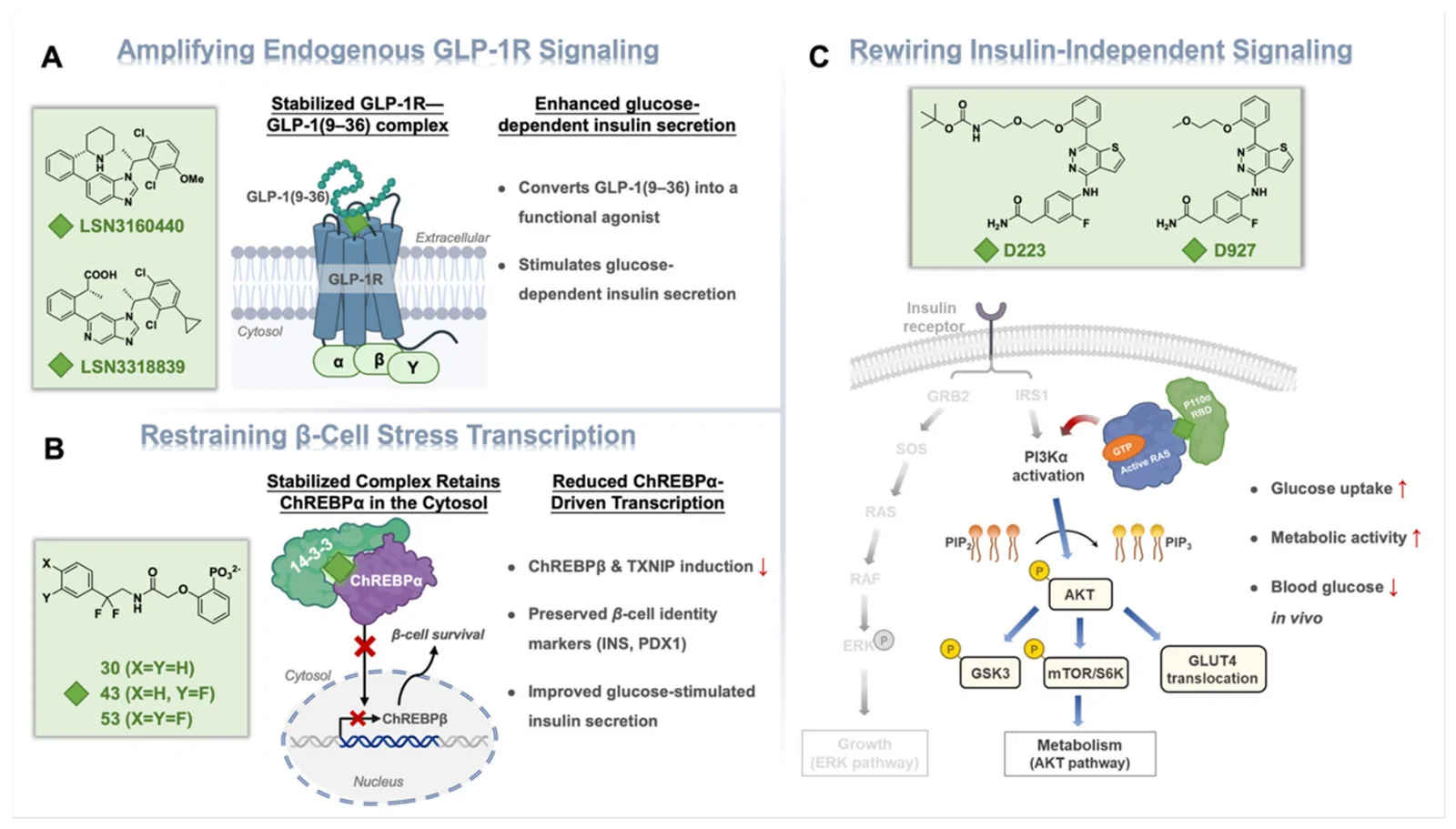
Chemical structures, mechanisms, and functional effects of ndMGs in metabolic signaling and diseases. (**A**) LSN3160440 and LSN3318839 stabilize the GLP-1R-GLP-1(9-36) complex, converting GLP-1(9–36) into a functional agonist and enhancing glucose-dependent insulin secretion. (**B**) Compounds **30**, **43**, and **53** stabilize cytosolic ChREBPα-14-3-3 association, reducing ChREBP-driven stress transcription and preserving β-cell function. (**C**) D223 and D927 stabilize the active RAS-PI3Kα interaction, activating AKT-dependent metabolic signaling and increasing glucose uptake independently of insulin receptor signaling.

Glucagon-like peptide-1 receptor (GLP-1R) signaling is a central axis in metabolic pharmacology. Current incretin therapies such as semaglutide largely rely on orthosteric peptide agonists that mimic or replace endogenous GLP-1 [99]. By contrast, small-molecule GLP-1R glues bind a transmembrane pocket and stabilize GLP-1R–peptide ligand complexes (Figure 3A) [100]. This mechanism is particularly relevant to GLP-1(9-36), a major GLP-1 metabolite with intrinsically weak agonist activity, because these compounds can convert its weak interaction with GLP-1R into an active ternary complex.

LSN3160440 was an early prototype of this class of GLP-1R glues [100]. Cryo-EM analysis showed that LSN3160440 occupies a composite pocket formed by GLP-1R and the bound peptide, directly reinforcing the receptor–peptide interface and, hence, stabilizing the complex in a Gs-coupled active state. Functionally, this compound displayed strong positive cooperativity with GLP-1(9–36), promoted glucose-, ligand-, and receptor-dependent insulin secretion in mouse islets, and showed insulinotropic activity during intravenous glucose challenge in rats. These data support its classification as a bona fide nondegrading molecular glue because the compound acts by stabilizing an endogenous ligand–receptor complex rather than directly replacing the ligand.

LSN3318839 extends this mechanism toward a more drug-like profile. This orally active GLP-1R positive allosteric modulator (PAM) converts GLP-1(9–36) from a weak metabolite into a functional agonist, while only modestly potentiating GLP-1(7–36) [101]. Structural studies suggest that GLP-1(9–36) alone adopts a relatively unproductive binding mode, whereas LSN3318839 reshapes the receptor–peptide complex toward a more active GLP-1(7–36)-like configuration [102]. Ex vivo, the compound enabled GLP-1(9–36)-dependent glucose-stimulated insulin secretion comparable to GLP-1(7–36), and in vivo, it lowered glucose under nutrient-stimulated conditions, including in combination with sitagliptin. Other GLP-1R PAM chemotypes, including compounds **3**–**20** and **3**–**24** identified from double-click triazole libraries, further support the generalizability of ligand-dependent receptor potentiation [103]. These compounds showed GLP-1(9–36)-dependent functional potentiation, although their proposed binding mode has not been structurally resolved and in vivo validation remains limited.

### 4.2. Restraining β-Cell Stress Transcription

As shown by several 14-3-3-based glues discussed above, the same adaptor–scaffold logic can be repurposed by stabilizing distinct 14-3-3 client complexes. In β cells, this strategy can be directed toward transcriptional restraint through the ChREBPα-14-3-3 axis [104]. Under basal conditions, 14-3-3 binding helps retain ChREBPα in the cytoplasm. During chronic hyperglycemia or glucolipotoxic stress, ChREBPα enters the nucleus and promotes a feed-forward transcriptional program involving ChREBPβ, TXNIP, and other targets associated with β-cell dysfunction and death. Rather than inhibiting ChREBP DNA binding directly, molecular glues can reinforce the cytoplasmic ChREBPα-14-3-3 complex and thereby limit stress-induced transcriptional activation.

Recent fluorinated analogs, including compounds **30**, **43**, and **53**, provide the most advanced chemical-biology evidence for this mechanism (Figure 3B) [104]. These compounds stabilize the ChREBPα-14-3-3 interaction with low-micromolar activity and substantial cooperativity. X-ray structures show that compounds **30** and **43** occupy a pocket at the protein–protein interface and bridge the 14-3-3/client complex, with compound **43** benefiting from a helix-9 clamping effect that rationalizes its improved activity. Importantly, the series shows selectivity for ChREBPα over other tested 14-3-3 client peptides, and compound **43** retains activity across multiple 14-3-3 isoforms.

The cellular pharmacology is also unusually coherent for a transcription-factor glue. In INS-1 *β*-like cells and primary human islets, active compounds increased ChREBPα-14-3-3 proximity, blocked glucose- or glucose-plus-palmitate-induced ChREBP*α* nuclear translocation, suppressed ChREBP*β* and TXNIP induction, preserved *β*-cell identity markers such as INS and PDX1, and improved glucose-stimulated insulin secretion under glucotoxic conditions [104]. These effects connect ternary-complex stabilization to transcriptional correction and a disease-relevant *β*-cell phenotype. Despite these promising data, public reports have not yet disclosed rodent PK/PD, oral exposure, or in vivo diabetes efficacy for compounds **30**, **43**, or **53**. In addition, compound **43** also attenuated glucose-stimulated proliferation in INS-1 cells, raising a potential, but as yet unproven, concern that chronic ChREBP restraint could limit adaptive *β*-cell expansion in vivo.

### 4.3. Rewiring Insulin-Independent Signaling

The RAS–PI3K*α* interface is usually discussed in cancer biology, but it also provides a striking example of metabolic signaling rewiring [105]. D223 and D927 were identified in the search for insulin-independent glucose uptake and glucose lowering (Figure 3C) [106]. Mechanistically, these compounds bind primarily to the RAS-binding domain of p110*α*, stabilize it in an RAS-compatible conformation, and make interfacial contacts with active RAS. Therefore, they enhance the active RAS–p110*α* interaction without simply increasing RAS GTP loading or broadly strengthening all RAS effector interactions; notably, D927 did not enhance RAS binding to CRAF.

The structural evidence is particularly strong. Crystal structures of p110α–RBD with D223 and of KRAS–p110α complexes with D223 or D927 show how the compounds create or stabilize an induced pocket in p110α while also engaging residues on KRAS [106]. Biochemically, D223 and D927 enhance the otherwise weak active KRAS–p110α interaction by nearly three orders of magnitude, providing a molecular basis for their downstream signaling effects. In vivo studies extended this insulin-mimetic activity to multiple rodent settings. D927 rapidly lowered blood glucose and improved glucose metabolism in normal and Zucker fatty rats, while also correcting hyperglycemia in models of both type 1 and type 2 diabetes, including insulin-deficient settings. Unlike GLP-1R potentiators, D223 and D927 create a neomorphic signaling bypass that couples active RAS to PI3Kα, thereby activating the PI3K–AKT metabolic axis independently of the canonical insulin receptor–IRS pathway. Nevertheless, the translational potential of this approach remains uncertain, particularly for chronic metabolic therapy. Although D927 did not appreciably activate the RAF–MEK–ERK pathway, sustained enhancement of PI3Kα–AKT signaling may itself carry growth- and survival-related liabilities, and the current in vivo studies extend only over several weeks. Thus, the long-term safety of sustained RAS–PI3Kα gluing remains to be established.

## 5. Nondegrading Molecular Glues for Neurological Disorders

Neurological disorders have already provided a classical precedent for glue-based pharmacology. Everolimus, a rapalog that acts through FKBP12-mediated inhibition of mTORC1, is approved as an adjunctive treatment for tuberous sclerosis complex (TSC)-associated partial-onset seizures [107], illustrating that stabilizing or reconfiguring regulatory protein complexes can produce clinically meaningful effects in neurological disease. Recent work has expanded this approach to neurological processes shaped by stress-response control, ion-channel gating, organelle-contact remodeling, and endosomal trafficking.

### 5.1. Restoring Stress-Impaired Translation

**Figure 4:**
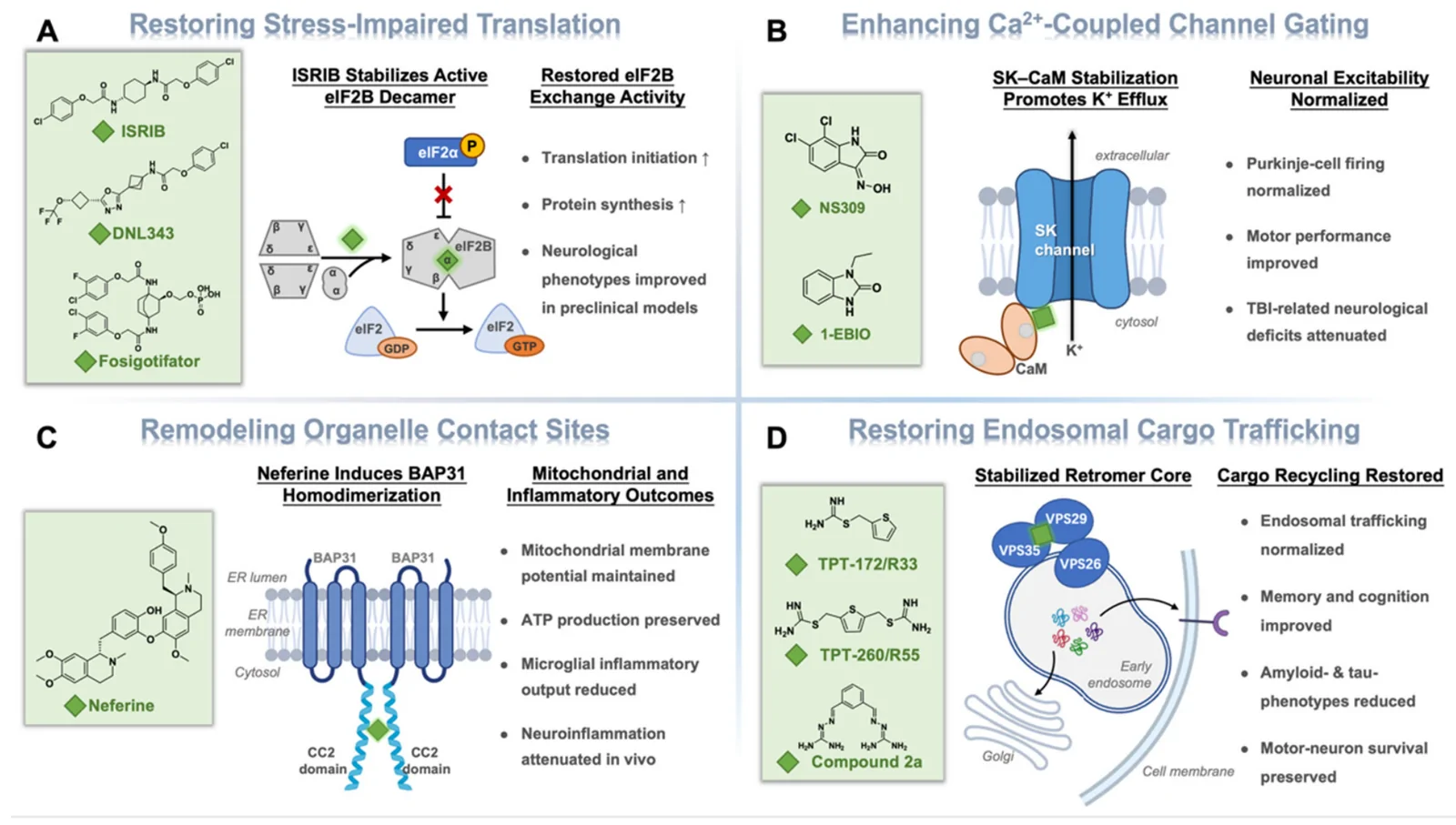
Chemical structures, mechanisms, and functional effects of ndMGs in neurological disorders. (**A**) ISRIB and related eIF2B activators stabilize the active eIF2B decamer, restore guanine-nucleotide exchange activity, and promote translation under stress conditions. (**B**) K-channel modulators such as NS309 and 1-EBIO stabilize CaM-coupled channel gating, enhance K^+^ efflux, and help normalize neuronal excitability. (**C**) Neferine induces BAP31 homodimerization, thereby preserving mitochondrial function and attenuating inflammatory responses. (**D**) Retromer stabilizers reinforce the VPS35-VPS29-VPS26 core, restore endosomal cargo trafficking, and improve neurodegeneration-related phenotypes.

eIF2B is the central guanine-nucleotide-exchange factor that reactivates eIF2 during translation initiation, making it a key control point of the integrated stress response. ISRIB, named for its ability to inhibit the integrated stress response, is the prototype small-molecule eIF2B activator in this class (Figure 4A) [108]. Structurally, ISRIB binds a deep, symmetric cleft at the eIF2B decamer interface, bridging the two halves of the guanine-nucleotide-exchange factor and stabilizing its active decameric assembly [109]. Later mechanistic work further showed that ISRIB does not merely stabilize eIF2B in a static manner, but also allosterically counteracts the inhibitory conformation imposed by phosphorylated eIF2α [110]. This mechanism captures a form of nondegrading glue logic that is especially relevant to neurology, as chronic integrated stress response activation is observed in traumatic injury, leukodystrophy, TDP-43 proteinopathy, and multiple proteostasis-related disorders [111,112,113]. In such conditions, restoring the function of a stressed translation-control complex provides a rationale for testing eIF2B activation across neurological settings marked by persistent ISR signaling.

Preclinical evidence supports this rationale across several neurological models. ISRIB and related eIF2B activators reversed persistent cognitive deficits after traumatic brain injury, restored memory and synaptic phenotypes in Down syndrome models, delayed neurodegeneration in prion disease, and improved age-related cognitive decline [111,114,115,116]. More recently, CNS-penetrant eIF2B activators reduced ISR biomarkers and modestly delayed disease features in TDP-43 and vanishing-white-matter-related models [116]. Despite this breadth of preclinical evidence, clinical translation has been more sobering. DNL343 showed substantial CSF exposure, ISR biomarker modulation, and generally acceptable tolerability in healthy volunteers and participants with ALS, but did not meet the primary efficacy endpoint in the HEALEY ALS platform trial [113,117]. Fosigotifator, another investigational eIF2B activator, has advanced into phase 1b/2 testing in vanishing white matter disease, where the mechanism–genotype match is tighter than that in ALS [118]. Regulatory support has also strengthened, with FDA Breakthrough Therapy Designation in June 2026. Together, these outcomes suggest that the therapeutic value of eIF2B activation may depend strongly on disease context, timing, and biomarker-guided patient selection.

### 5.2. Enhancing Calcium-Coupled Channel Gating

Small-conductance Ca^2+^-activated potassium channels, also known as SK or KCa2 channels, are key regulators of neuronal excitability. Through constitutively associated calmodulin (CaM), these channels couple intracellular Ca^2+^ signals to K^+^ efflux, thereby controlling afterhyperpolarization, firing frequency, and network activity [119]. Dysregulated KCa2-channel activity is implicated in cerebellar excitability disorders such as tremor and ataxia, while pharmacological KCa-channel activation has also shown protective effects in experimental traumatic brain injury.

Several reported compounds function as positive modulators of the SK–CaM interface (Figure 4B). Although these agents are not always described as molecular glues in studies of SK-channel pharmacology, structural evidence supports a glue-like mechanism. In this mechanism, early modulators such as 1-EBIO occupy pockets at the CaM–channel interface and strengthen the coupling between Ca^2+^ sensing and pore opening [120]. Recent cryo-EM studies further showed how NS309, a more potent tool compound, engages active-state pockets and alters channel gating [121]. By enhancing SK-channel gating through this interface mechanism, these compounds have shown activity in models of cerebellar and network dysfunction. In SCA3 mice, SKA-31 partially corrected abnormal Purkinje-cell firing and improved motor performance, while in experimental traumatic brain injury, NS309 reduced edema, inflammation, apoptosis, and neurological deficit scores [122,123]. Clinical translation has also moved beyond proof of concept. The KCa2 activator CAD-1883, also known as rimtuzalcap, reached phase 2 testing in essential tremor and spinocerebellar ataxia, suggesting that oral exposure and tolerability were sufficient to justify human studies [124]. Nevertheless, peer-reviewed neurological efficacy data and detailed human CNS pharmacokinetic information remain limited or publicly unspecified.

### 5.3. Remodeling Organelle Contact Sites

A conceptually novel case is neferine-induced BAP31 homodimerization. In a recent study, neferine was identified by thermal proteome profiling as a BAP31 ligand and was shown to bind the CC2 coiled-coil region, thereby inducing stable BAP31 homodimerization (Figure 4C) [125]. This dimerized state remodeled ER membrane architecture and disrupted ER–mitochondria contact sites. As a result, mitochondrial energetic homeostasis was preserved and microglial inflammatory output was reduced. This finding is notable because it moves the concept beyond soluble protein complexes and into the regulation of an ER-resident membrane protein. It also suggests that neuroinflammation can be modulated not only through receptor antagonism, but also by altering organelle-contact topology. The study also reported anti-inflammatory activity in LPS endotoxemia and neuroprotection in MCAO ischemic stroke, linking the molecular event of BAP31 dimerization to disease-associated phenotypes in vivo. The liabilities are equally clear. Neferine remains an early natural-product lead; brain exposure and medicinal-chemistry tractability have not yet been clearly defined. Furthermore, the safety consequences of prolonged manipulation of ER–mitochondria contacts remain unknown.

### 5.4. Restoring Endosomal Cargo Trafficking

The retromer is an endosomal sorting complex that retrieves selected cargo from endosomes and redirects it to the trans-Golgi network or plasma membrane, thereby supporting neuronal trafficking homeostasis. Small-molecule retromer stabilizers reinforce the VPS35–VPS29–VPS26 core and have emerged as a representative class of trafficking-complex glues in neurodegenerative disease (Figure 4D). TPT-260/R55 was identified through structure-based screening for compounds predicted to occupy a hotspot near the VPS35–VPS29 interface and was subsequently shown to stabilize the retromer complex [126]. In SORL1-deficient or SORL1-variant human neurons, TPT-260/R55 corrected endosomal enlargement and trafficking abnormalities while improving amyloid- and tau-related phenotypes [127]. The related stabilizer TPT-172/R33 has also reduced Aβ and phosphorylated tau in other cellular and animal models of Alzheimer’s disease [128]. Compound **2a** further broadens this platform beyond Alzheimer’s-spectrum models [129]. NMR studies confirmed that compound **2a** binds the VPS35–VPS29 heterodimer. The compound was able to cross the blood–brain barrier and ameliorate locomotor decline while preserving motor-neuron survival in SOD1-G93A ALS mice. Collectively, these data make retromer stabilization an unusually attractive platform for neurological disease, because it directly targets a disease-linked trafficking complex rather than a single receptor, enzyme, or aggregate species. Its translation, however, will depend on solving two familiar problems for CNS molecular glues: how to measure target engagement in the human brain, and how to identify disease subtypes that are sufficiently retromer-dependent to yield a clinical signal.

## 6. Nondegrading Molecular Glues for Infectious Diseases

Infectious diseases involve extensive protein-interaction networks within pathogens and between pathogens and their hosts. Viral replication, particle maturation, immune evasion, and bacterial envelope biogenesis often depend on dynamic or ordered macromolecular assemblies, making these processes particularly amenable to modulation by molecular glues.

### 6.1. Disrupting Virion Maturation

**Figure 5:**
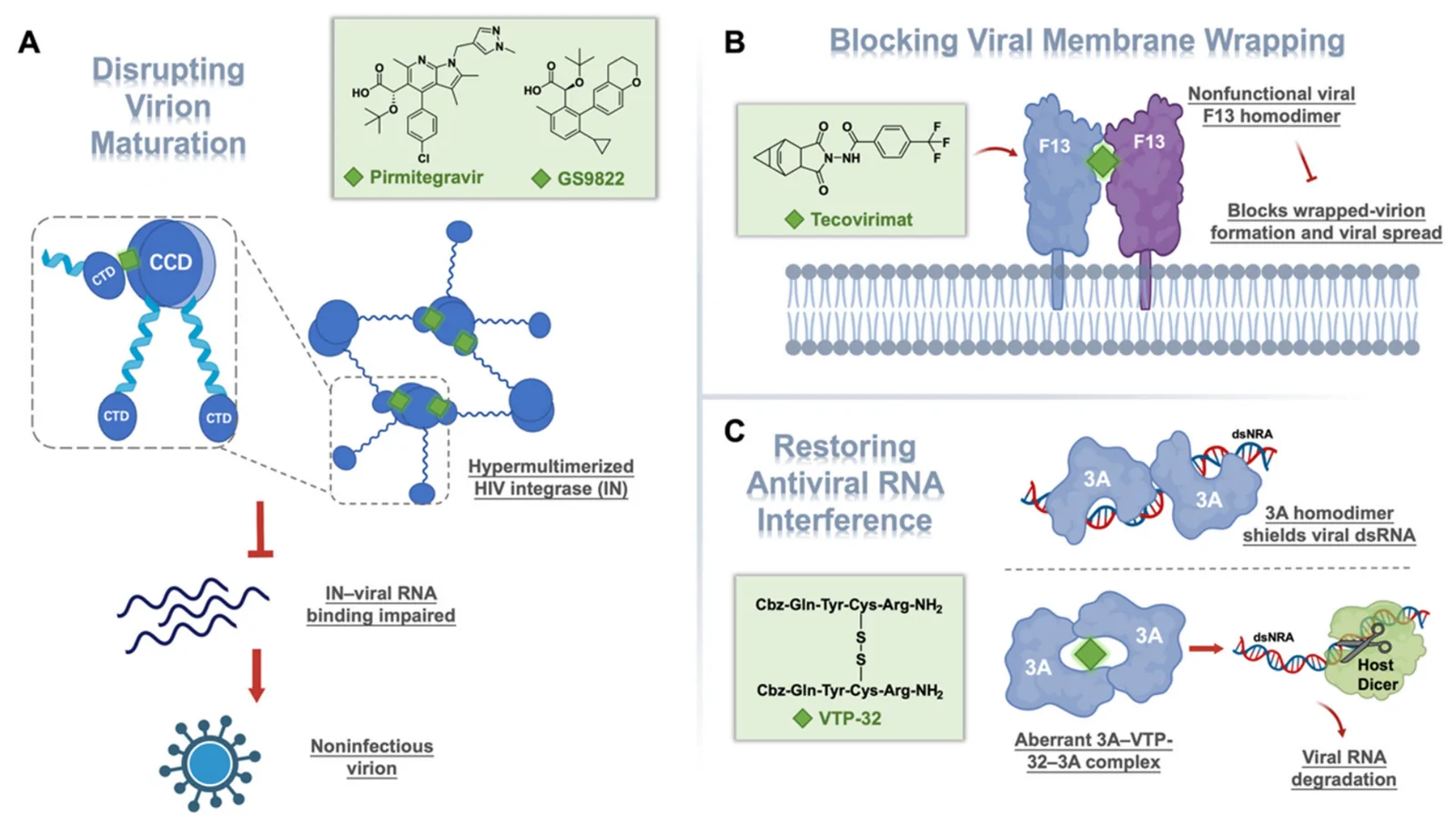
Chemical structures, mechanisms, and functional effects of ndMGs in infectious diseases. (**A**) ALLINIs such as pirmitegravir and GS-9822 stabilize aberrant integrase multimers, impair integrase-viral RNA binding, and disrupt virion maturation to produce noninfectious particles. (**B**) Tecovirimat stabilizes a nonfunctional F13 homodimer, blocking wrapped-virion formation and viral spread. (**C**) VTP-32 induces an aberrant 3A complex, exposing viral dsRNA to Dicer and restoring RNAi-mediated viral RNA degradation.

HIV-1 integrase (IN) is a clinically validated antiviral target, but approved integrase strand transfer inhibitors act at the catalytic site and can share overlapping resistance profiles. Allosteric integrase inhibitors, also known as ALLINIs, LEDGINs, or INLAIs, represent a mechanistically distinct class of nondegrading molecular glues (Figure 5A) [130,131,132]. These compounds bind the conserved LEDGF/p75-binding pocket at the dimer interface of the integrase catalytic core domain. Rather than merely disrupting cofactor binding, potent ALLINIs extend from this pocket and create an ectopic interface between the catalytic core domain and the C-terminal domain of another integrase molecule. Structural studies of BI-D, pirmitegravir/STP0404, and BDM-2 showed that these compounds engage conserved residues such as Tyr226, Trp235, and Lys266, thereby driving aberrant integrase hypermultimerization [131,132]. An essential viral assembly factor is consequently trapped in aberrant higher-order multimers.

This mechanism produces antiviral effects at two stages of the viral life cycle. During early infection, occupation of the LEDGF/p75 pocket can impair productive integration and redirect residual proviral integration away from transcriptionally active chromatin. This property has been explored in “block-and-lock” latency strategies using compounds such as CX14442 and GS-9822 [133]. The more pronounced antiviral effect, however, occurs during late replication, when ALLINIs disrupt virion morphogenesis. Drug-induced integrase hypermultimerization interferes with integrase binding to viral RNA and produces eccentric, noninfectious virions [134]. These defective particles subsequently fail to support efficient reverse transcription in newly infected cells.

Pirmitegravir/STP0404 has completed phase 2a proof-of-concept testing, and preliminary clinical data demonstrated short-term antiviral activity in individuals with HIV-1 [135,136]. In addition, BDM-2 has completed a single-ascending-dose phase 1 study in healthy volunteers [137], while separate in vitro profiling demonstrated that the compound retained full activity against HIV-1 variants resistant to INSTIs and multiple other antiretroviral classes [132]. Further development will require improved pharmacological properties and careful management of resistance mutations arising at the induced protein–protein interface [138]. Compound-specific liabilities also remain relevant, as illustrated by the species-specific toxicity reported for GS-9822 [139].

### 6.2. Blocking Viral Membrane Wrapping

Orthopoxviruses, including variola virus and monkeypox virus, encode the conserved F13 protein, a membrane-associated phospholipase-like factor required for wrapped virion formation. F13 supports virion envelopment, viral egress, and cell-to-cell spread, making it a clinically validated target for restricting orthopoxvirus dissemination. Tecovirimat, an approved treatment for smallpox that has also been used during mpox outbreaks, acts by trapping F13 in an aberrant oligomeric state (Figure 5B) [140,141].

Structural studies showed that tecovirimat binds within a conserved cavity formed between two F13 protomers [140]. F13 is predominantly monomeric in solution, whereas drug binding promotes the formation of a stabilized dimer. Tecovirimat, therefore, does not simply occupy an inhibitory pocket on an individual F13 molecule. Instead, it creates and reinforces a protein–protein interface that traps F13 in a nonfunctional homodimeric state.

This drug-stabilized assembly interferes with viral membrane wrapping and limits the production of extracellular virions, thereby restricting viral spread. The resistance profile of tecovirimat further supports this molecular-glue mechanism. Clinically observed resistance substitutions cluster around the induced dimer interface and weaken drug-dependent F13 association [142], directly linking disruption of the stabilized complex to loss of antiviral activity.

### 6.3. Restoring Antiviral RNA Interference

Enteroviruses, including EV-A71, coxsackieviruses, echoviruses, and EV-D68, cause a broad spectrum of diseases ranging from hand-foot-and-mouth disease and respiratory infection to myocarditis and severe neurological complications. These pathogens all encode the nonstructural protein 3A, which contributes to viral replication and immune evasion. The 3A protein also acts as a viral suppressor of RNA interference (Figure 5C) [143]. Its homodimeric form shields double-stranded RNA replication intermediates from Dicer-mediated processing, thereby limiting the production of antiviral small interfering RNAs and preserving viral genomes.

VTP-32 was developed to disrupt this protective function by remodeling the oligomeric state of 3A [144]. The compound promotes formation of an aberrant 3A–VTP-32–3A complex in which 3A can no longer efficiently shield viral double-stranded RNA. The exposed RNA intermediates become accessible to Dicer, leading to the generation of virus-derived small interfering RNAs and subsequent RNAi-mediated degradation of viral genomes.

VTP-32 showed antiviral activity against several enteroviruses, including EV-A71, CV-A10, CV-A16, echovirus 11, and EV-D68. In an EV-A71 neonatal mouse model, treatment improved survival and reduced clinical disease severity [144]. Viral burdens were also lowered in multiple affected tissues, including the brain, muscle, lung, and heart. Further development will require improvement of its peptidomimetic architecture, delivery properties, and pharmacokinetic profile.

## 7. Emerging Mechanistic Frontiers and New Targets

### 7.1. GID4–ACAA1 Interaction for Allosteric Enzyme Inhibition

Chetan et al. demonstrated that a small peptidomimetic binds GID4 and allosterically remodels its surface to promote association with the peroxisomal thiolase ACAA1 [145]. Unlike canonical interfacial glues, it contacts only GID4, with newly enabled protein–protein interactions driving ternary-complex formation. Recruitment does not result in ACAA1 ubiquitination or degradation but locks the enzyme in an inactive-like state and suppresses its thiolase activity. ACAA1 inhibition may have therapeutic potential in selected metabolically dependent tumors. Genetic or pharmacological suppression of ACAA1 restrains proliferation and enhances CDK4/6 inhibition in ACAA1-high triple-negative breast cancer [146], while ACAA1 loss reduces energy production and tumor growth in pancreatic cancer models [147]. ACAA1 has also been linked to ferroptosis resistance in endometrial cancer [148]. Importantly, these disease associations derive from ACAA1 target-validation studies rather than from CLEO4-88 itself. Whether CLEO4-88-mediated catalytic trapping reproduces these cellular and antitumor phenotypes remains untested. Moreover, ACAA1 loss can be detrimental in other tissues and tumor types, emphasizing the need for context-specific biomarkers and targeted delivery.

### 7.2. FKBP12-Binding Molecular Glues Beyond Macrocycles

An FKBP12-biased DNA-encoded library screen identified non-macrocyclic compounds that recruit FKBP12 to either the BRD9 bromodomain or quinoid dihydropteridine reductase (QDPR) [149]. Structural studies showed that the compounds occupy the acyl-lysine-recognition pocket of BRD9 or part of the NADH-binding pocket of QDPR, while FKBP12 supplies additional protein contacts. One QDPR glue displayed no measurable target binding without FKBP12, demonstrating strong presenter dependence. The work establishes a prospective route to FKBP12 glues beyond rapamycin-like macrocycles. Nevertheless, the reported compounds remain low-micromolar discovery hits, and the study demonstrated recruitment rather than functional modulation.

### 7.3. Covalent Modification of Phosphatases for Target Binding

DML189 illustrates a less conventional form of nondegradative protein gluing [150]. The electrophilic compound covalently modifies SHP2 at Y279 near the catalytic pocket and promotes reversible disulfide-mediated tethering with other proteins, leading to depletion of monomeric SHP2. Crosslinking mass spectrometry implicated several regulatory cysteines in the resulting assemblies, whereas related phosphatases underwent less extensive tethering. This mechanism suggests that phosphatases may be inhibited through ligand-induced protein association involving residues outside the highly conserved catalytic cysteine. However, DML189 does not generate a single structurally defined ternary complex, and the recruited partners and assembly stoichiometries appear heterogeneous.

### 7.4. GPCR Homodimerization for Sustained G-Protein-Biased Signaling

AP-7-168 is a membrane-protein molecular glue that modulates β2AR signaling through receptor homodimerization [151]. Two AP-7-168 molecules pack within a composite transmembrane pocket formed by TM3, TM4, and TM5 of two β2-adrenergic receptor protomers, thereby stabilizing a defined β2AR homodimer. The resulting assembly sterically restricts GRK-mediated phosphorylation and β-arrestin core engagement while retaining substantial Gs coupling and cAMP signaling. AP-7-168, therefore, shifts β2AR signaling toward a sustained G-protein-biased state and may delay receptor desensitization, consistent with the prolonged bronchorelaxation reported for its parent chemotype in airway models. These findings establish ligand-induced GPCR homodimerization as a distinct nondegrading molecular-glue mechanism with potential relevance to asthma and chronic obstructive pulmonary disease.

### 7.5. Trapping of Transporter–Substrate Complexes in Bacteria

Zosurabalpin is a tethered macrocyclic peptide antibiotic that inhibits lipopolysaccharide transport in carbapenem-resistant *Acinetobacter baumannii*. Genetic and structural studies identified the inner-membrane LptB_2_FG transporter as its functional target. Cryo-electron microscopy showed that zosurabalpin and related macrocycles bind within the central transporter cavity while simultaneously contacting a bound LPS molecule [152]. The compound, therefore, recognizes a composite protein–substrate interface and traps LPS in a nonproductive transport intermediate. This blocks onward transfer through the Lpt pathway, causes toxic intracellular accumulation of LPS, and produces bactericidal activity. Zosurabalpin has retained activity against multidrug-resistant isolates, shown efficacy in several murine infection models, and entered phase I clinical development [153,154]. Its narrow spectrum reflects species-specific features of LptFG, while resistance substitutions in LptF or LptG may affect the durability of this mechanism. Nevertheless, spontaneous resistance frequencies were within the range reported for current antibiotics against *A. baumannii*, and some resistance routes, notably lpxM mutations, were associated with markedly reduced virulence [153].

### 7.6. Stabilization of RNA–Protein Interfaces

Rocaglates and the clinical derivative zotatifin stabilize eIF4A on polypurine motifs within mRNA 5′UTRs, converting the helicase into a sequence-selective translational repressor [155]. This RNA–protein gluing mechanism blocks ribosomal scanning and preferentially suppresses transcripts encoding oncogenic and pro-survival proteins, including KRAS, MYC, MCL1, cyclins, and selected receptor tyrosine kinases. Zotatifin has shown activity in RTK-driven tumors and prostate cancer models, where inhibition of AR, AR-V7, and HIF1A translation enhances responses to anti-androgen therapy and radiotherapy [156]. It has also entered phase 1/2 evaluation in advanced solid tumors and ER+/HER2- metastatic breast cancer [157]. By stabilizing a protein–nucleic acid interface rather than a protein–protein complex, this class broadens nondegrading molecular-glue pharmacology toward transcript-selective control of translation.

## 8. Concluding Remarks and Future Perspectives

Monovalent nondegrading molecular glues are evolving from serendipitous discoveries into a major class of proximity-based therapeutics. Their main pharmacological advantage is the ability to induce new protein–protein interfaces and stabilize functional protein states that traditional occupancy-based inhibitors cannot reach. As shown across various disease models, ndMGs can modulate biological pathways through diverse mechanisms, such as trapping proteins in specific cellular locations, restoring the function of mutated proteins, and blocking pathogen assembly.

However, a major gap remains between a few successful clinical candidates and a large number of early-stage chemical probes. To address this issue, the field needs to move from identifying molecular glues by chance to rational, prospective drug design. Future progress will depend on developing screening assays that measure ternary complex formation rather than simple binary affinity. Combining structural biology, chemoproteomics, DNA-encoded libraries, and new screening techniques can help find targetable protein interfaces. In addition, computational tools and machine learning models for protein–protein interaction may accelerate candidate optimization.

Translating these scientific advances into clinical practice involves several key challenges. First, small molecules must be selective for the target interface to avoid toxicities, especially when targeting wild-type proteins or highly conserved chaperone systems. Second, mutations at the induced interface can lead to drug resistance, which reduces therapeutic efficacy. To overcome these problems, new discovery platforms should include in vivo biomarkers to quantify ternary complex engagement. Finally, clinical success will require patient stratification strategies based on target abundance and pathway dependencies. In conclusion, nondegrading molecular glues provide an important therapeutic strategy that complements conventional small-molecule inhibitors and targeted protein degradation.

## Figures and Tables

**Table 1:** Representative monovalent nondegrading molecular glues in cancer research and drug development.

Compound	Biomolecular Assembly	Functional Consequence	Developmental Stage
Suppressing Proliferative Signaling			
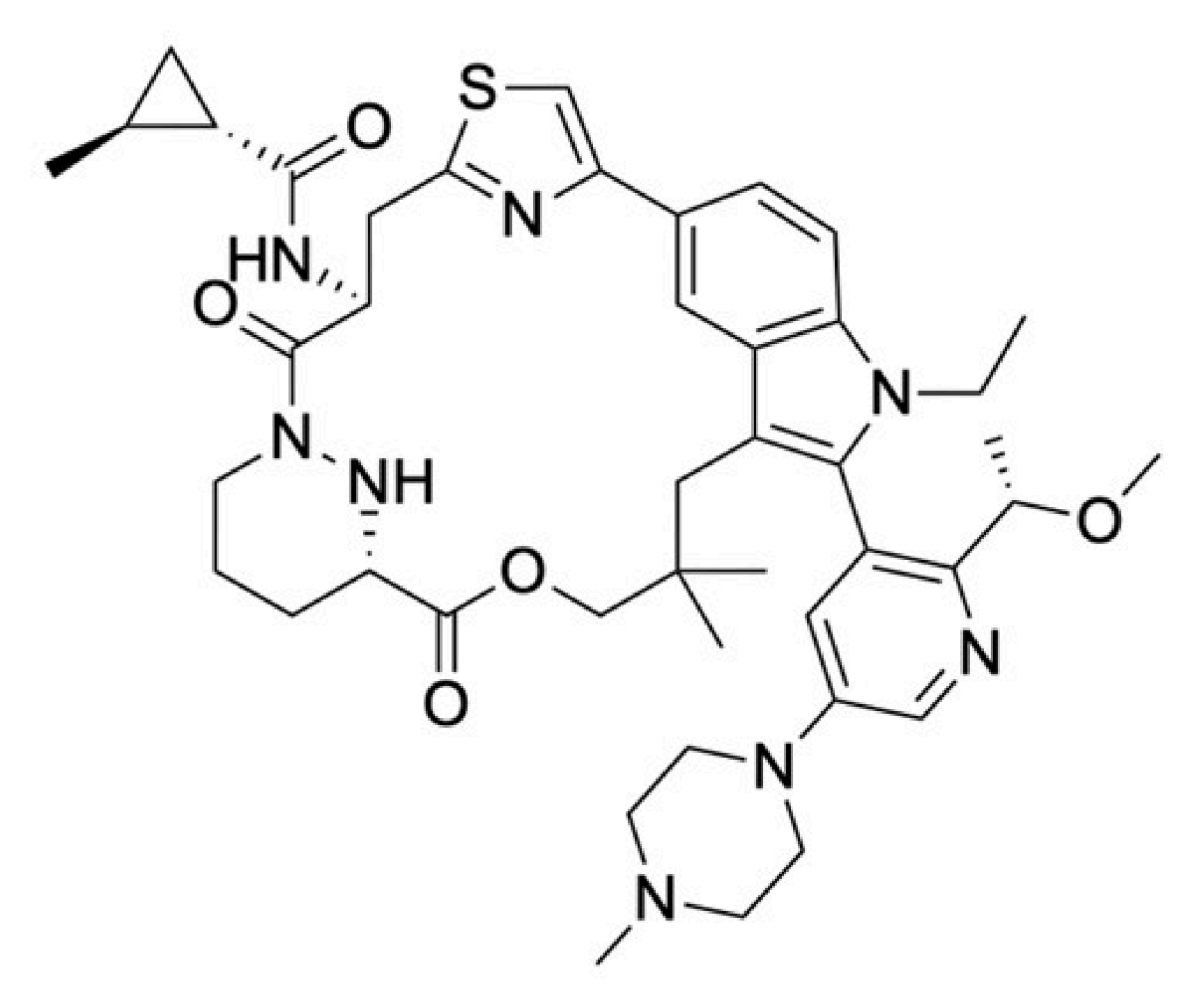 Daraxonrasib (RMC-6236)	CypA–RAS(ON); multi-selective for mutant and wild-type RAS	RAS effector blockade	Phase 3
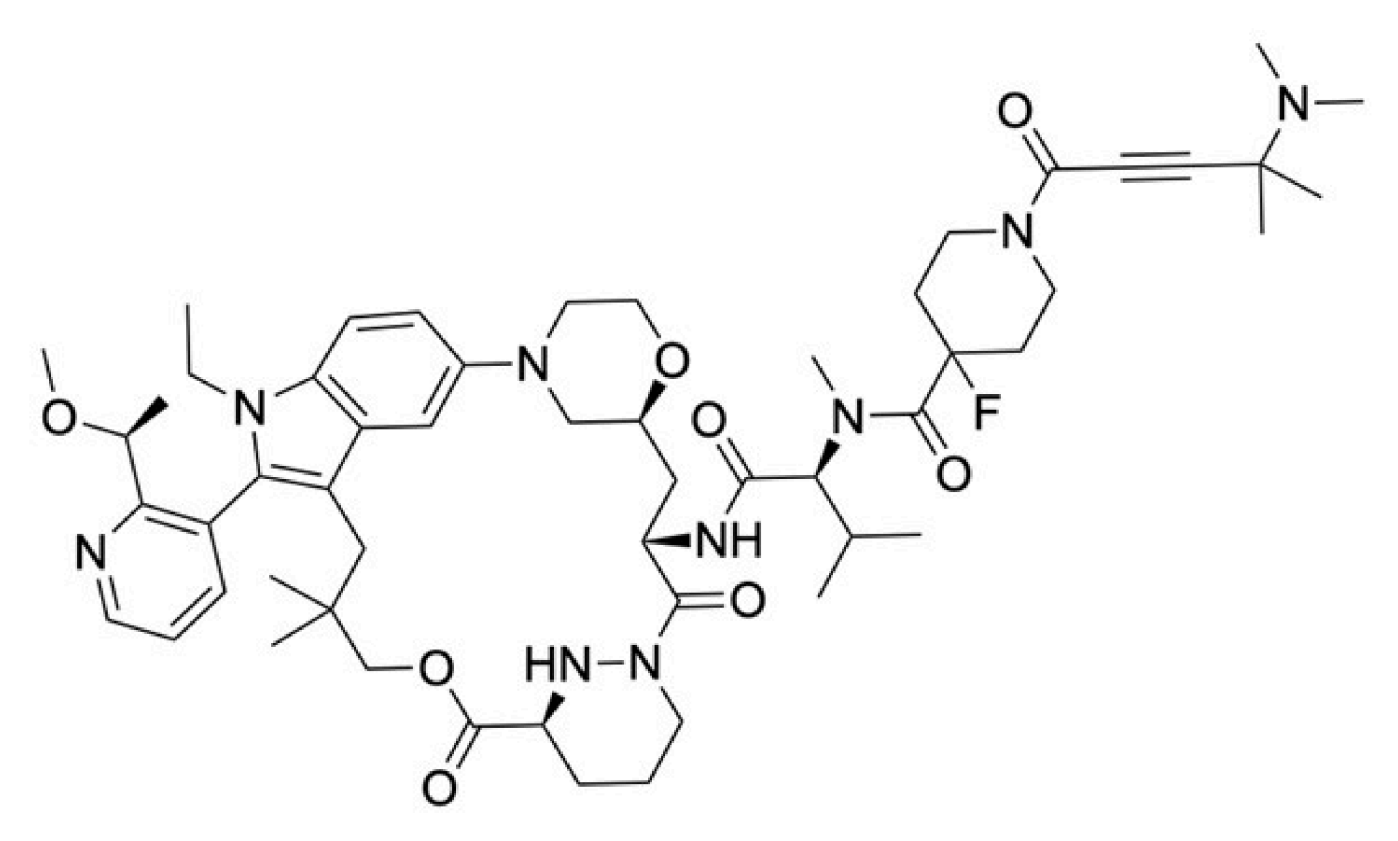 Elironrasib (RMC-6291)	CypA–KRAS^G12C^(ON)	RAS effector blockade	Phase 1/1B (monotherapy); Phase 1b/2 (combination therapy)
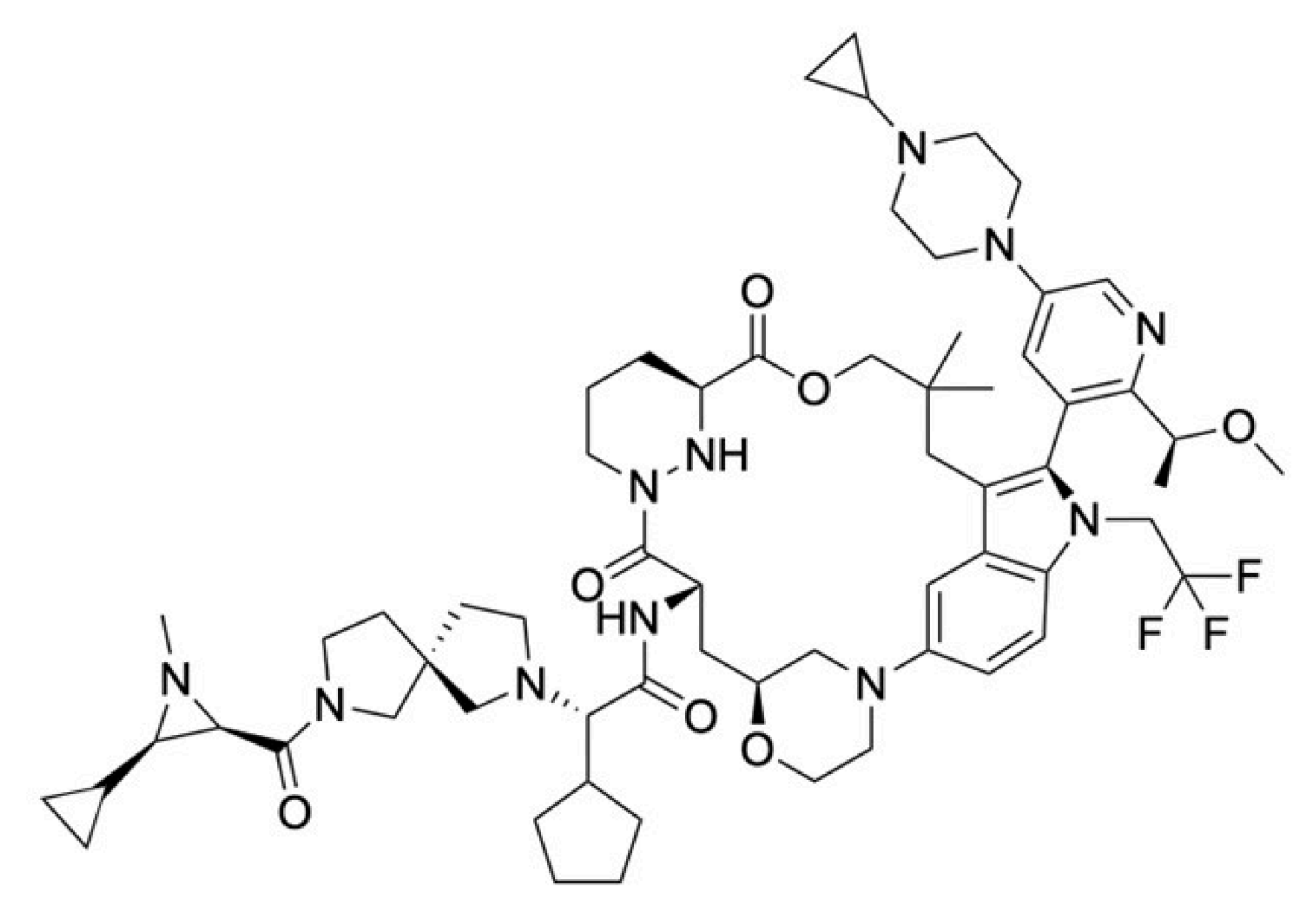 Zoldonrasib (RMC-9805)	CypA–KRAS^G12D^(ON)	RAS effector blockade	Phase 1/1b (monotherapy); Phase 1b/2 (combination therapy); Phase 3 (PDAC)
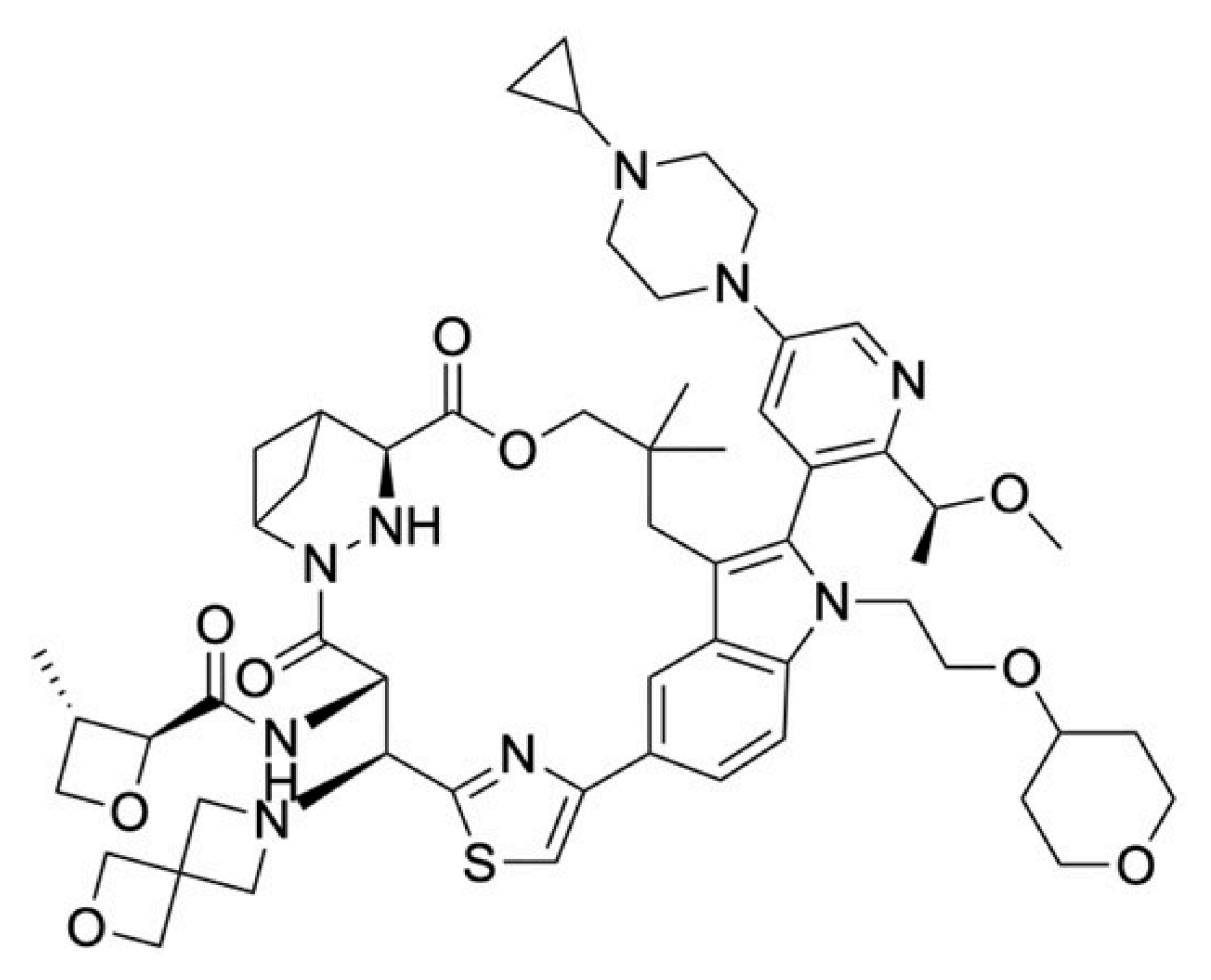 RMC-5127	CypA–KRAS^G12V^(ON)	RAS effector blockade	Phase 1/1b
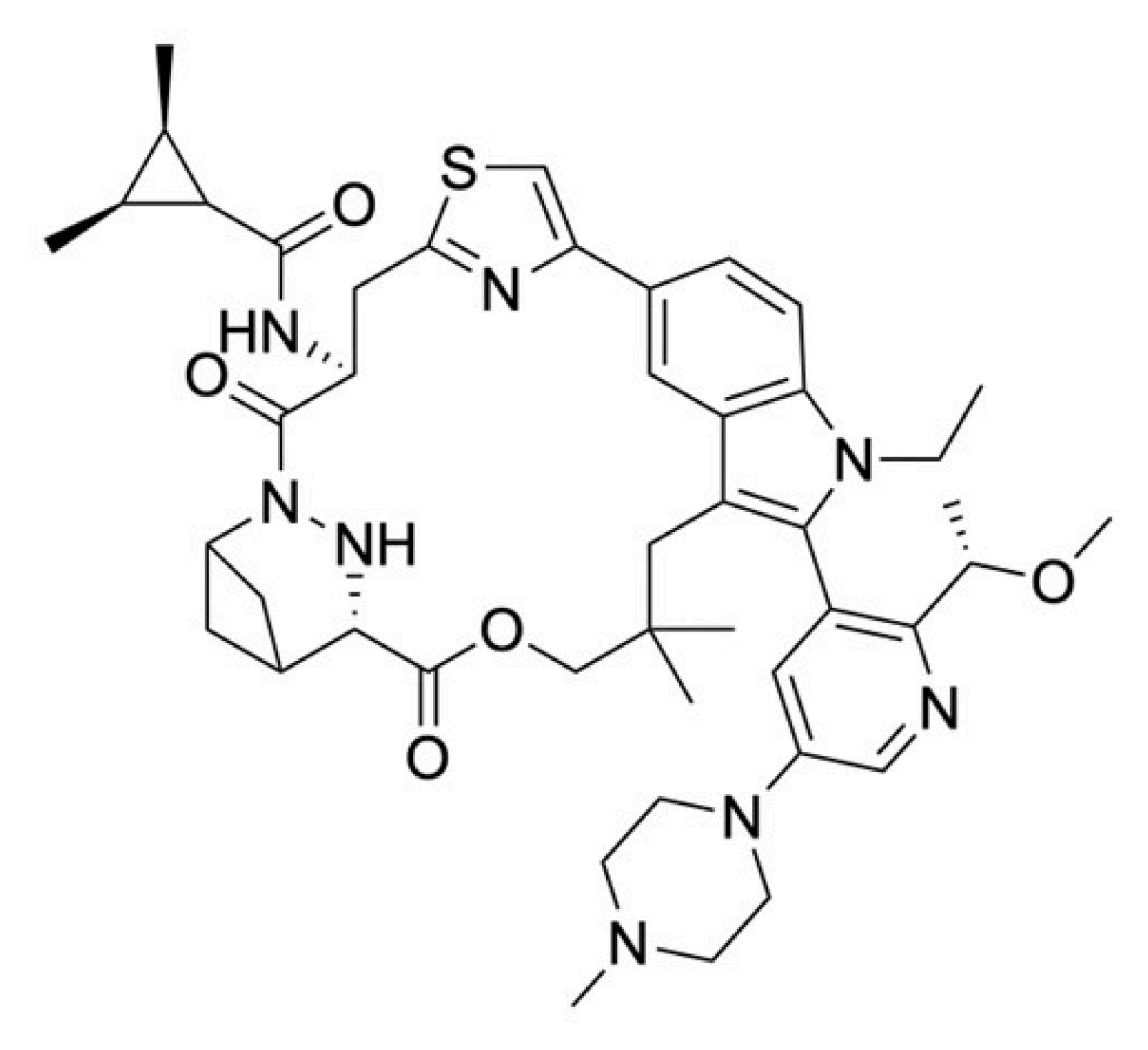 ERAS-0015	CypA–RAS(ON); pan-RAS	RAS effector blockade	Phase 1
GFH-276 structure undisclosed	CypA–RAS(ON); pan-RAS	RAS effector blockade	Phase 1/2 (monotherapy); Phase 1b/2 (combination therapy)
JAB-23E73 structure undisclosed	CypA–RAS(ON)	RAS effector blockade	Phase 1/2a
HEC228032 structure undisclosed	CypA–RAS(ON); pan-RAS	RAS effector blockade	Preclinical (cellular, in vivo validation)
SP09253 structure undisclosed	CypA–RAS(ON);	RAS effector blockade	Preclinical (cellular, in vivo validation)
RCZY-690 structure undisclosed	CypA–RAS(ON); pan-RAS	RAS effector blockade	Preclinical (IND filing announced)
HZ-V068 structure undisclosed	CypA–RAS(ON)	RAS effector blockade	Preclinical (cellular, in vivo validation)
HZ-V055 structure undisclosed	CypA–RAS(ON)	RAS effector blockade	Preclinical (IND filed)
p120RasGAP glues name and structure undisclosed	KRAS–p120RasGAP	RAS inactivation	Preclinical (biochemical, cellular validation)
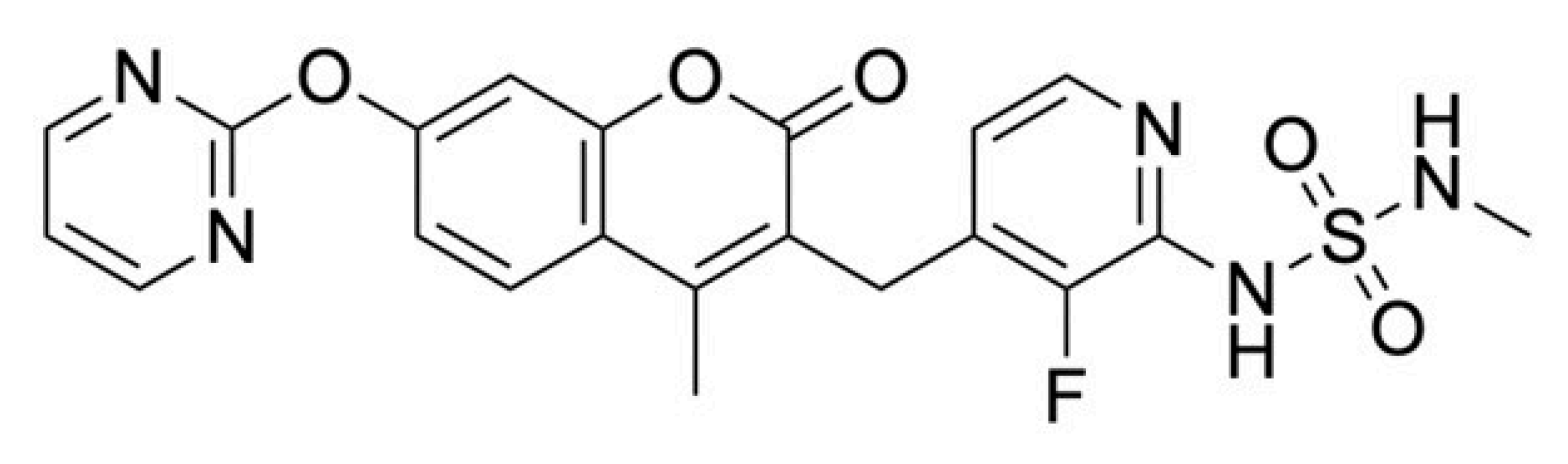 Avutometinib (VS-6766/CH5126766/ RO5126766)	Pan-RAF–MEK	MEK activation blockade	Approved
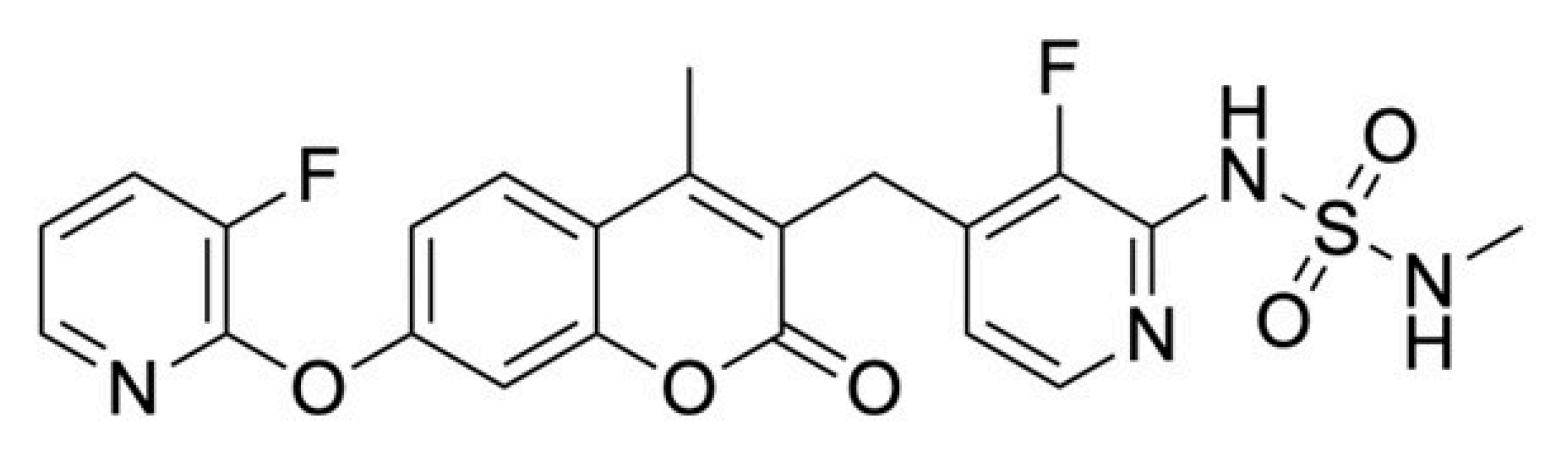 NST-628	Pan-RAF–MEK	MEK activation blockade	Phase 1
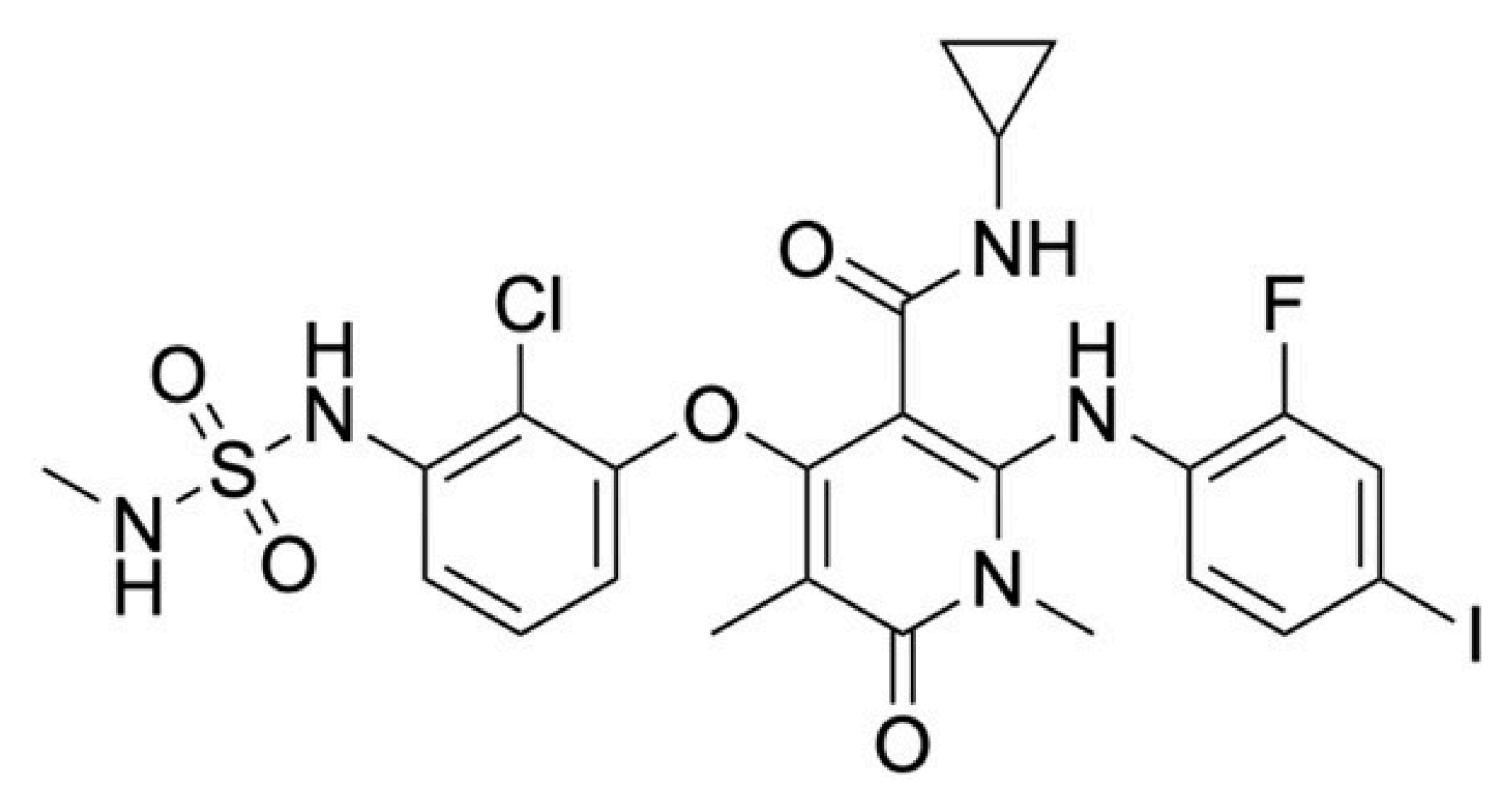 IK-595	Pan-RAF–MEK	MEK activation blockade	Phase 1
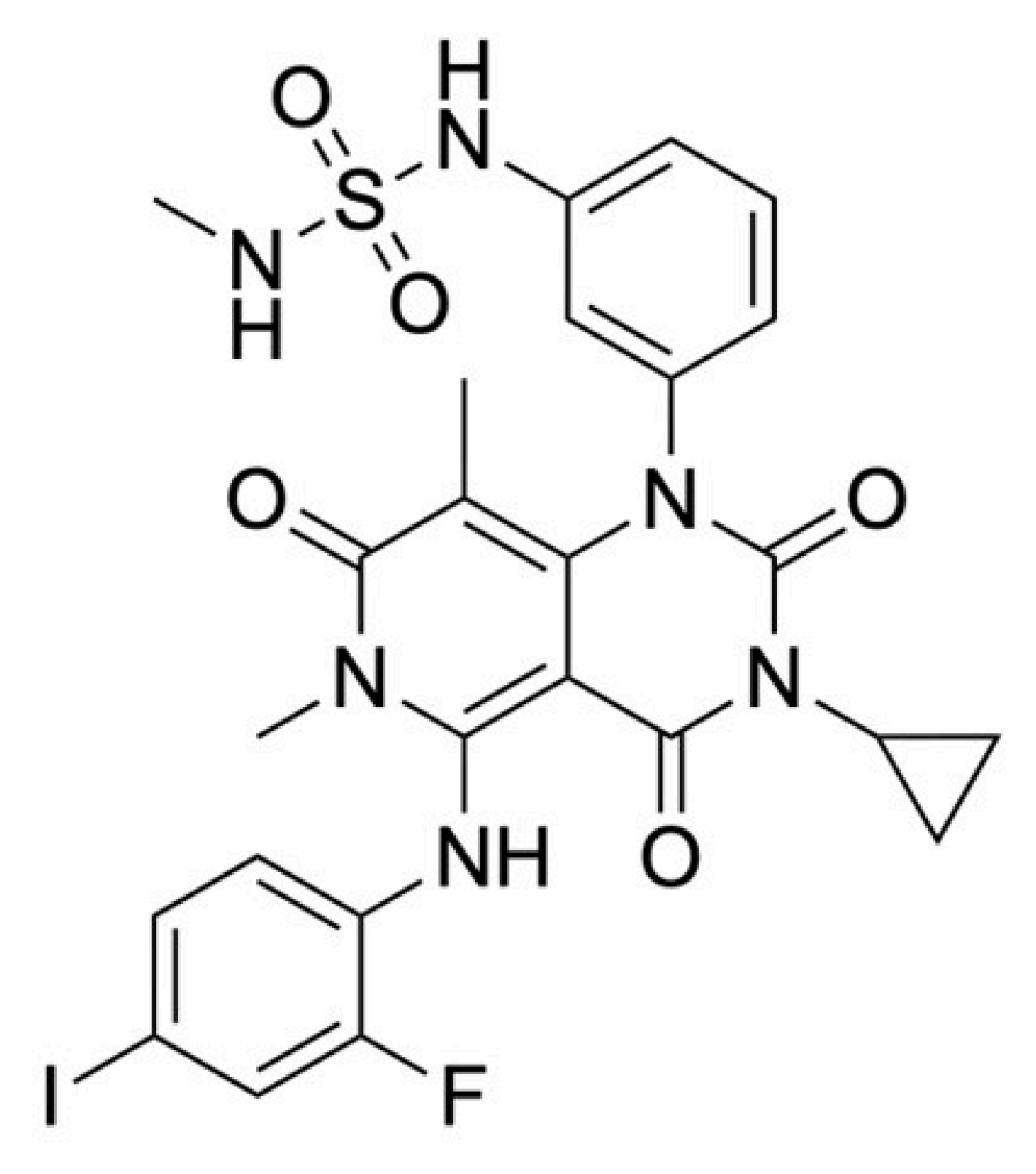 Trametiglue	MEK–KSR and MEK–BRAF	MEK inhibition	Preclinical (biochemical, cellular validation)
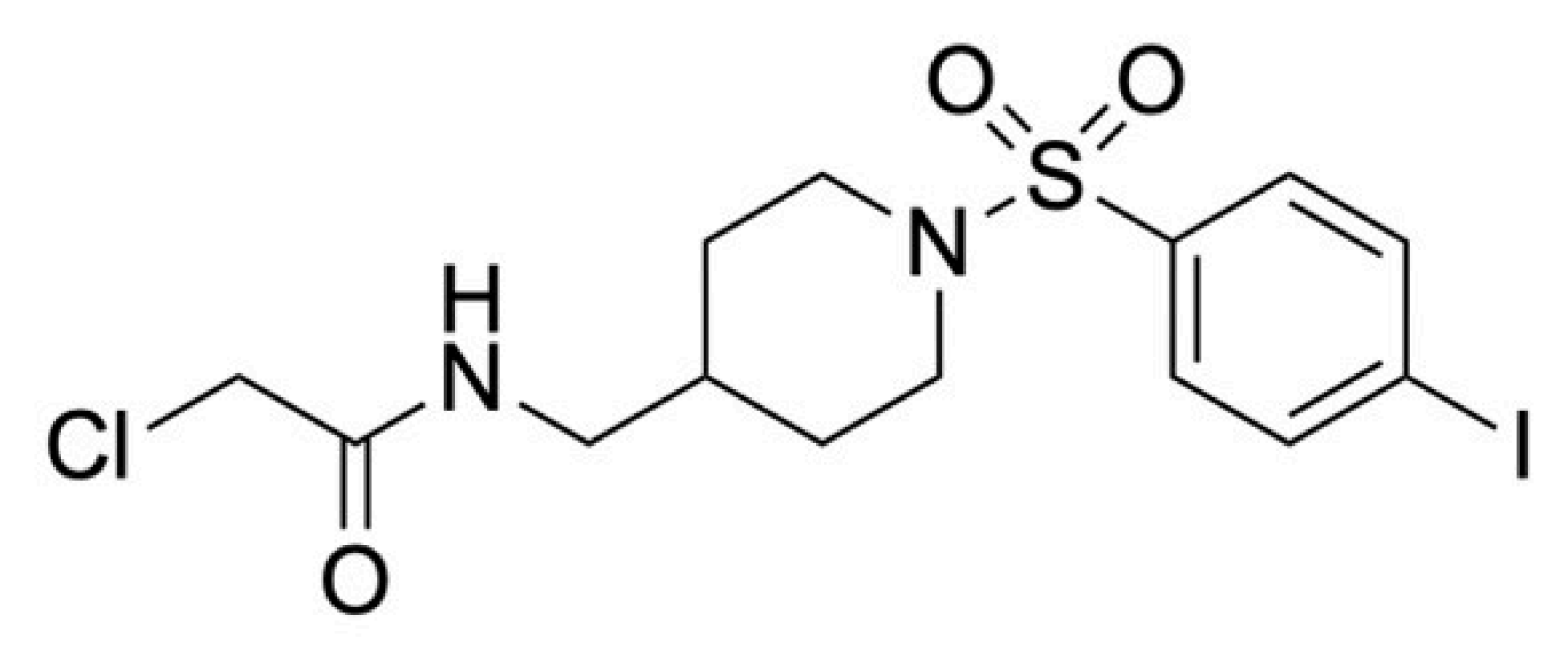 Compound **23**	14-3-3σ–C-RAF(pS259) autoinhibitory complex	Reinforcement of RAF autoinhibition	Preclinical (biochemical, cellular validation)
Reactivating Cell Death Programs			
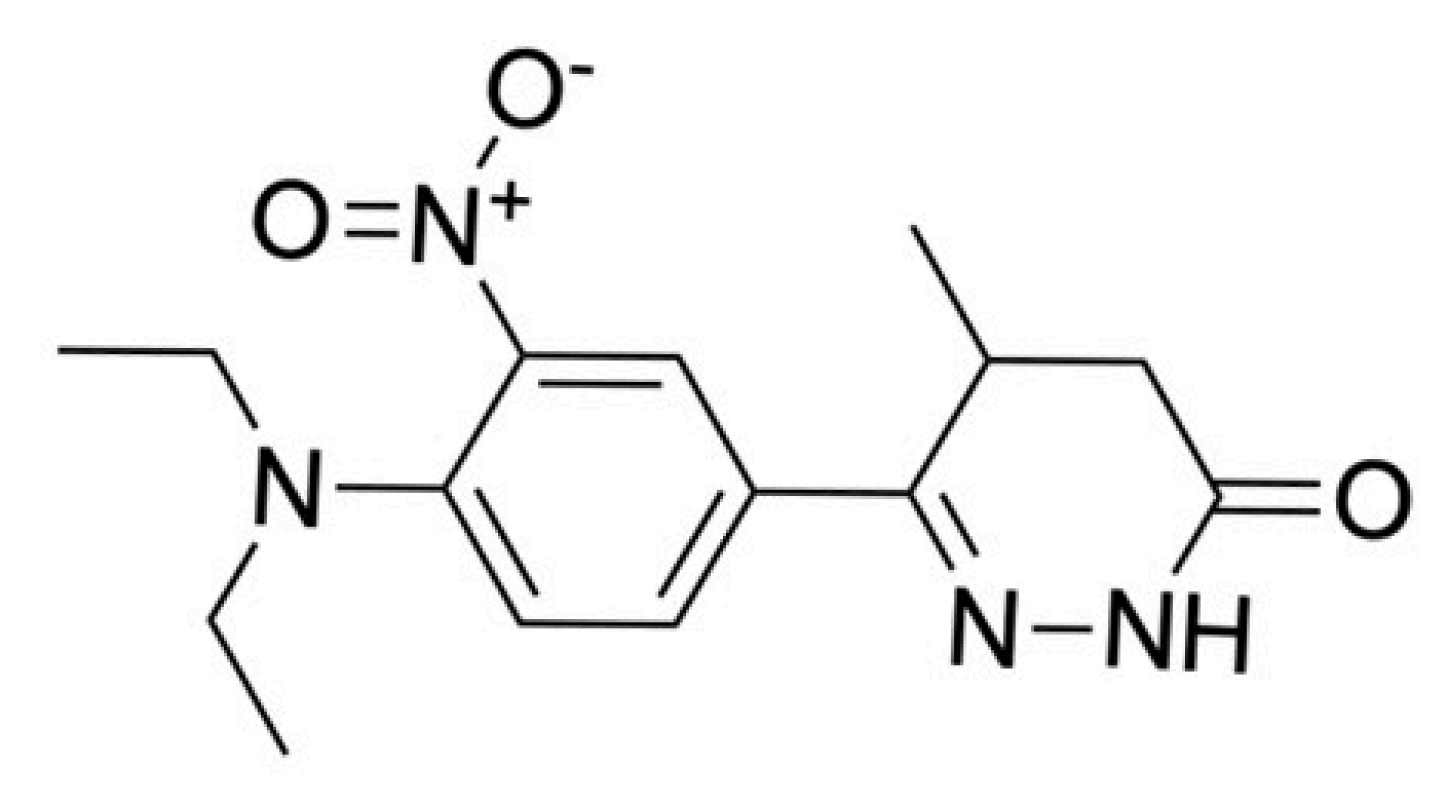 DNMDP	PDE3A–SLFN12	SLFN12 activation and tomoptosis	Preclinical (biochemical, cellular validation)
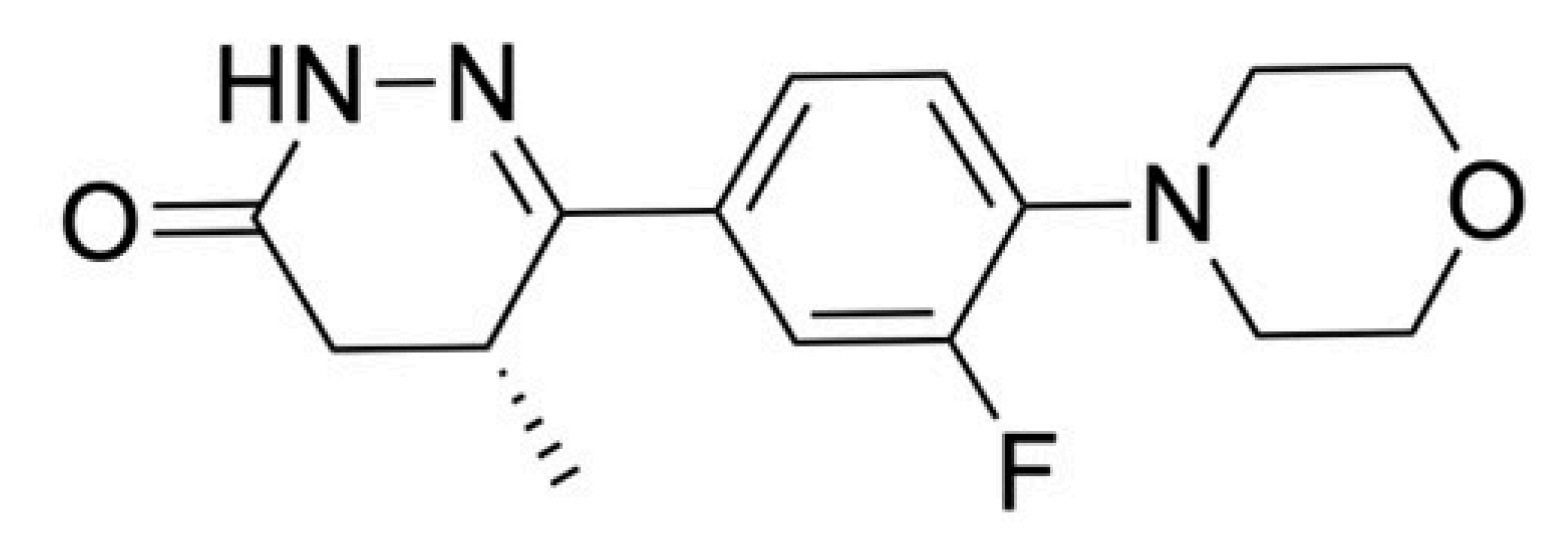 BRD9500	PDE3A–SLFN12	SLFN12 activation and tomoptosis	Preclinical (cellular, in vivo validation)
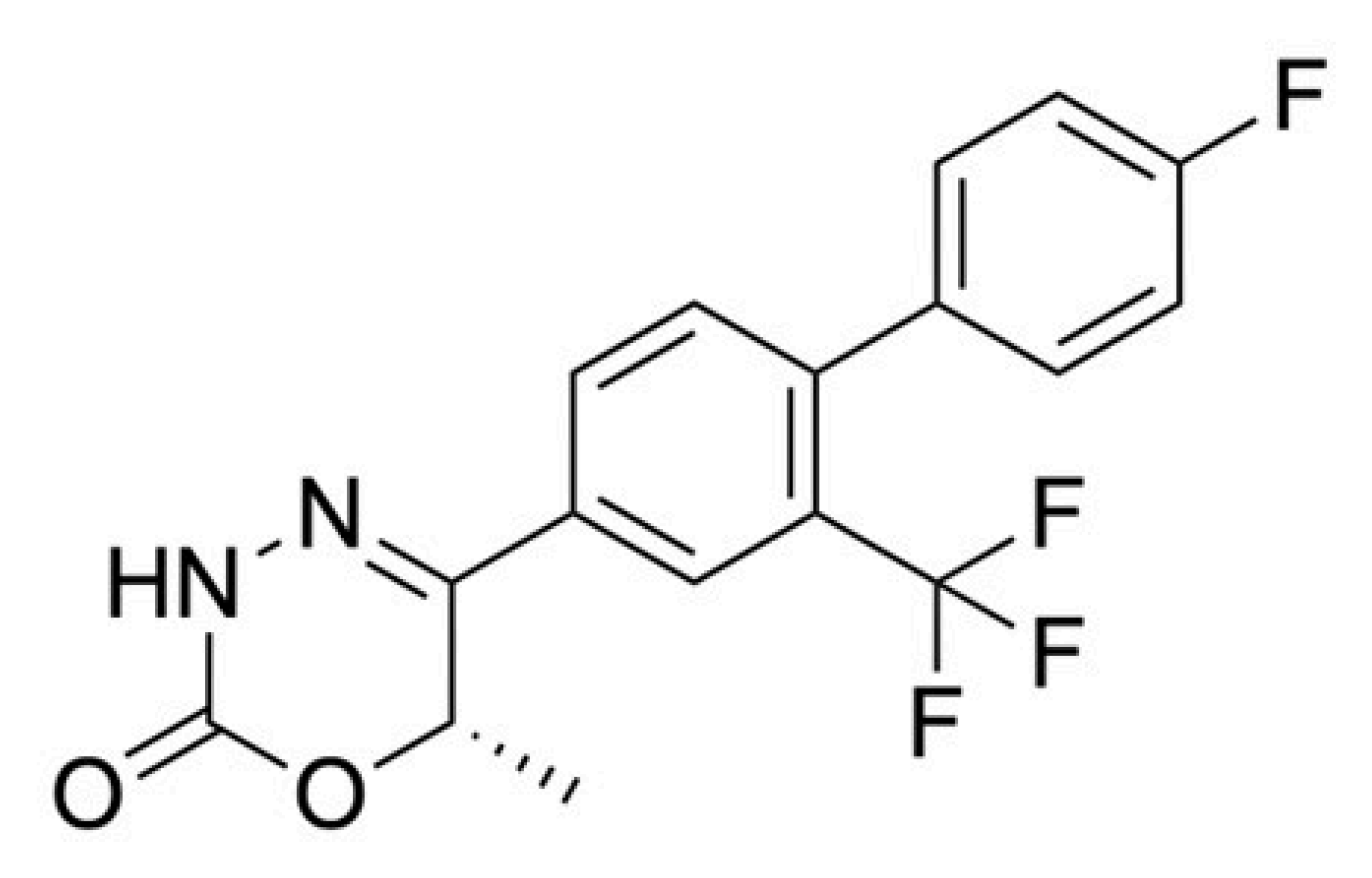 BAY 2666605	PDE3A–SLFN12	SLFN12 activation and tomoptosis	Phase 1 terminated
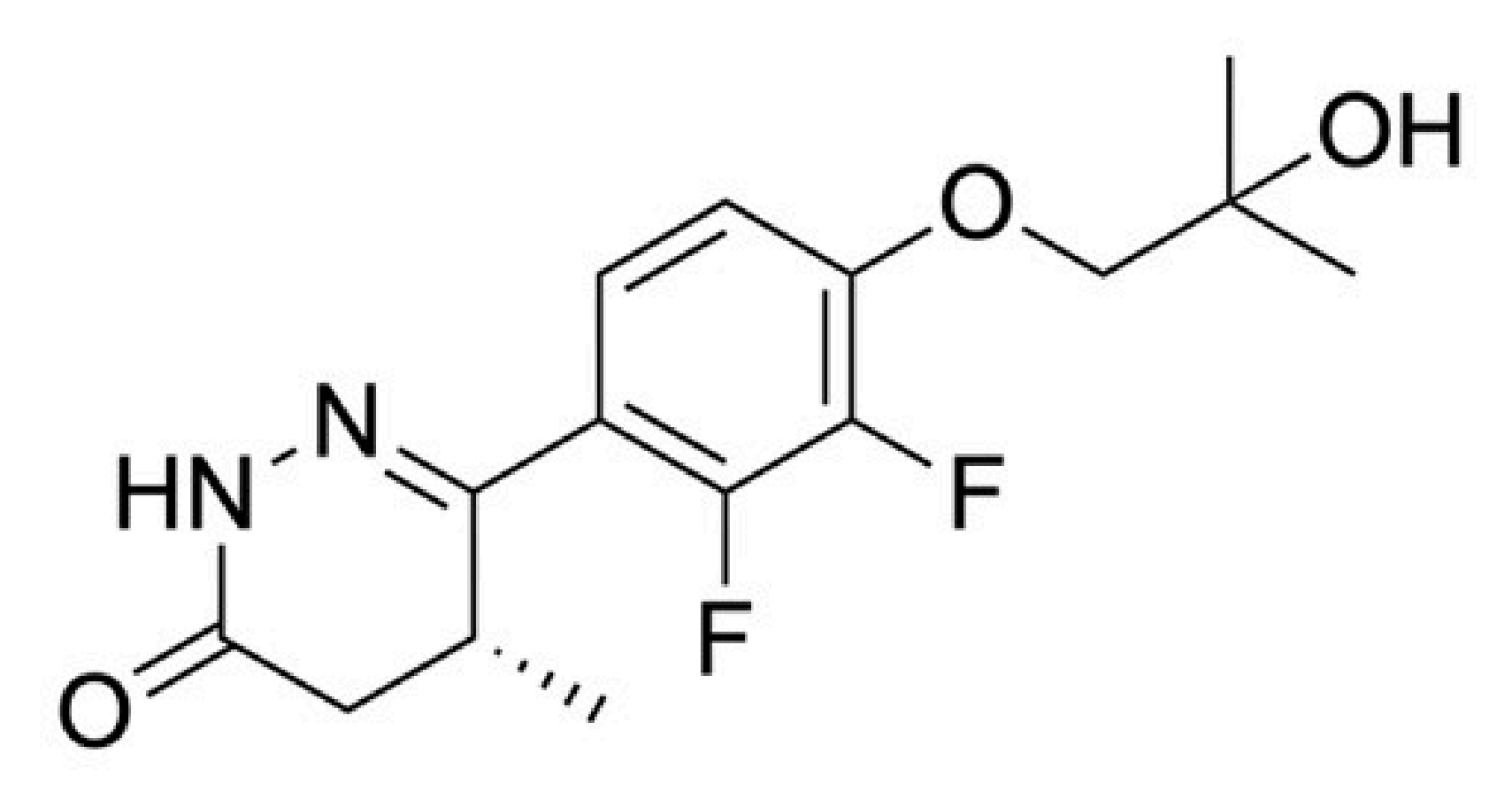 OPB-171775	PDE3A–SLFN12	SLFN12 activation and tomoptosis	Phase 1-ready
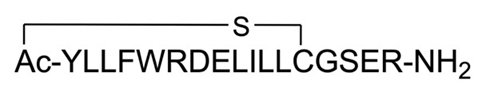 5L1	MCL1–MCL1 homodimer	MCL1 inactivation by homodimerization	Preclinical (biochemical, cellular validation)
Restoring Growth-Suppressive Functions			
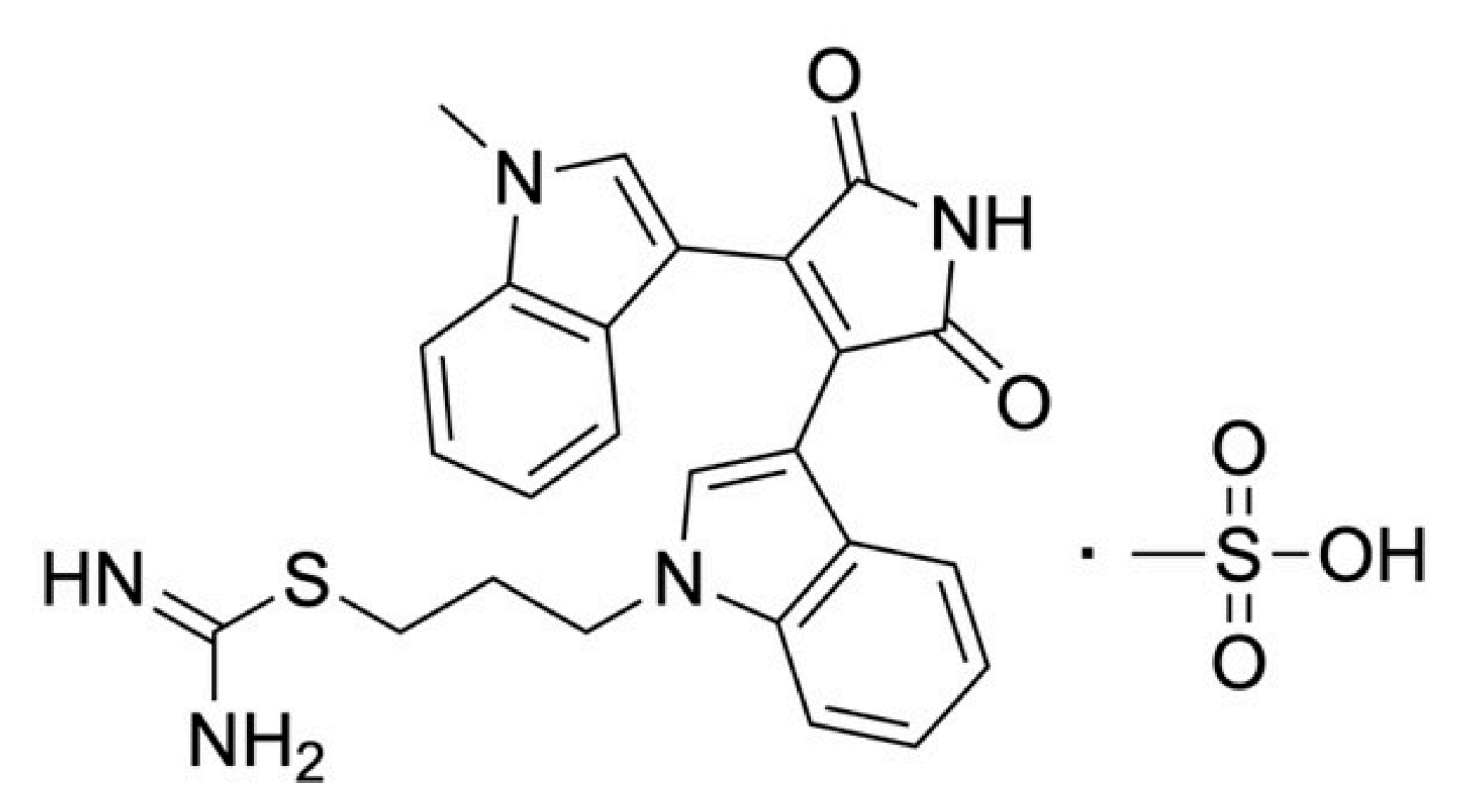 Ro-31-8220	SMAD4^R361H^–SMAD3	Restoration of SMAD signaling	Preclinical (biochemical, cellular validation)
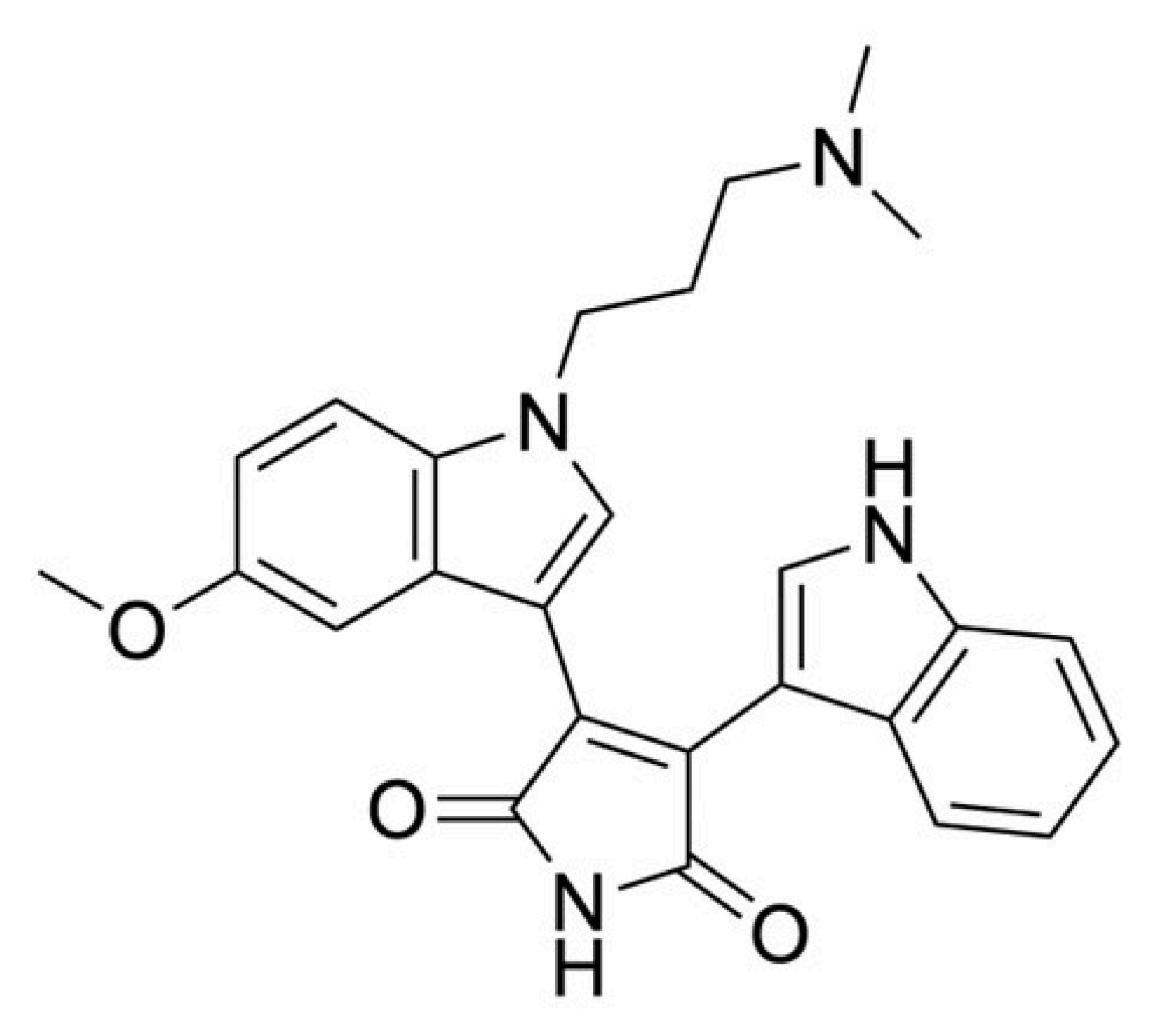 Go-6983	SMAD4^R361H^–SMAD3	Restoration of SMAD signaling	Preclinical (biochemical, cellular validation)
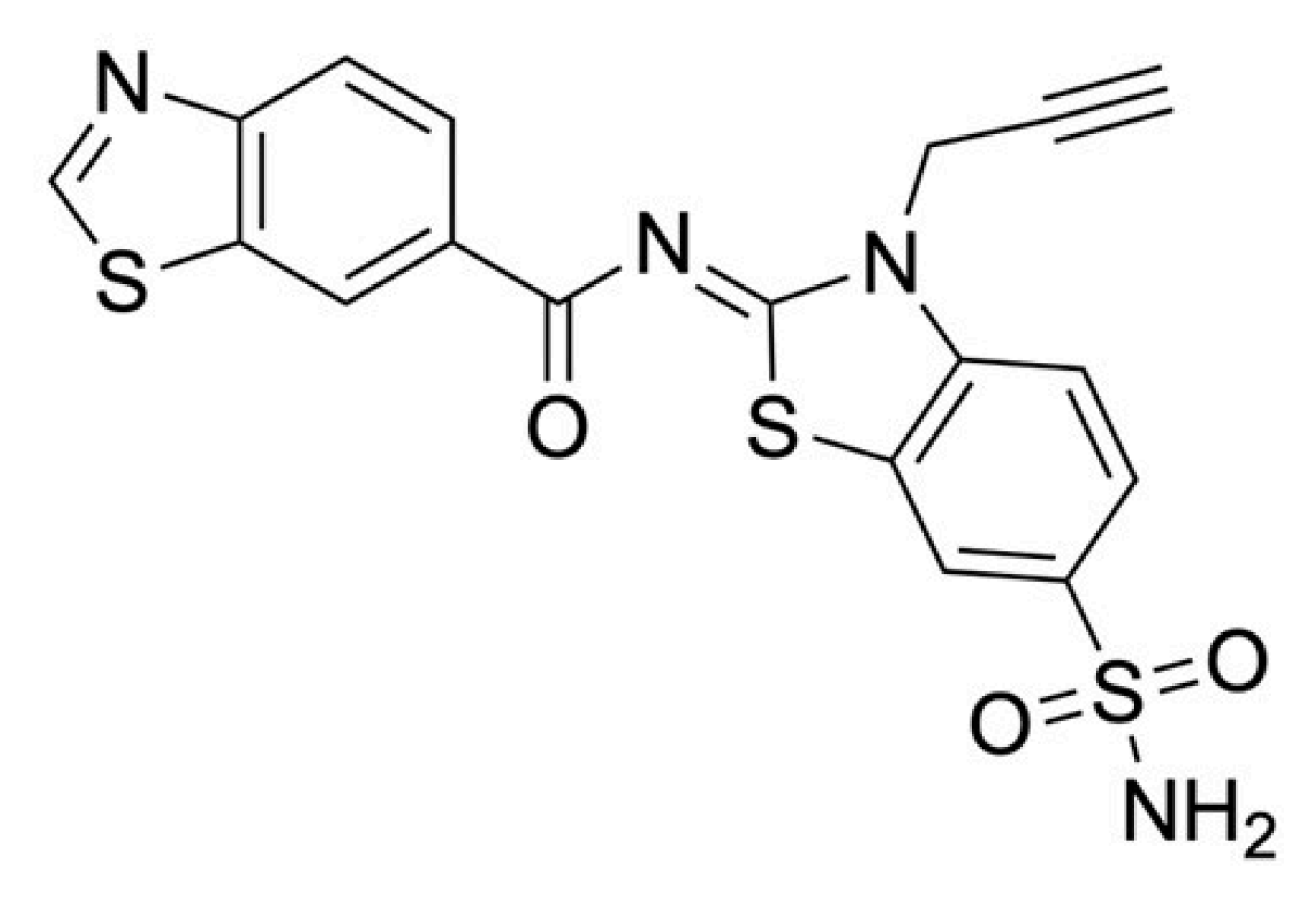 neoCMG101	SMAD4^R361C^–SMAD3	Restoration of SMAD signaling	Preclinical (biochemical, cellular validation)
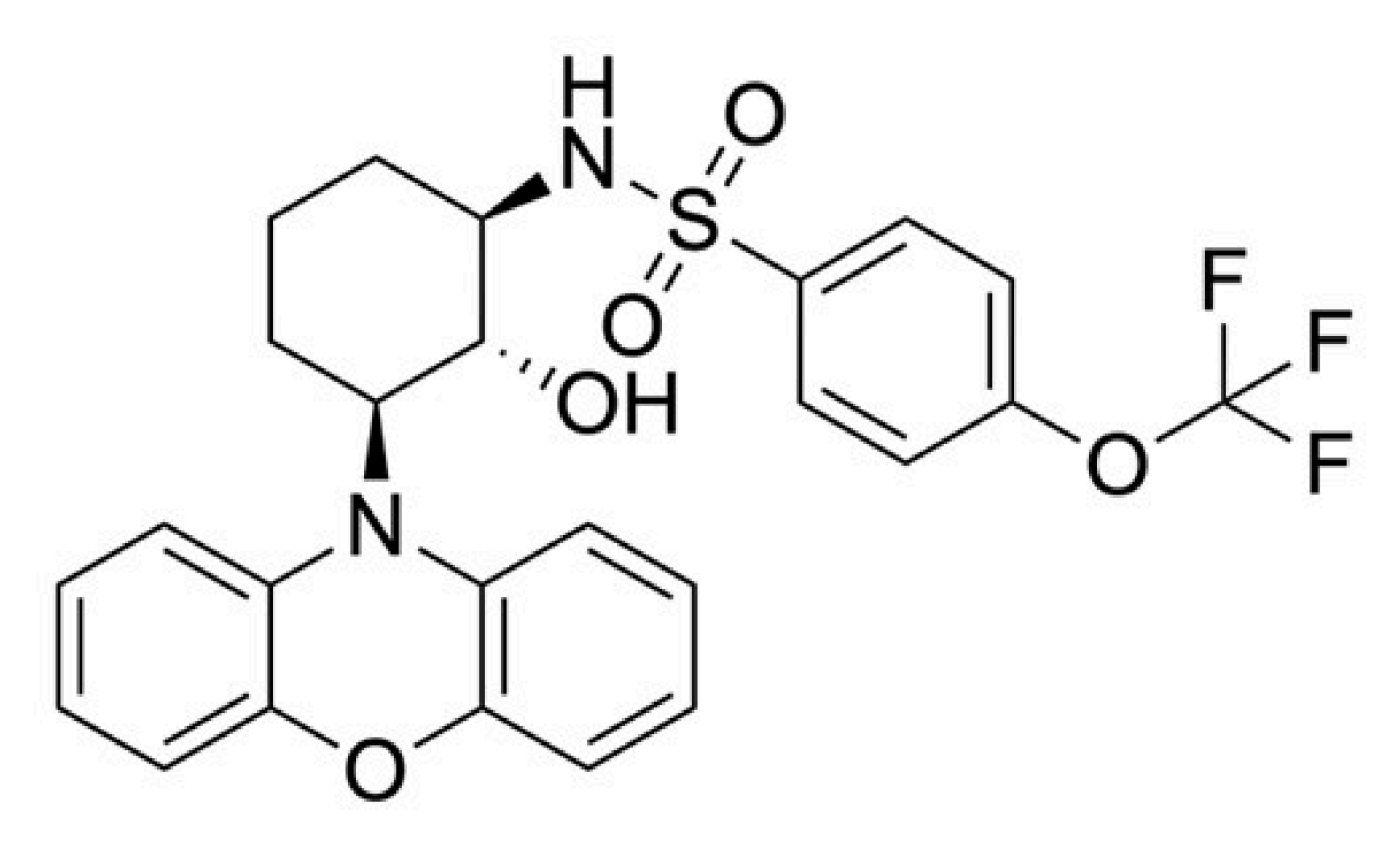 DT-061 (SMAP-061)	PP2A A–C–B56α heterotrimer	PP2A-B56α activation	Preclinical (biochemical, cellular validation)
RPT04402 (SW-3431) structure undisclosed	PP2A A–C–B56α heterotrimer	PP2A-B56α activation	Preclinical (biochemical, cellular, in vivo validation)
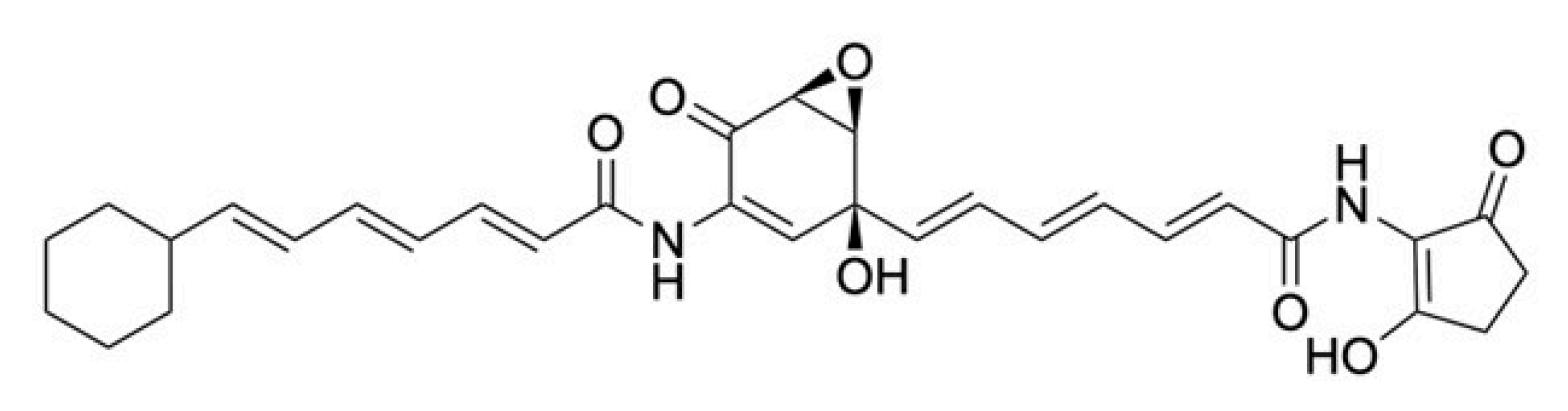 Asukamycin	UBR7-p53	p53 activation	Preclinical (biochemical, cellular validation)
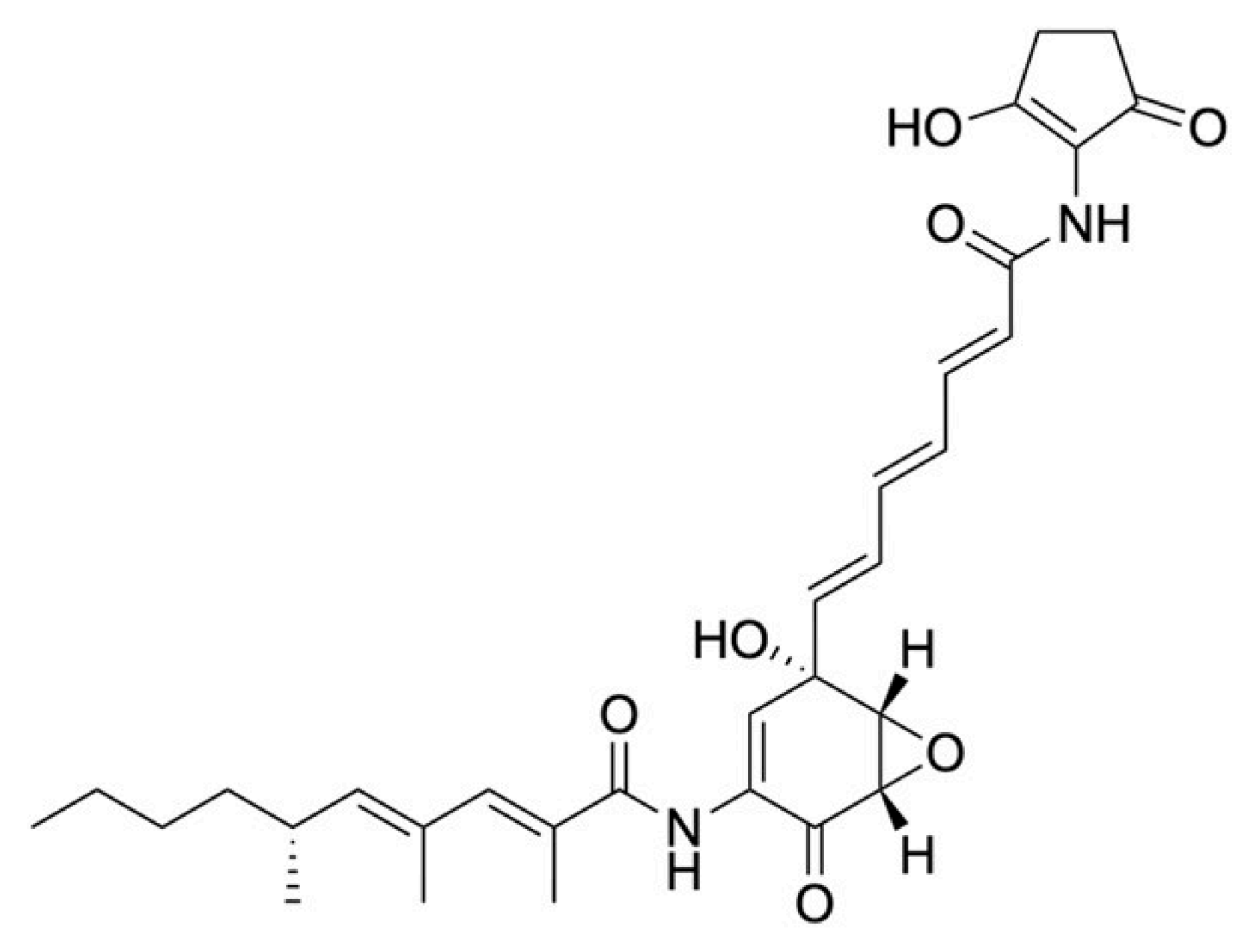 Manumycin A	UBR7–p53	p53 activation	Preclinical (biochemical, cellular validation)
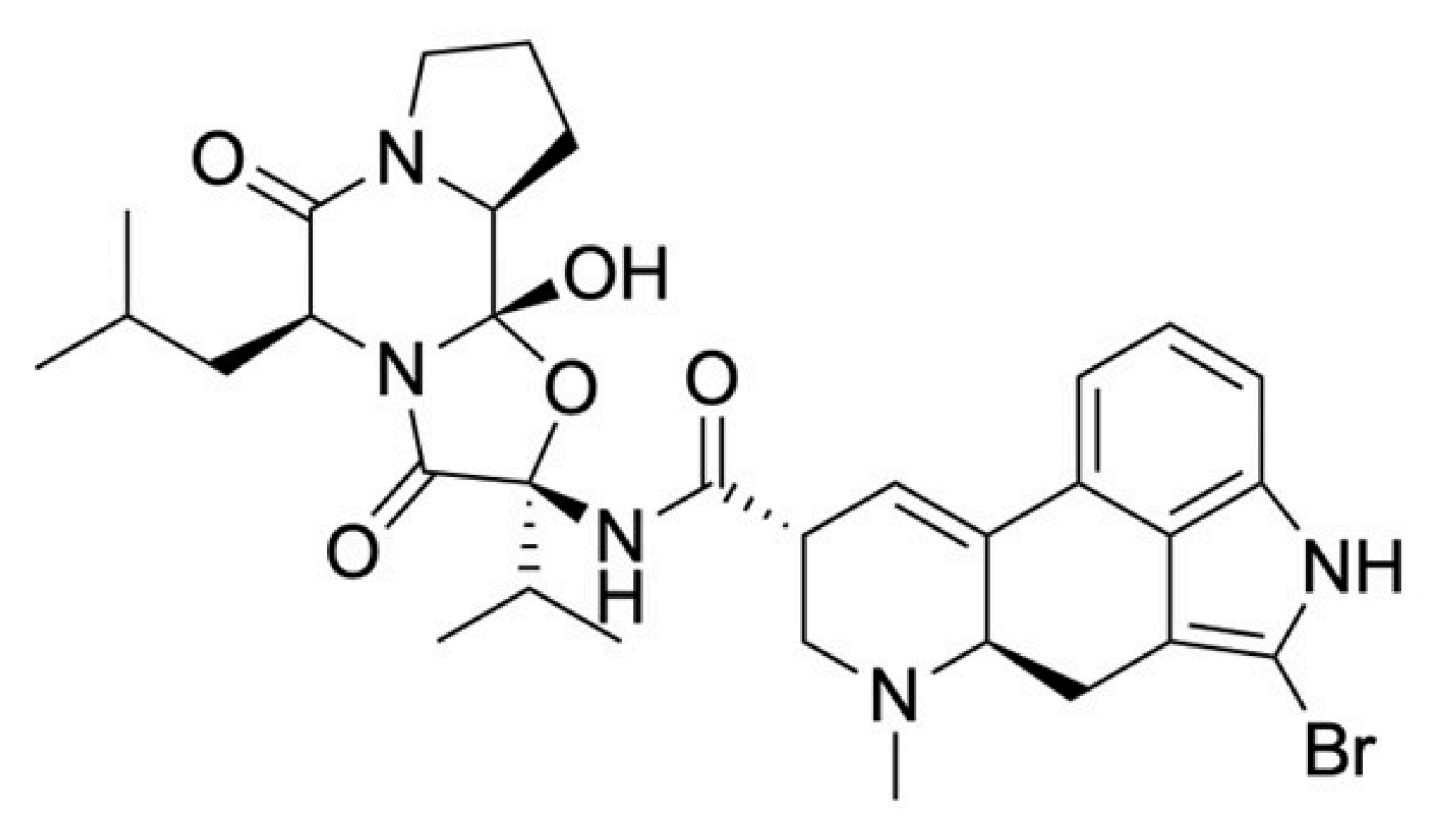 Bromocriptine	USP7–p53	p53 activation	Approved (other indications); preclinical (p53–USP7 glue)
Transcriptional Rewiring			
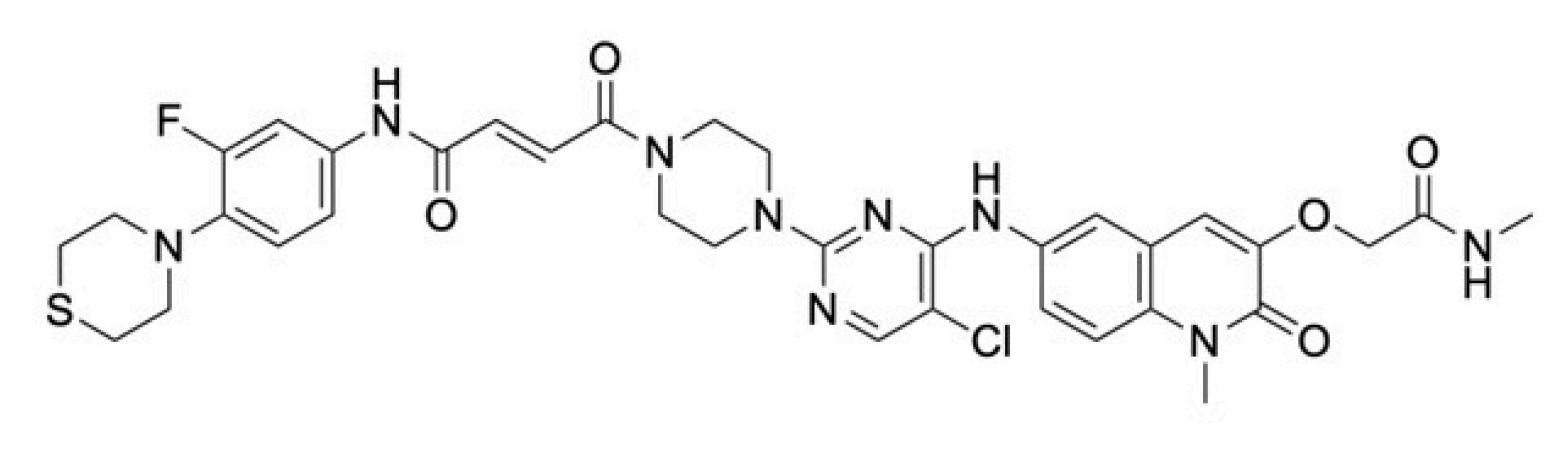 ZD-1-186	BCL6–BRD9	BCL6 transcriptional rewiring	Preclinical (chemoproteomics, cellular validation)
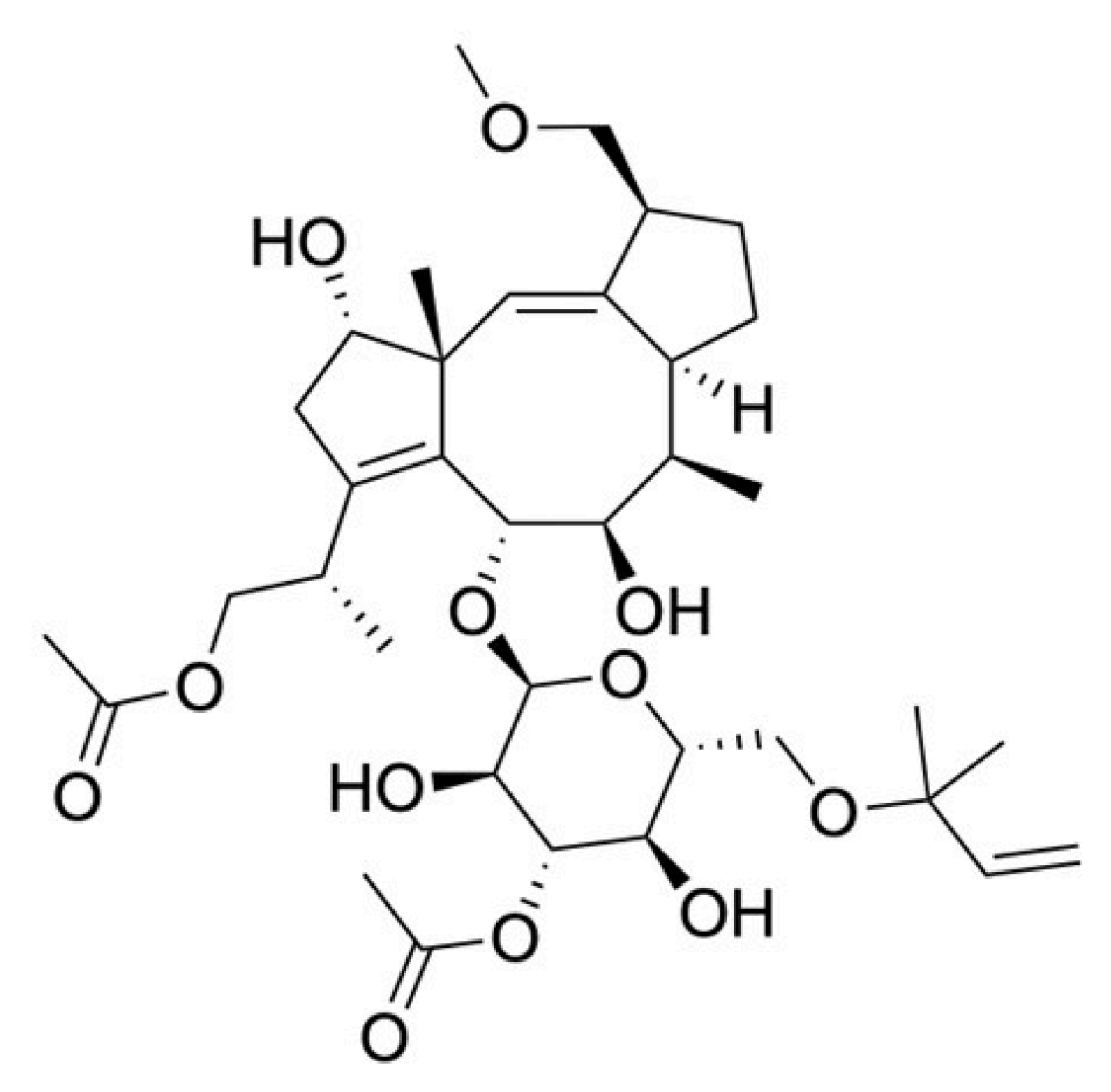 Fusicoccin A	14-3-3–Erα(pT594)	ERα transcriptional inhibition	Preclinical (biochemical, cellular validation)
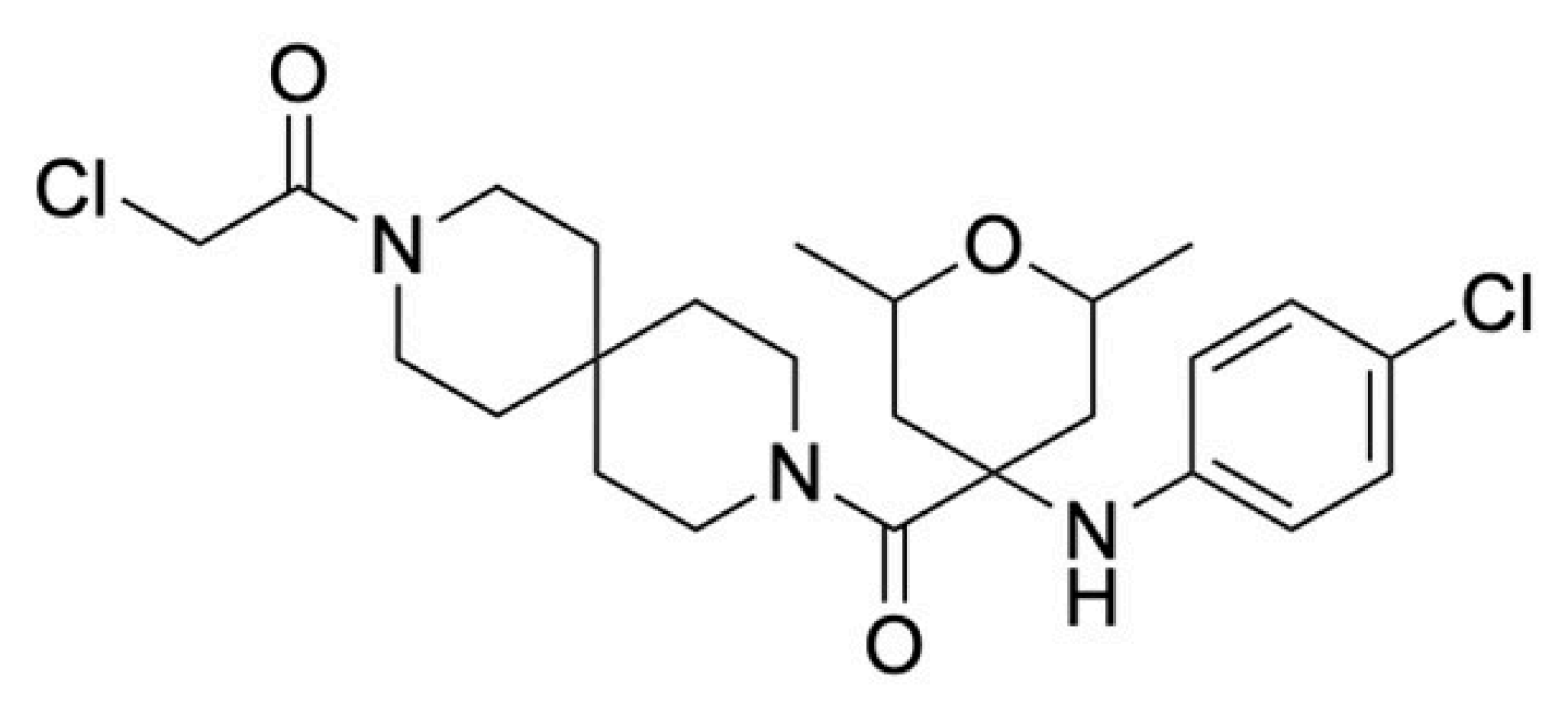 Compound **181**	14-3-3σ–Erα(pT594)	ERα transcriptional inhibition	Preclinical (biochemical, cellular validation)
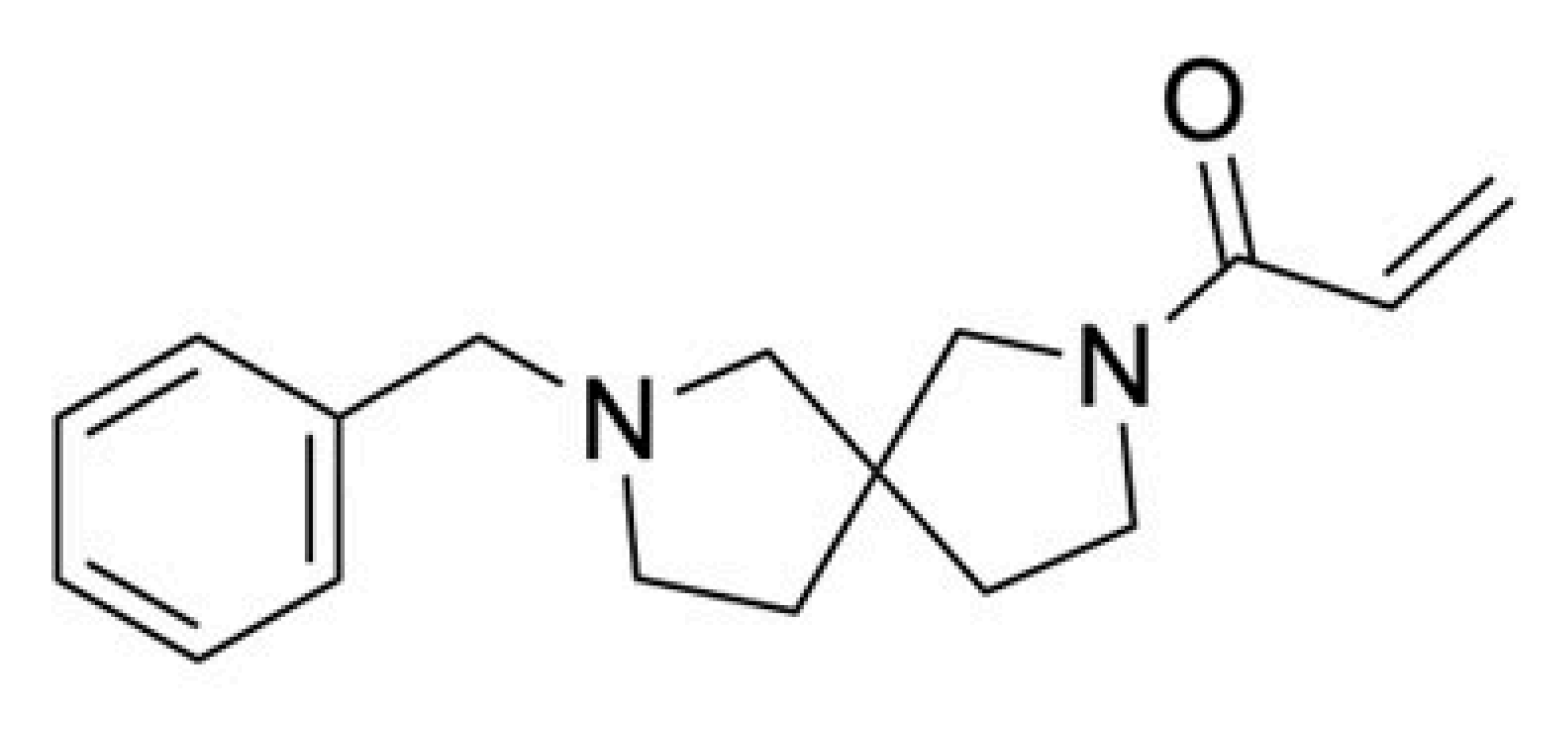 EN171	14-3-3σ–ERα/YAP/TAZ	ERα/YAP/TAZ transcriptional inhibition	Preclinical (biochemical, cellular validation)
Targeting Noncanonical Cancer Dependencies			
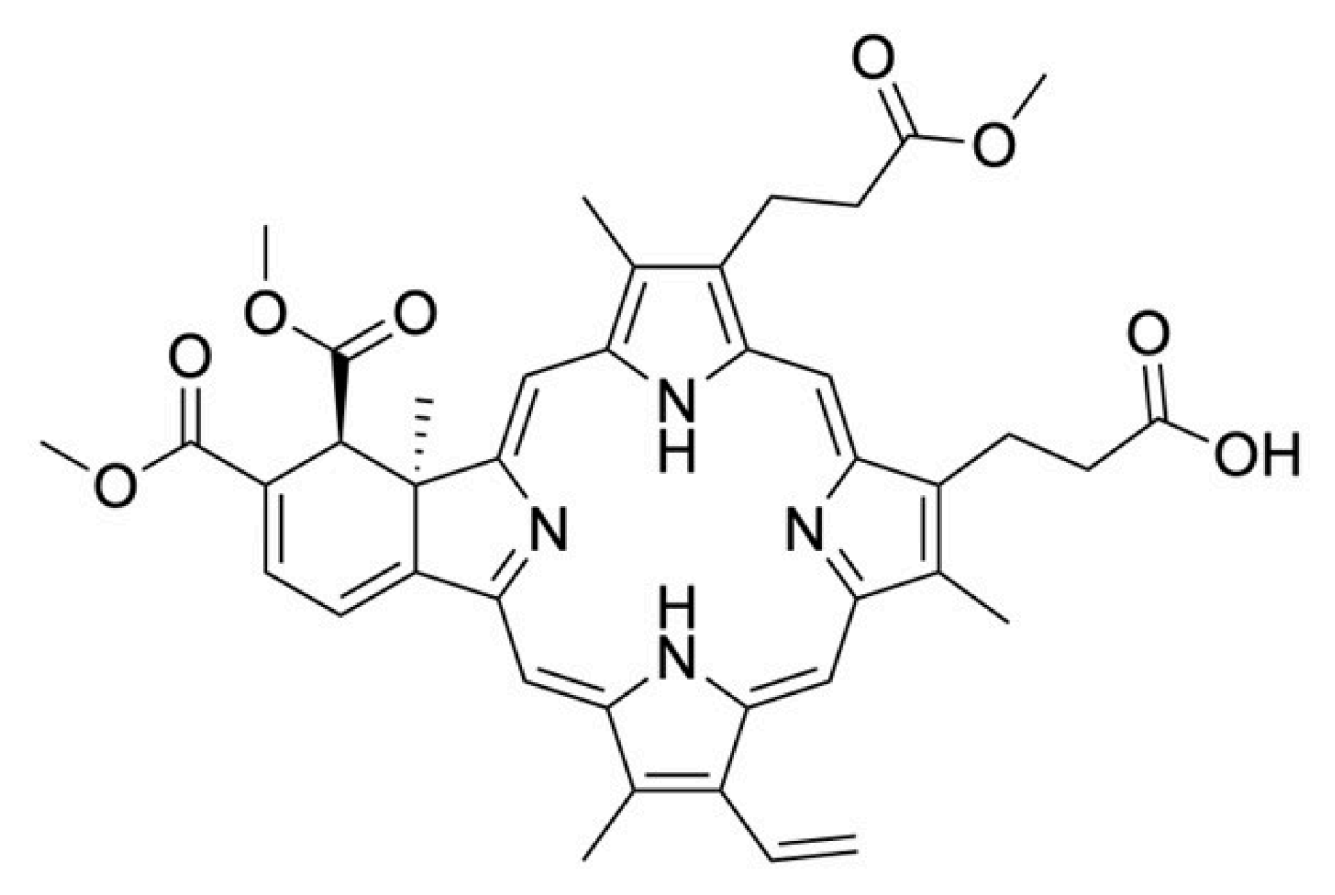 Verteporfin	IRE1α dimer/oligomer	IRE1α activation	Approved (as ophthalmic photosensitizer); preclinical (as IRE1α glue)
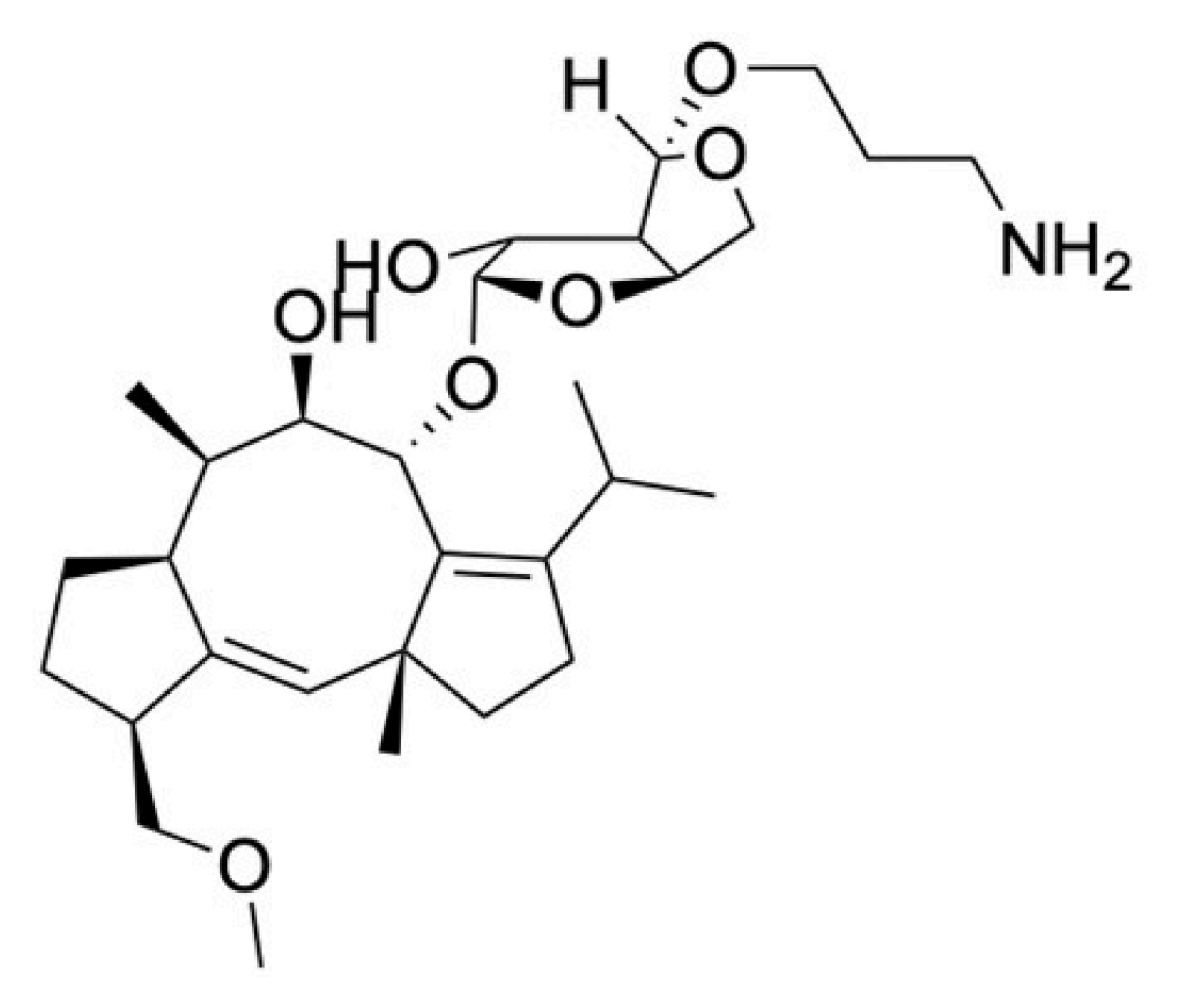 12-dFC	14-3-3–GIGYF2(pS546)	GIGYF2-dependent translation repression	Preclinical (biochemical, cellular validation)

## Data Availability

No new data were created or analyzed in this study. Data sharing is not applicable to this article.

## References

[1] Stanton B.Z., Chory E.J., Crabtree G.R. (2018). Chemically induced proximity in biology and medicine. Science.

[2] Watson E.R., Novick S., Matyskiela M.E., Chamberlain P.P., de la Peña A.H., Zhu J., Tran E., Griffin P.R., Wertz I.E., Lander G.C. (2022). Molecular glue CELMoD compounds are regulators of cereblon conformation. Science.

[3] Zhao L., Zhao J., Zhong K., Tong A., Jia D. (2022). Targeted protein degradation: Mechanisms, strategies and application. Signal Transduct. Target. Ther..

[4] Henning N.J., Boike L., Spradlin J.N., Ward C.C., Liu G., Zhang E., Belcher B.P., Brittain S.M., Hesse M.J., Dovala D. (2022). Deubiquitinase-targeting chimeras for targeted protein stabilization. Nat. Chem. Biol..

[5] Kabir M., Sun N., Hu X., Martin T.C., Yi J., Zhong Y., Xiong Y., Kaniskan H., Gu W., Parsons R. (2023). Acetylation Targeting Chimera Enables Acetylation of the Tumor Suppressor p53. J. Am. Chem. Soc..

[6] Schreiber S.L. (2021). The Rise of Molecular Glues. Cell.

[7] Guo Z., Hong S.Y., Wang J., Rehan S., Liu W., Peng H., Das M., Li W., Bhat S., Peiffer B. (2019). Rapamycin-inspired macrocycles with new target specificity. Nat. Chem..

[8] Wolpin B.M., Wainberg Z.A., Hendifar A., Borad M.J., Pietrantonio F., Pant S., Hammel P., Cremolini C., Manji G.A., Oberstein P.E. (2026). Daraxonrasib, a RAS(ON) multi-selective inhibitor vs chemotherapy in previously treated metastatic pancreatic adenocarcinoma (mPDAC): Primary and final analysis from the phase 3 RASolute 302 study. J. Clin. Oncol..

[9] Yu S., Zhou L., Yang J., Zhang J., Lu W. (2025). Exploring nondegrading molecular glues for protein–protein interactions. Trends Biochem. Sci..

[10] Schulze C.J., Seamon K.J., Zhao Y., Yang Y.C., Cregg J., Kim D., Tomlinson A., Choy T.J., Wang Z., Sang B. (2023). Chemical remodeling of a cellular chaperone to target the active state of mutant KRAS. Science.

[11] Simanshu D.K., Nissley D.V., McCormick F. (2017). RAS Proteins and Their Regulators in Human Disease. Cell.

[12] Lu S., Jang H., Nussinov R., Zhang J. (2016). The Structural Basis of Oncogenic Mutations G12, G13 and Q61 in Small GTPase K-Ras4B. Sci. Rep..

[13] Prior I.A., Hood F.E., Hartley J.L. (2020). The Frequency of Ras Mutations in Cancer. Cancer Res..

[14] Cox A.D., Fesik S.W., Kimmelman A.C., Luo J., Der C.J. (2014). Drugging the undruggable RAS: Mission possible?. Nat. Rev. Drug. Discov..

[15] Stephen A.G., Esposito D., Bagni R.K., McCormick F. (2014). Dragging ras back in the ring. Cancer Cell.

[16] Jänne P.A., Riely G.J., Gadgeel S.M., Heist R.S., Ou S.I., Pacheco J.M., Johnson M.L., Sabari J.K., Leventakos K., Yau E. (2022). Adagrasib in Non-Small-Cell Lung Cancer Harboring a KRAS(G12C) Mutation. N. Engl. J. Med..

[17] Nakajima E.C., Drezner N., Li X., Mishra-Kalyani P.S., Liu Y., Zhao H., Bi Y., Liu J., Rahman A., Wearne E. (2022). FDA Approval Summary: Sotorasib for KRAS G12C-Mutated Metastatic NSCLC. Clin. Cancer Res..

[18] Janes M.R., Zhang J., Li L.S., Hansen R., Peters U., Guo X., Chen Y., Babbar A., Firdaus S.J., Darjania L. (2018). Targeting KRAS Mutant Cancers with a Covalent G12C-Specific Inhibitor. Cell.

[19] Wrighton K.H. (2023). Remodeling cyclophilin A to target KRAS. Nat. Cancer.

[20] Nokin M.J., Mira A., Patrucco E., Ricciuti B., Cousin S., Soubeyran I., San José S., Peirone S., Caizzi L., Vietti Michelina S. (2024). RAS-ON inhibition overcomes clinical resistance to KRAS G12C-OFF covalent blockade. Nat. Commun..

[21] Cregg J., Edwards A.V., Chang S., Lee B.J., Knox J.E., Tomlinson A.C.A., Marquez A., Liu Y., Freilich R., Aay N. (2025). Discovery of Daraxonrasib (RMC-6236), a Potent and Orally Bioavailable RAS(ON) Multi-selective, Noncovalent Tri-complex Inhibitor for the Treatment of Patients with Multiple RAS-Addicted Cancers. J. Med. Chem..

[22] Jiang J., Jiang L., Maldonato B.J., Wang Y., Holderfield M., Aronchik I., Winters I.P., Salman Z., Blaj C., Menard M. (2024). Translational and Therapeutic Evaluation of RAS-GTP Inhibition by RMC-6236 in RAS-Driven Cancers. Cancer Discov..

[23] O’Reilly E.M., Wainberg Z.A., Hendifar A.E., Borad M.J., Pietrantonio F., Pant S., Hammel P., Cremolini C., Manji G.A., Oberstein P.E. (2026). Daraxonrasib or Chemotherapy in Previously Treated Metastatic Pancreatic Cancer. N. Engl. J. Med..

[24] Cregg J., Pota K., Tomlinson A.C.A., Yano J., Marquez A., Liu Y., Schulze C.J., Seamon K.J., Holderfield M., Wei X. (2025). Discovery of Elironrasib (RMC-6291), a Potent and Orally Bioavailable, RAS(ON) G12C-Selective, Covalent Tricomplex Inhibitor for the Treatment of Patients with RAS G12C-Addicted Cancers. J. Med. Chem..

[25] Riess J., Haura E.B., Yaeger R., Johnson M., Luo J., Parikh A.R., Orr D., Punekar S.R., Papadopoulos K., Strickler J.H. (2026). Abstract CT021: Preliminary safety and clinical activity of zoldonrasib (RMC-9805), an oral, RAS(ON) G12D-selective, tri-complex inhibitor in patients with previously treated KRAS G12D non-small cell lung cancer (NSCLC). Cancer Res..

[26] (2026). Phase 1/1b, Multicenter, Open-Label, Study of RMC-5127 in Patients with Advanced KRAS G12V-Mutant Solid Tumors. https://clinicaltrials.gov/study/NCT07349537.

[27] Lew E.D., Brooun A., Lam J., Oh J., Sullivan T., Holub C., Bae J.H., Lin B., Qua B., Stromitis A. (2025). Abstract 390: ERAS-0015 is a pan-RAS molecular glue with best-in-class potential in RAS mutant solid tumors. Cancer Res..

[28] Yan F., Zhao J., Zhang J., Ren H., Le S., Zhou F., Lan J., Lu Q. (2025). Abstract 389: GFH276: A molecular glue panRAS(on) inhibitor with broad spectrum anti-tumor activities. Cancer Res..

[29] (2025). A Single-Arm, Open-Label, Multicenter Phase I/II Clinical Study of GFH276 in Patients with RAS-Mutant Advanced Solid Tumors. https://clinicaltrials.gov/study/NCT07198321.

[30] Liu H., Chen L., Meng Y., Huang H., Li M., Kang N., Feng Y., Liu Z., Li J., Lin K. (2026). Abstract 4570: HEC228032, an orally bioavailable molecular glue pan-RAS (ON) inhibitor with highly potent anti-tumor efficacy. Cancer Res..

[31] Zhang Z., Di X., Sun Q. (2026). Abstract 1830: SP09253, an orally bioavailable, highly potent RAS(ON) molecular glue inhibitor demonstrates robust anti-tumor activity in KRAS-mutant tumors. Cancer Res..

[32] Chen X., Wang L., Liu X., You Q., Li S., Wang L., Bi S., Jiang J., Bao J. (2026). Abstract 5778: RCZY-690: A tri-complex molecular glue pan-RAS (ON) inhibitor exhibiting best-in-class potential for RAS-addicted solid tumors. Cancer Res..

[33] Wu Y., Xie J., Huang J., Zhou X. (2025). Abstract 388: HZ-V068, an oral, highly potent pan-Ras molecular glue inhibitor demonstrated robust potency in pancreatic ductal adenocarcinoma and colorectal cancer models. Cancer Res..

[34] Wu Y., Feng B., Xie J., Wang P., Huang J., Hu X., Xu G., Zhou Y., Li J., Zhou X. (2026). Abstract LB345: HZ-V055, an oral, highly potent pan-Ras molecular glue inhibitor demonstrated robust potency in RasMut cancer models. Cancer Res..

[35] (2025). A Multicenter, Open Phase I/IIa Study to Evaluate the Safety, Tolerability, Pharmacokinetics, and Preliminary Antitumor Activity of JAB-23E73 in Patients with Advanced Solid Tumors Harboring KRAS Gene Alteration. https://clinicaltrials.gov/study/NCT06959615.

[36] Scheffzek K., Shivalingaiah G. (2019). Ras-Specific GTPase-Activating Proteins-Structures, Mechanisms, and Interactions. Cold Spring Harb. Perspect. Med..

[37] Ranđelović I., Nyíri K., Koppány G., Baranyi M., Tóvári J., Kigyós A., Tímár J., Vértessy B.G., Grolmusz V. (2024). Gluing GAP to RAS Mutants: A New Approach to an Old Problem in Cancer Drug Development. Int. J. Mol. Sci..

[38] Gerasimova A., Miller S., Chernoff J. (2026). Abstract 4521: Use of a p120 RasGAP glue to inhibit KRas in NF1-null cells. Cancer Res..

[39] Sang B., Ye L.F., Fu Z., Pourfarjam Y., Cuevas-Navarro A., Fan S., Hu F., Washington A., Rodriguez D.J., Vides A. (2026). Disrupted molecular glue complex drives RAS inhibitor resistance. Cell.

[40] Holderfield M., Lee B.J., Jiang J., Tomlinson A., Seamon K.J., Mira A., Patrucco E., Goodhart G., Dilly J., Gindin Y. (2024). Concurrent inhibition of oncogenic and wild-type RAS-GTP for cancer therapy. Nature.

[41] Wolpin B.M., Park W., Garrido-Laguna I., Spira A., Starodub A., Sommerhalder D., Punekar S.R., Barve M., Pelster M., Herzberg B. (2026). Daraxonrasib in Previously Treated Advanced RAS-Mutated Pancreatic Cancer. N. Engl. J. Med..

[42] Yaeger R., Corcoran R.B. (2019). Targeting Alterations in the RAF-MEK Pathway. Cancer Discov..

[43] Bahar M.E., Kim H.J., Kim D.R. (2023). Targeting the RAS/RAF/MAPK pathway for cancer therapy: From mechanism to clinical studies. Signal Transduct. Target. Ther..

[44] Gaire B., Orive-Ramos A., Adamopoulos C., Baars B., Desaunay M., Matenoglou E., Coma S., Gutierrez-Trejo N., Mohammed K., Aaronson S.A. (2026). Abstract 2948: A spatial-trapping mechanism of RAF by RAF/MEK glue enables full-dose combination with a pan-RAF inhibitor and drives potent, RAS-mutant tumor-selective MAPK and growth inhibition. Cancer Res..

[45] Banerjee S., Krebs M.G., Greystoke A., Garces A.I., Perez V.S., Terbuch A., Shinde R., Caldwell R., Grochot R., Rouhifard M. (2025). Defactinib with avutometinib in patients with solid tumors: The phase 1 FRAME trial. Nat. Med..

[46] (2020). A Phase 2 Study of Avutometinib (VS-6766) (Dual RAF/MEK Inhibitor) Alone and In Combination with Defactinib (FAK Inhibitor) in Recurrent Low-Grade Serous Ovarian Cancer (LGSOC). https://adisinsight.springer.com/trials/700320830.

[47] Banerjee S.N., Van Nieuwenhuysen E., Aghajanian C., D’Hondt V., Monk B.J., Clamp A., Prendergast E., Oaknin A., Ring K., Colombo N. (2025). Efficacy and Safety of Avutometinib ± Defactinib in Recurrent Low-Grade Serous Ovarian Cancer: Primary Analysis of ENGOT-OV60/GOG-3052/RAMP 201. J. Clin. Oncol..

[48] Ryan M.B., Quade B., Schenk N., Fang Z., Zingg M., Cohen S.E., Swalm B.M., Li C., Özen A., Ye C. (2024). The Pan-RAF-MEK Nondegrading Molecular Glue NST-628 Is a Potent and Brain-Penetrant Inhibitor of the RAS-MAPK Pathway with Activity Across Diverse RAS- and RAF-Driven Cancers. Cancer Discov..

[49] (2024). A Phase I, Open Label Single-Arm Two-Part Study to Investigate Safety, Pharmacokinetics, and Preliminary Efficacy of Pan-RAF/MEK Glue NST-628 Oral Tablets in Subject with Solid Tumors Harboring Genetic Alterations in the MAPK Pathway and with Other Solid Tumors. https://www.mdanderson.org/patients-family/diagnosis-treatment/clinical-trials/clinical-trials-index/clinical-trials-detail.ID2024-0403.html.

[50] Haines E., Burke M., Catterall R., De Jesus V., Hidalgo D., Li B., Manna J.D., Yang A., Cavanaugh J., Wessell S.R. (2026). The MEK-RAF molecular glue IK-595 has potent antitumor activity across RAS/MAPK pathway-altered cancers. Nat. Cancer.

[51] (2024). A First-in-Human (FiH) Study of IK-595, an Oral Dual MEK/RAF Inhibitor, in Patients with RAS-or RAF-altered Advanced Solid Tumors. https://www.patlynk.com/trial/NCT06270082.

[52] Tolcher A.W., Lakhani N.J., McKean M., Bashir B., Henry J.T., Vandross A.L., Spira A.I., Valerin J.B., Lingaraj T., Kim K. (2025). Abstract CT268: Preliminary results from a first-in-human study of IK-595, an oral MEK/RAF molecular glue, in patients with RAS- or RAF-altered advanced solid tumors. Cancer Res..

[53] Lavoie H., Sahmi M., Maisonneuve P., Marullo S.A., Thevakumaran N., Jin T., Kurinov I., Sicheri F., Therrien M. (2018). MEK drives BRAF activation through allosteric control of KSR proteins. Nature.

[54] Khan Z.M., Real A.M., Marsiglia W.M., Chow A., Duffy M.E., Yerabolu J.R., Scopton A.P., Dar A.C. (2020). Structural basis for the action of the drug trametinib at KSR-bound MEK. Nature.

[55] Jang D.M., Boxer K., Ha B.H., Tkacik E., Levitz T., Rawson S., Metivier R.J., Schmoker A., Jeon H., Eck M.J. (2025). Cryo-EM structures of CRAF/MEK1/14-3-3 complexes in autoinhibited and open-monomer states reveal features of RAF regulation. Nat. Commun..

[56] Konstantinidou M., Vickery H.R., Pennings M.A.M., Virta J.M., Luo S.Y., Visser E.J., Bannier S.D., Srikanth M., Cismoski S.Z., Young L.C. (2026). Modulation of the 14-3-3σ/C-RAF “Auto”inhibited Complex by Molecular Glues. J. Am. Chem. Soc..

[57] Virta J.M., Vickery H.R., Konstantinidou M., Crawford M.C., Pennings M.A.M., Ottmann C., Brunsveld L., Arkin M.R. (2026). Restoring the 14-3-3/CRAF regulatory interaction in Noonan syndrome using molecular glues. Proc. Natl. Acad. Sci. USA.

[58] Garvie C.W., Wu X., Papanastasiou M., Lee S., Fuller J., Schnitzler G.R., Horner S.W., Baker A., Zhang T., Mullahoo J.P. (2021). Structure of PDE3A-SLFN12 complex reveals requirements for activation of SLFN12 RNase. Nat. Commun..

[59] de Waal L., Lewis T.A., Rees M.G., Tsherniak A., Wu X., Choi P.S., Gechijian L., Hartigan C., Faloon P.W., Hickey M.J. (2016). Identification of cancer-cytotoxic modulators of PDE3A by predictive chemogenomics. Nat. Chem. Biol..

[60] Lewis T.A., de Waal L., Wu X., Youngsaye W., Wengner A., Kopitz C., Lange M., Gradl S., Ellermann M., Lienau P. (2019). Optimization of PDE3A Modulators for SLFN12-Dependent Cancer Cell Killing. ACS Med. Chem. Lett..

[61] Lewis T.A., Ellermann M., Kopitz C., Lange M., de Waal L., Wu X., Tersteegen A., Denner K., Lienau P., Kaulfuss S. (2024). Discovery of BAY 2666605, a Molecular Glue for PDE3A and SLFN12. ACS Med. Chem. Lett..

[62] Takaki E.O., Kiyono K., Obuchi Y., Yamauchi T., Watanabe T., Matsumoto H., Karimine M., Kuniyoshi Y., Nishikori S., Yokoyama F. (2024). A PDE3A-SLFN12 Molecular Glue Exhibits Significant Antitumor Activity in TKI-Resistant Gastrointestinal Stromal Tumors. Clin. Cancer Res..

[63] Papadopoulos K.P., McKean M., Goldoni S., Genvresse I., Garrido M.F., Li R., Wilkinson G., Kneip C., Yap T.A. (2024). First-in-Human Dose-Escalation Study of the First-in-Class PDE3A-SLFN12 Complex Inducer BAY 2666605 in Patients with Advanced Solid Tumors Coexpressing SLFN12 and PDE3A. Clin. Cancer Res..

[64] Vogler M., Braun Y., Smith V.M., Westhoff M.A., Pereira R.S., Pieper N.M., Anders M., Callens M., Vervliet T., Abbas M. (2025). The BCL2 family: From apoptosis mechanisms to new advances in targeted therapy. Signal Transduct. Target. Ther..

[65] Yuda J., Will C., Phillips D.C., Abraham L., Alvey C., Avigdor A., Buck W., Besenhofer L., Boghaert E., Cheng D. (2023). Selective MCL-1 inhibitor ABBV-467 is efficacious in tumor models but is associated with cardiac troponin increases in patients. Commun. Med..

[66] Li F., Zhang M., Liu C., Cheng J., Yang Y., Peng X., Li Z., Cai W., Yu H., Wu J. (2025). De novo discovery of a molecular glue-like macrocyclic peptide that induces MCL1 homodimerization. Proc. Natl. Acad. Sci. USA.

[67] Hanahan D. (2022). Hallmarks of Cancer: New Dimensions. Cancer Discov..

[68] Zhao M., Mishra L., Deng C.X. (2018). The role of TGF-β/SMAD4 signaling in cancer. Int. J. Biol. Sci..

[69] Tang C., Mo X., Niu Q., Wahafu A., Yang X., Qui M., Ivanov A.A., Du Y., Fu H. (2021). Hypomorph mutation-directed small-molecule protein-protein interaction inducers to restore mutant SMAD4-suppressed TGF-β signaling. Cell Chem. Biol..

[70] Kumari P., Oum Y.H., Miller E.J., Qui M., Du Y., Mou H., Singh R., Fu H., Mo X. (2026). Neo-Cysteine Molecular Glues for Targeting Mutated SMAD4 Protein. Angew. Chem. Int. Ed..

[71] Haanen T.J., O’Connor C.M., Narla G. (2022). Biased holoenzyme assembly of protein phosphatase 2A (PP2A): From cancer to small molecules. J. Biol. Chem..

[72] Leonard D., Huang W., Izadmehr S., O’Connor C.M., Wiredja D.D., Wang Z., Zaware N., Chen Y., Schlatzer D.M., Kiselar J. (2020). Selective PP2A Enhancement through Biased Heterotrimer Stabilization. Cell.

[73] Vit G., Duro J., Rajendraprasad G., Hertz E.P.T., Holland L.K.K., Weisser M.B., McEwan B.C., Lopez-Mendez B., Sotelo-Parrilla P., Jeyaprakash A.A. (2022). Chemogenetic profiling reveals PP2A-independent cytotoxicity of proposed PP2A activators iHAP1 and DT-061. EMBO J..

[74] Raines B., Tseng-Rogenski S., Dowdican A.C., Peris I., Hinderman M., Zawacki K.P., Barrie K., Hodges Onishi G., Dymond A.M., Luther T.K. (2025). A PP2A molecular glue overcomes RAS/MAPK inhibitor resistance in KRAS-mutant non-small cell lung cancer. J. Clin. Investig..

[75] Wang H., Guo M., Wei H., Chen Y. (2023). Targeting p53 pathways: Mechanisms, structures, and advances in therapy. Signal Transduct. Target. Ther..

[76] Isobe Y., Okumura M., McGregor L.M., Brittain S.M., Jones M.D., Liang X., White R., Forrester W., McKenna J.M., Tallarico J.A. (2020). Manumycin polyketides act as molecular glues between UBR7 and P53. Nat. Chem. Biol..

[77] Li Z., Wang Z., Zhong C., Zhang H., Liu R., An P., Ma Z., Lu J., Pan C., Zhang Z. (2024). P53 upregulation by USP7-engaging molecular glues. Sci. Bull..

[78] Sung J., Seo Y., Lee J.-Y. (2026). From undruggable to degradable: Strategic expansion of targeted protein degradation in cancer therapy. Mol. Ther..

[79] Słabicki M., Yoon H., Koeppel J., Nitsch L., Roy Burman S.S., Di Genua C., Donovan K.A., Sperling A.S., Hunkeler M., Tsai J.M. (2020). Small-molecule-induced polymerization triggers degradation of BCL6. Nature.

[80] Langan C., Brown N.E., Perria B.G., Gatzeva-Topalova P., Jones B.D., Kindler L.J., Blosser W.D., Metivier R.J., Fischer E.S., Gong X. (2026). Abstract 4594: LY4584180, a novel BCL6 molecular glue, demonstrates antitumor efficacy in preclinical models of B Cell NHL. Cancer Res..

[81] Duong T.N., Pandji E., Shao Q., Nomura D.K. (2025). Discovery of Non-Degradative Covalent Molecular Glues for Transcriptional Reprogramming. bioRxiv.

[82] De Vries-van Leeuwen I.J., da Costa Pereira D., Flach K.D., Piersma S.R., Haase C., Bier D., Yalcin Z., Michalides R., Feenstra K.A., Jiménez C.R. (2013). Interaction of 14-3-3 proteins with the estrogen receptor alpha F domain provides a drug target interface. Proc. Natl. Acad. Sci. USA.

[83] Konstantinidou M., Visser E.J., Vandenboorn E., Chen S., Jaishankar P., Overmans M., Dutta S., Neitz R.J., Renslo A.R., Ottmann C. (2023). Structure-Based Optimization of Covalent, Small-Molecule Stabilizers of the 14-3-3σ/ERα Protein–Protein Interaction from Nonselective Fragments. J. Am. Chem. Soc..

[84] Konstantinidou M., Zingiridis M., Pennings M.A.M., Fragkiadakis M., Virta J.M., Revalde J.L., Visser E.J., Ottmann C., Brunsveld L., Neochoritis C.G. (2025). Scaffold-hopping for molecular glues targeting the 14-3-3/ERα complex. Nat. Commun..

[85] Shao Q., Duong T.N., Park I., Orr L.M., Nomura D.K. (2024). Targeted Protein Localization by Covalent 14–3–3 Recruitment. J. Am. Chem. Soc..

[86] Liu Y., Hua H., Cao Y., Li M., Zhang H., Du S., Liu J., Luo T., Jiang Y. (2025). Mechanism by which the molecular glue-like verteporfin induces IRE1α dimerization and activation to synergize with AKT inhibition in breast cancer. Cell Chem. Biol..

[87] Ogino N., Masuda R., Igaue S., Arita M., Matsumura A., Ieda Y., Muroi M., Matsumoto K., Nakagawa R., Higuchi Y. (2026). Diterpene Molecular Glue Stabilizes Protein-Protein Interactions of a Disordered Phosphoprotein that Controls Translational Repression. JACS Au.

[88] Kawakami K., Hattori M., Inoue T., Maruyama Y., Ohkanda J., Kato N., Tongu M., Yamada T., Akimoto M., Takenaga K. (2012). A novel fusicoccin derivative preferentially targets hypoxic tumor cells and inhibits tumor growth in xenografts. Anticancer Agents Med. Chem..

[89] Shi J., Zhang Y., Zhao N., Seki E., Ma L., Kocic G., Li X., Terzić J., Zheng T. (2026). Precision targeting of STING: Challenges, innovations, and clinical outlook for cancer therapy. Innovation.

[90] Li J., Canham S.M., Wu H., Henault M., Chen L., Liu G., Chen Y., Yu G., Miller H.R., Hornak V. (2024). Activation of human STING by a molecular glue-like compound. Nat. Chem. Biol..

[91] Yuan L., Ma X., Yang Y., Qu Y., Li X., Zhu X., Ma W., Duan J., Xue J., Yang H. (2023). Phosphoantigens glue butyrophilin 3A1 and 2A1 to activate Vγ9Vδ2 T cells. Nature.

[92] Zhu Y., Gao W., Zheng J., Bai Y., Tian X., Huang T., Lu Z., Dong D., Zhang A., Guo C. (2025). Phosphoantigen-induced inside-out stabilization of butyrophilin receptor complexes drives dimerization-dependent γδ TCR activation. Immunity.

[93] Bennouna J., Levy V., Sicard H., Senellart H., Audrain M., Hiret S., Rolland F., Bruzzoni-Giovanelli H., Rimbert M., Galéa C. (2010). Phase I study of bromohydrin pyrophosphate (BrHPP, IPH 1101), a Vgamma9Vdelta2 T lymphocyte agonist in patients with solid tumors. Cancer Immunol. Immunother..

[94] Bonnafous C., Faria B., Ingoure S., Benech P., Joly F., Halfon P., Bouzidi M., Girre J.-P., Sicard H. (2009). IPH1101 FIRST GENERATION γδT CELL AGONIST. EX VIVO AND IN VIVO ANTIVIRAL FUNCTIONS OF γδT CELLS FROM CHRONICALLY INFECTED HCV PATIENTS TREATED IN PHASE 2 STUDY: 1583. Hepatology.

[95] Wolter M., de Vink P., Neves J.F., Srdanović S., Higuchi Y., Kato N., Wilson A., Landrieu I., Brunsveld L., Ottmann C. (2020). Selectivity via Cooperativity: Preferential Stabilization of the p65/14-3-3 Interaction with Semisynthetic Natural Products. J. Am. Chem. Soc..

[96] Mao H., Zhao X., Sun S.-c. (2025). NF-κB in inflammation and cancer. Cell. Mol. Immunol..

[97] Watson P.J., Fairall L., Santos G.M., Schwabe J.W. (2012). Structure of HDAC3 bound to co-repressor and inositol tetraphosphate. Nature.

[98] Chatterjee S., Sin Z., Tran N., Vierra L., Shukla A., Tran T., Koshkaryan G., Ritter K., Su X.B., Ragsac S.J. (2026). Phytic acid (InsP(6)) activates HDAC3 epigenetic axis to maintain intestinal barrier function. Nat. Commun..

[99] Lau J., Bloch P., Schäffer L., Pettersson I., Spetzler J., Kofoed J., Madsen K., Knudsen L.B., McGuire J., Steensgaard D.B. (2015). Discovery of the Once-Weekly Glucagon-Like Peptide-1 (GLP-1) Analogue Semaglutide. J. Med. Chem..

[100] Bueno A.B., Sun B., Willard F.S., Feng D., Ho J.D., Wainscott D.B., Showalter A.D., Vieth M., Chen Q., Stutsman C. (2020). Structural insights into probe-dependent positive allosterism of the GLP-1 receptor. Nat. Chem. Biol..

[101] Willard F.S., Wainscott D.B., Showalter A.D., Stutsman C., Ma W., Cardona G.R., Zink R.W., Corkins C.M., Chen Q., Yumibe N. (2021). Discovery of an Orally Efficacious Positive Allosteric Modulator of the Glucagon-like Peptide-1 Receptor. J. Med. Chem..

[102] Li J., Li G., Mai Y., Liu X., Yang D., Zhou Q., Wang M.-W. (2024). Molecular basis of enhanced GLP-1 signaling mediated by GLP-1(9–36) in conjunction with LSN3318839. Acta Pharm. Sin. B.

[103] Xin Y., Liu S., Liu Y., Qian Z., Liu H., Zhang B., Guo T., Thompson G.J., Stevens R.C., Sharpless K.B. (2023). Affinity selection of double-click triazole libraries for rapid discovery of allosteric modulators for GLP-1 receptor. Proc. Natl. Acad. Sci. USA.

[104] Katz L.S., Visser E.J., Plitzko K.F., Pennings M.A.M., Cossar P.J., Tse I.L., Kaiser M., Brunsveld L., Ottmann C., Scott D.K. (2024). Molecular glues of the regulatory ChREBP/14-3-3 complex protect beta cells from glucolipotoxicity. bioRxiv.

[105] Czyzyk D., Yan W., Messing S., Gillette W., Tsuji T., Yamaguchi M., Furuzono S., Turner D.M., Esposito D., Nissley D.V. (2025). Structural insights into isoform-specific RAS-PI3Kα interactions and the role of RAS in PI3Kα activation. Nat. Commun..

[106] Terayama K., Furuzono S., Fer N., Yan W., Young L.C., Czyzyk D.J., Goldstein de Salazar R., Sasaki M., Uozumi A., Konishi M. (2025). Molecular glues that facilitate RAS binding to PI3Kα promote glucose uptake without insulin. Science.

[107] French J.A., Lawson J.A., Yapici Z., Ikeda H., Polster T., Nabbout R., Curatolo P., de Vries P.J., Dlugos D.J., Berkowitz N. (2016). Adjunctive everolimus therapy for treatment-resistant focal-onset seizures associated with tuberous sclerosis (EXIST-3): A phase 3, randomised, double-blind, placebo-controlled study. Lancet.

[108] Sidrauski C., Acosta-Alvear D., Khoutorsky A., Vedantham P., Hearn B.R., Li H., Gamache K., Gallagher C.M., Ang K.K., Wilson C. (2013). Pharmacological brake-release of mRNA translation enhances cognitive memory. eLife.

[109] Tsai J.C., Miller-Vedam L.E., Anand A.A., Jaishankar P., Nguyen H.C., Renslo A.R., Frost A., Walter P. (2018). Structure of the nucleotide exchange factor eIF2B reveals mechanism of memory-enhancing molecule. Science.

[110] Zyryanova A.F., Kashiwagi K., Rato C., Harding H.P., Crespillo-Casado A., Perera L.A., Sakamoto A., Nishimoto M., Yonemochi M., Shirouzu M. (2021). ISRIB Blunts the Integrated Stress Response by Allosterically Antagonising the Inhibitory Effect of Phosphorylated eIF2 on eIF2B. Mol. Cell.

[111] Chou A., Krukowski K., Jopson T., Zhu P.J., Costa-Mattioli M., Walter P., Rosi S. (2017). Inhibition of the integrated stress response reverses cognitive deficits after traumatic brain injury. Proc. Natl. Acad. Sci. USA.

[112] Sidrauski C., McGeachy A.M., Ingolia N.T., Walter P. (2015). The small molecule ISRIB reverses the effects of eIF2α phosphorylation on translation and stress granule assembly. eLife.

[113] Flores B.N., Yu S.B., Cohen I.V., Fanok M.H., Luan W., Maciuca R.D., Sun L.D., Tsai R.M., Vissers M., Smits L. (2025). Investigational eIF2B activator DNL343 modulates the integrated stress response in preclinical models of TDP-43 pathology and individuals with ALS in a randomized clinical trial. Nat. Commun..

[114] Zhu P.J., Khatiwada S., Cui Y., Reineke L.C., Dooling S.W., Kim J.J., Li W., Walter P., Costa-Mattioli M. (2019). Activation of the ISR mediates the behavioral and neurophysiological abnormalities in Down syndrome. Science.

[115] Halliday M., Radford H., Sekine Y., Moreno J., Verity N., le Quesne J., Ortori C.A., Barrett D.A., Fromont C., Fischer P.M. (2015). Partial restoration of protein synthesis rates by the small molecule ISRIB prevents neurodegeneration without pancreatic toxicity. Cell Death Dis..

[116] Krukowski K., Nolan A., Frias E.S., Boone M., Ureta G., Grue K., Paladini M.S., Elizarraras E., Delgado L., Bernales S. (2020). Small molecule cognitive enhancer reverses age-related memory decline in mice. eLife.

[117] Denali Therapeutics Inc (2023). HEALEY ALS Platform Trial—Regimen G DNL343. https://clinicaltrials.gov/study/NCT05842941.

[118] (2025). Single Patient Investigational Treatment for Cree Leukoencephalopathy (Vanishing White Matter Disease). https://clinicaltrials.gov/study/NCT07300397.

[119] Adelman J.P., Maylie J., Sah P. (2012). Small-conductance Ca2+-activated K+ channels: Form and function. Annu. Rev. Physiol..

[120] Zhang M., Pascal J.M., Schumann M., Armen R.S., Zhang J.F. (2012). Identification of the functional binding pocket for compounds targeting small-conductance Ca^2+^-activated potassium channels. Nat. Commun..

[121] Zhang M., Nam Y.W., Ramanishka A., Xu Y., Yasuda R.M., Im D., Cui M., Chandy G., Wulff H. (2025). Structural basis for the subtype-selectivity of K(Ca)2.2 channel activators. Res. Sq..

[122] Shakkottai V.G., do Carmo Costa M., Dell’Orco J.M., Sankaranarayanan A., Wulff H., Paulson H.L. (2011). Early changes in cerebellar physiology accompany motor dysfunction in the polyglutamine disease spinocerebellar ataxia type 3. J. Neurosci..

[123] Chen T., Zhu J., Hang C.H., Wang Y.H. (2019). The Potassium SK Channel Activator NS309 Protects Against Experimental Traumatic Brain Injury Through Anti-Inflammatory and Immunomodulatory Mechanisms. Front. Pharmacol..

[124] (2018). A Phase 2a Open-Label Study to Evaluate the Safety, Tolerability and Efficacy of CAD-1883 Oral Treatment in Adults with Essential Tremor. https://clinicaltrials.gov/study/NCT03688685.

[125] Zhu Y.X., Yang Z., Li L., Chen Z.K., Zhuo F.F., Wang Y.H., Liu Z.P., Han B., Yu W., Tu P.F. (2026). Molecular glue neferine induces BAP31 homodimerisation to disrupt endoplasmic reticulum-mitochondria crosstalk for anti-neuroinflammation in ischaemic stroke. Br. J. Pharmacol..

[126] Mecozzi V.J., Berman D.E., Simoes S., Vetanovetz C., Awal M.R., Patel V.M., Schneider R.T., Petsko G.A., Ringe D., Small S.A. (2014). Pharmacological chaperones stabilize retromer to limit APP processing. Nat. Chem. Biol..

[127] Mishra S., Knupp A., Kinoshita C., Williams C.A., Rose S.E., Martinez R., Theofilas P., Young J.E. (2023). Pharmacologic enhancement of retromer rescues endosomal pathology induced by defects in the Alzheimer’s gene *SORL1*. Stem Cell Rep..

[128] Young J.E., Fong L.K., Frankowski H., Petsko G.A., Small S.A., Goldstein L.S.B. (2018). Stabilizing the Retromer Complex in a Human Stem Cell Model of Alzheimer’s Disease Reduces TAU Phosphorylation Independently of Amyloid Precursor Protein. Stem Cell Rep..

[129] Muzio L., Sirtori R., Gornati D., Eleuteri S., Fossaghi A., Brancaccio D., Manzoni L., Ottoboni L., Feo L.D., Quattrini A. (2020). Retromer stabilization results in neuroprotection in a model of Amyotrophic Lateral Sclerosis. Nat. Commun..

[130] Christ F., Voet A., Marchand A., Nicolet S., Desimmie B.A., Marchand D., Bardiot D., Van der Veken N.J., Van Remoortel B., Strelkov S.V. (2010). Rational design of small-molecule inhibitors of the LEDGF/p75-integrase interaction and HIV replication. Nat. Chem. Biol..

[131] Singer M.R., Dinh T., Levintov L., Annamalai A.S., Rey J.S., Briganti L., Cook N.J., Pye V.E., Taylor I.A., Kim K. (2023). The Drug-Induced Interface That Drives HIV-1 Integrase Hypermultimerization and Loss of Function. mBio.

[132] Bonnard D., Le Rouzic E., Singer M.R., Yu Z., Le Strat F., Batisse C., Batisse J., Amadori C., Chasset S., Pye V.E. (2023). Biological and Structural Analyses of New Potent Allosteric Inhibitors of HIV-1 Integrase. Antimicrob. Agents Chemother..

[133] Bruggemans A., Vansant G., Balakrishnan M., Mitchell M.L., Cai R., Christ F., Debyser Z. (2023). GS-9822, a preclinical LEDGIN candidate, displays a block-and-lock phenotype in cell culture. Antimicrob. Agents Chemother..

[134] Kessl J.J., Kutluay S.B., Townsend D., Rebensburg S., Slaughter A., Larue R.C., Shkriabai N., Bakouche N., Fuchs J.R., Bieniasz P.D. (2016). HIV-1 Integrase Binds the Viral RNA Genome and Is Essential during Virion Morphogenesis. Cell.

[135] (2023). A Phase 2a, Randomized, Double-Blinded, Placebo-Controlled, Multicenter Study to Investigate the Antiviral Effect, Safety, Tolerability, and Pharmacokinetics of STP0404 in Adults With HIV-1 Infection. https://clinicaltrials.gov/study/NCT05869643.

[136] Ramgopal M., Benson P., Williamson J.C., Berhe M., Ruane P., Casanas B., DeJesus E., Lucasti C., Meng X., Kim U.-I. (2026). 172. The First Proof-of-Concept Clinical Trial of an HIV-1 Allosteric Integrase Inhibitor, Pirmitegravir (STP0404). Open Forum Infect. Dis..

[137] Venn Life Sciences (2018). A Single Ascending Dose Trial Investigating the Safety, Tolerability and Pharmacokinetics of Orally Administered BDM-2 in Healthy Male Subjects. https://clinicaltrials.gov/study/NCT03634085.

[138] Dinh T., Tber Z., Rey J.S., Mengshetti S., Annamalai A.S., Haney R., Briganti L., Amblard F., Fuchs J.R., Cherepanov P. (2024). The structural and mechanistic bases for the viral resistance to allosteric HIV-1 integrase inhibitor pirmitegravir. bioRxiv.

[139] Roberts R.A., Campbell R.A., Sikakana P., Sadler C., Osier M., Xu Y., Feng J.Y., Mitchell M., Sakowicz R., Chester A. (2021). Species-Specific Urothelial Toxicity With an Anti-HIV Noncatalytic Site Integrase Inhibitor (NCINI) Is Related to Unusual pH-Dependent Physicochemical Changes. Toxicol. Sci..

[140] Vernuccio R., Martínez León A., Poojari C.S., Buchrieser J., Selverian C.N., Jaleta Y., Meola A., Guivel-Benhassine F., Porrot F., Haouz A. (2025). Structural insights into tecovirimat antiviral activity and poxvirus resistance. Nat. Microbiol..

[141] Yu P.A., Elmor R., Muhammad K., Yu Y.C., Rao A.K. (2024). Tecovirimat Use under Expanded Access to Treat Mpox in the United States, 2022-2023. NEJM Evid..

[142] Smith T.G., Gigante C.M., Wynn N.T., Matheny A., Davidson W., Yang Y., Condori R.E., O’Connell K., Kovar L., Williams T.L. (2023). Tecovirimat Resistance in Mpox Patients, United States, 2022-2023. Emerg. Infect. Dis..

[143] Qiu Y., Xu Y., Zhang Y., Zhou H., Deng Y.Q., Li X.F., Miao M., Zhang Q., Zhong B., Hu Y. (2017). Human Virus-Derived Small RNAs Can Confer Antiviral Immunity in Mammals. Immunity.

[144] Fang Y., Xie X., Fan H., Wu B., Wang A., Dai W., Liu Z., Li J., Tong H., Li J. (2026). Rational Design of Broad-Spectrum Anti-Enteroviral Molecular Glues Targeting Enteroviral RNAi Suppressors. Adv. Sci..

[145] Chana C.K., Ben Makhlouf I., Kim J., Yu A.J.Y., Moatti N., Orlicky S., Wong C.J., Baronijan L., Tang X., Mao D. (2026). The molecular glue CLEO4-88 inhibits the ACAA1 thiolase by induced binding to GID4. Nat. Chem. Biol..

[146] Peng W.T., Jin X., Xu X.E., Yang Y.S., Ma D., Shao Z.M., Jiang Y.Z. (2023). Inhibition of ACAA1 Restrains Proliferation and Potentiates the Response to CDK4/6 Inhibitors in Triple-Negative Breast Cancer. Cancer Res..

[147] Lee H., Kang M., Sim S.H., Kang J.H., Choi W., Chun J.W., Hong W., Kim C., Ham W., Park J.H. (2025). ACAA1 knockout increases the survival rate of KPC mice by activating autophagy. Mol. Metab..

[148] Lang T., Xiao P., Hua S., Liang X., Yang Y. (2025). Turning off the ferroptosis switch: ACAA1-Driven PI3K/AKT/Nrf2 signaling as a novel driver of endometrial cancer progression. Free Radic. Biol. Med..

[149] Zandi T.A., Romanowski M.J., Viscomi J.S., Gunderson K., Tan Z.Y., Tong B., Bonazzi S., Zécri F.J., Schreiber S.L., Michaud G.A. (2026). Discovery of molecular glues that bind FKBP12 and structurally distinct targets using DNA-encoded libraries. Nat. Commun..

[150] Ware M.L., Leace D.M., Qu Z., Schaefer Q., Vaidya S.D., Horvath M.L., Li Z., Bai Y., Zhang Z.-Y., Hsu K.-L. (2026). Protein tyrosine phosphatase inactivation by electrophilic tyrosine modification. Chem. Sci..

[151] Shen J., Peddada T.N., Komolov K.E., De Pascali F., Garces A.M., Wang H., Lerch M.T., Benovic J.L., Xu J., Kobilka B.K. (2025). A biased allosteric modulator functions as a molecular glue to induce β(2)AR dimerization. bioRxiv.

[152] Pahil K.S., Gilman M.S.A., Baidin V., Clairfeuille T., Mattei P., Bieniossek C., Dey F., Muri D., Baettig R., Lobritz M. (2024). A new antibiotic traps lipopolysaccharide in its intermembrane transporter. Nature.

[153] Zampaloni C., Mattei P., Bleicher K., Winther L., Thäte C., Bucher C., Adam J.-M., Alanine A., Amrein K.E., Baidin V. (2024). A novel antibiotic class targeting the lipopolysaccharide transporter. Nature.

[154] (2022). A Multicenter, Single-Dose, Uncontrolled, Open-Label, One Group Study to Investigate the Pharmacokinetics of RO7223280 in Critically Ill Patients With Bacterial Infections. https://clinicaltrials.gov/study/NCT05614895.

[155] Iwasaki S., Floor S.N., Ingolia N.T. (2016). Rocaglates convert DEAD-box protein eIF4A into a sequence-selective translational repressor. Nature.

[156] Kuzuoglu-Ozturk D., Nguyen H.G., Xue L., Figueredo E., Subramanyam V., Liu I., Bonitto K., Noronha A., Dabrowska A., Cowan J.E. (2025). Small-molecule RNA therapeutics to target prostate cancer. Cancer Cell.

[157] (2019). A Phase 1-2 Dose-Escalation and Cohort-Expansion Study of Intravenous Zotatifin (eFT226) in Subjects with Selected Advanced Solid Tumor Malignancies. https://clinicaltrials.gov/study/NCT04092673.

